# Age-specific associations between blood pressure and cardiovascular disease, kidney disease, and death among individuals with type 2 diabetes: a population-based cohort study

**DOI:** 10.1186/s12933-025-03072-1

**Published:** 2026-01-28

**Authors:** Edith W. K. Chow, Yingnan Fan, Hongjiang Wu, Eric S. H. Lau, Aimin Yang, Elaine Chow, Alice P. S. Kong, Ronald C. W. Ma, Juliana C. N. Chan, Andrea O. Y. Luk

**Affiliations:** 1 Department of Medicine and Therapeutics, Prince of Wales Hospital, The Chinese University of Hong Kong, Sha Tin, Hong Kong Special Administrative Region People’s Republic of China; 2 Li Ka Shing Institute of Health Sciences, The Chinese University of Hong Kong, Sha Tin, Hong Kong Special Administrative Region People’s Republic of China; 3 Hong Kong Institute of Diabetes and Obesity, The Chinese University of Hong Kong, Sha Tin, Hong Kong Special Administrative Region People’s Republic of China; 4 Phase 1 Clinical Trial Centre, The Chinese University of Hong Kong, Sha Tin, Hong Kong Special Administrative Region People’s Republic of China

**Keywords:** Blood pressure, Cardiovascular disease, Kidney failure, All-cause death, Age, Type 2 diabetes

## Abstract

**Background:**

High blood pressure(BP) is a modifiable risk factor for premature mortality, adverse cardiovascular outcomes and kidney diseases in individuals with type 2 diabetes(T2D). We studied the age-specific associations between BP and incident cardiovascular disease(CVD), chronic kidney disease(CKD), kidney failure, and all-cause death among individuals with T2D.

**Methods:**

We included individuals with T2D who underwent structured diabetes assessment between 2000 and 2022 in Hong Kong Special Administrative Region, People's Republic of China. Participants were stratified by baseline age(18–44 years, 45–59 years, 60–74 years, and 75 years or older). Cox proportional hazard model was used to estimate hazard ratios for the risk of incident CVD, CKD, kidney failure and all-cause death associated with systolic blood pressure(SBP) and diastolic blood pressure(DBP) categories, referenced to SBP 120–129 mmHg and DBP 70–79 mmHg within each age stratum. Non-linear association between SBP/ DBP and clinical outcomes was modelled using restricted cubic splines(RCS).

**Results:**

We included 429,740 individuals with T2D (mean age 61.9 years, 52.7% men, 75.3% pre-existing hypertension). SBP above 120–129 mmHg and DBP above 70–79 mmHg were associated with a proportional increase in risks for CVD, CKD, kidney failure and death across ages adjusted for demographics, diabetes duration, BMI, smoking status, HbA1c, lipids, albuminuria, history of CVD, CKD and use of BP-lowering medications. The strength of the risk associations was greatest in youngest age group and declined with increasing age. Among individual components of CVD, risks conferred by an incremental increase in SBP or DBP were high for hemorrhagic stroke. A 10 mmHg or 1-SD increase in SBP / DBP conferred a 1.2 to 1.5-fold increase in hazards for hemorrhagic stroke among individuals aged 18–44 years (*p*-interaction < 0.001). RCS indicated variable linear and nonlinear associations between SBP/DBP and CVD, kidney disease, or death across age categories.

**Conclusions:**

We observed heterogeneity in the relationship between BP and various clinical outcomes across ages in a diabetes population. Risk associations were strongest among young individuals, emphasising the importance of BP management in this population.

**Graphical abstract:**

**Figure:**
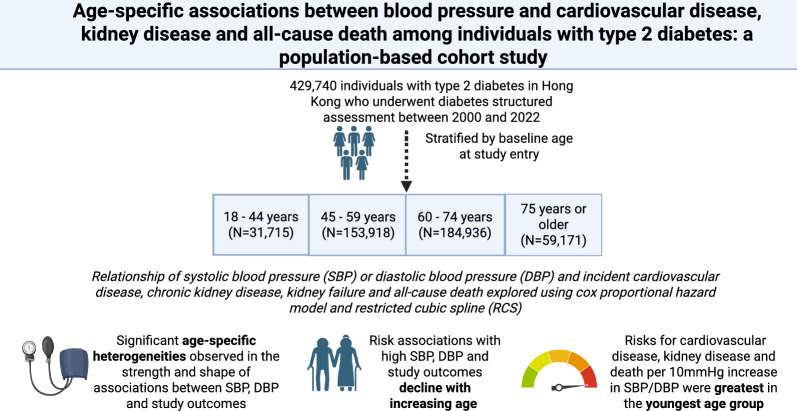
Created in BioRender. C, E. (2026) https://BioRender.com/zn17wcd

**Supplementary Information:**

The online version contains supplementary material available at 10.1186/s12933-025-03072-1.

## Research insights

**What is currently known about this topic?**

- Hypertension is a leading modifiable cardiovascular risk factor.
- Type 2 diabetes and hypertension commonly co-occur.
- There are age-dependent changes in blood pressure(BP) reflected in longitudinal studies.

**What is the key research question?**

- Does risk association between BP and diabetes outcomes change with age?

**What is new?**

- Age-specific heterogeneities exist in risk associations between BP and cardiovascular and kidney outcomes.

**How might this study influence clinical practice?**

- Findings highlight impact of high BP in young individuals with type 2 diabetes.

## Background

The Global Cardiovascular Risk Consortium reported that five modifiable cardiovascular risk factors(body mass index[BMI], systolic blood pressure [SBP], non-HDL cholesterol, smoking, diabetes) accounted for over 50% of the global burden of cardiovascular disease (CVD) [1]. Of these risk factors, improvement of hypertension in mid-life was associated with the most additional life-time years free of CVD [2]. Type 2 diabetes (T2D) is characterised by clustering of cardiovascular risk factors, with hypertension being prevalent in 54% to over 80% of individuals with diabetes in Asia [3, 4]. The risks of microvascular and macrovascular complications were higher in those with comorbid hypertension and diabetes, with studies reporting 1.5–1.9-fold higher hazards for any CVD and related death [5], and 2.8-fold higher hazards for incident chronic kidney disease(CKD), compared to those with diabetes alone [6].

The rise in blood pressure (BP) with ageing is well documented [7]. SBP increases linearly with age, while diastolic blood pressure (DBP) is characterised by an early rise and late fall after age 50 to 60 years [7]. Although it is established that high BP is associated with adverse cardiovascular and kidney outcomes, it is less well understood how these risks may differ by age. Most population-based analyses have demonstrated differential risk associations between high BP and cardiovascular outcomes across ages in the general population [8–10]. However, few studies have evaluated age-specific associations between BP, cardiovascular and kidney outcomes in a population with diabetes. A retrospective cohort study of individuals with diabetes investigated the relationship between usual SBP and CVD by age categories, but the risk associations with DBP were not assessed [11]. Since SBP and DBP represent distinct physiologies and age-related trajectories [7], separate analysis of SBP and DBP pressure may reveal meaningful differences in risk associations.

In this study, we aimed to investigate the age-specific associations between SBP, DBP with CVD, kidney diseases, and all-cause death in individuals with T2D. We hypothesised that heterogeneities by age exist in the risk associations between BP and clinical outcomes, which may have important implications for individualising hypertension management.

## Methods

### Data source and study cohort

The Hong Kong Hospital Authority (HA) is the statutory body that governs the public hospitals and most of the outpatient clinics in Hong Kong Special Administrative Region, People's Republic of China. Approximately 90% of the individuals with diabetes are treated under the public healthcare system in Hong Kong [12]. The Risk Assessment and Management Program-Diabetes Mellitus(RAMP-DM) is a structured assessment program offered to all individuals with diabetes [13]. Data from RAMP-DM have been used in other population-based epidemiological studies that reported diabetes-related outcomes [14, 15]. This study was reported according to the Strengthening the Reporting of Observational Studies in Epidemiology (STROBE) guidelines.

The study cohort consisted of individuals with T2D aged ≥ 18 years who participated in RAMP-DM between 1 January 2000 and 28 February 2022. The diagnosis of T2D was documented by the referring physician and confirmed if there was no requirement for insulin within the first year of diagnosis, and no prior history of diabetic ketoacidosis. We excluded individuals under the age of 18, individuals with missing data on covariates required for statistical analysis as outlined below, individuals with diabetes other than T2D, and individuals with follow-up times less than 1 month.

The use of anonymised data to study the health and economic impacts of individuals with diabetes has been approved by the Joint-Chinese University of Hong Kong-New Territories East Cluster Clinical Research Ethics Committee. The data used in this study contains anonymised data from Hong Kong's electronic health records. Due to local regulations, these data are unavailable to the public.

### Baseline evaluation and documentation of blood pressure

During RAMP-DM, an individual’s demographics, lifestyle habits, medical history and medication use were collected. Measurements of body weight, body height, waist circumference and BP were performed by trained healthcare professionals. Seated clinic BP was measured using either a manual mercury sphygmomanometer or an automatic sphygmomanometer. Fasting blood samples for plasma glucose, glycated haemoglobin(HbA1c), lipid profile and serum creatinine were taken. Spot urine was collected for albumin-to-creatinine ratio. Hypertension was defined by a SBP ≥ 140 mmHg, a DBP ≥ 90 mmHg, the use of BP-lowering medications, or confirmation by the referring physician.

### Outcome ascertainment

All participants were followed up from the date of RAMP-DM until the occurrence of an outcome, all-cause death or the censored date of 30 June 2024, whichever came first. Clinical outcomes were identified by the International Classification of Diseases(ICD)-9 codes recorded as principal diagnosis in hospital discharge summaries, procedure codes, and laboratory tests (outlined in Table S1**)**. The composite outcome of CVD included stroke, coronary heart disease, and peripheral artery disease. Kidney failure was defined by estimated glomerular filtration rate or by ICD-9 codes for dialysis or kidney transplant. The estimated glomerular filtration rate was estimated using the Chronic Kidney Disease Epidemiology Collaboration equation [16]. Information on death was provided by the HA, which included both in-hospital and out-of-hospital deaths.

### Statistical analysis

Continuous variables were expressed as mean ± standard deviation (SD) if normally distributed or median with interquartile range if skewed. Categorical variables were expressed as percentages with numbers. Baseline between-group comparisons were conducted using Student’s t-test and Mann–Whitney U test for continuous variables, and the Chi-square test for categorical variables.

To explore the age-specific associations between SBP or DBP and clinical outcomes, individuals were categorized by their age at study entry(18–44 years, 45–59 years, 60–74 years, and at or above 75 years). For the primary analysis, multivariate Cox proportional hazard models were used to estimate hazard ratios(HRs) with 95% confidence intervals for the risk of incident CVD, CKD, kidney failure and all-cause death across SBP and DBP categories. The reference group within each age stratum was defined as SBP 120–129 mmHg and DBP 70–79 mmHg– the threshold for defining non-elevated BP by recent guidelines [17]. The primary analysis was conducted for the entire cohort, as well as separately for men and women.

As secondary analyses, we reported the effects of a 10 mmHg and a 1-SD increase in SBP or DBP among individuals with SBP ≥ 120 mmHg and DBP ≥ 70 mmHg on the hazards of incident CVD (and its components), CKD, kidney failure, and all-cause death. We modelled non-linear associations between BP and clinical outcomes using restricted cubic splines with 4 knots positioned at the 5th, 35th, 65th and 95th percentiles, with SBP and DBP expressed as continuous variables. Covariates in the Cox regression models and spline analyses were chosen a priori based on clinical relevance. All analyses were adjusted for year of RAMP-DM assessment, age, sex, duration of diabetes, BMI, smoking status, HbA1c, LDL-cholesterol, HDL-cholesterol, log triglycerides, use of BP-lowering medications, history of CVD, history of CKD, and presence of albuminuria. SBP and DBP were not mutually included as covariates as they are highly correlated. Individuals with baseline CVD, CKD or kidney failure defined based on criteria set for outcomes ascertainment were excluded from the primary and secondary analyses for the corresponding clinical outcomes.

We conducted the following sensitivity analyses. First, we conducted the primary analyses separately for individuals with and without baseline BP-lowering medications. Second, we excluded individuals who developed CVD, CKD, or died within the first year of study entry to minimize possible reverse causality in the associations between BP and clinical outcomes. Third, we adjusted for the competing risk of death using Fine and Gray subdistribution hazard model for the analyes on CVD, CKD and kidney failure. Fourth, we included individuals with missing data by repeating the primary analysis following multiple imputation for missing covariates. We generated 25 imputed datasets, corresponding to the highest proportion of incomplete cases in the dataset, and the results were pooled using Rubin’s rules. Last, we tested whether pulse pressure modified the associations between DBP and outcomes among those with DBP below 70 mmHg by additionally adjusting for pulse pressure in the Cox regression models, as pulse pressure was reported to be a modifier in the risk associations between low DBP and CVD [18].

All statistical tests were two-tailed. A *p*-value of less than 0.05 is considered statistically significant. All statistical analyses were performed using R software (Vienna, Austria: version 4.4.1).

## Results

### Baseline Characteristics

Of 1,049,664 individuals diagnosed with diabetes managed by the Hong Kong HA between 1 Janurary 2000 and 28 February 2022, 63% (N = 661, 523) underwent RAMP-DM. We excluded individuals aged < 18 years (N = 955), individuals with other types of diabetes (N = 4,053), individuals with follow-up time less than one month (N = 133), and individuals with missing data on covariates from our primary analysis (N = 226,642). A total of 429, 740 individuals with T2D and complete data were included in our study (Fig. S1). Compared to individuals without missing data, those with missing data were older, more likely to be female, had a higher mean HbA1c, and had a higher prevalence of hypertension. Standardised mean differences suggested that these differences were small (Table S2).

The baseline characteristics of the study cohort are shown in Table 1. Individuals aged 18–44 years (N = 31,715) at baseline were more likely to be men (56.9% compared to 54.5%, 53.0%, and 45.1% for individuals aged 45–59 years, 60–74 years, and 75 years or older, respectively) and active smokers (23.3% compared to 16.9%, 11.4%, and 5.9% for individuals aged 45–59 years, 60–74 years, and 75 years or older, respectively). Mean BMI, HbA1c, triglyceride, and DBP decreased while SBP increased with increasing age categories. Individuals from the older age categories(45–59, 60–74, and 75 years or above) had a higher prevalence of hypertension(92.2%, 81.4%, 66.5% for individuals aged 75 years or above, 60–74 years and 45–59 years respectively, compared to 50.5% for individuals aged 18–44 years) and higher frequency of use of BP-lowering medications(86.1%, 72.1%, 55.7% for individuals aged 75 years or above, 60–74 years and 45–59 years respectively compared to 37.3% for individuals aged 18–44 years).

**Table 1:** Baseline characteristics of individuals with type 2 diabetes stratified by baseline age at study entry

Characteristics	Total	18–44 years	45–59 years	60–74 years	75 years or older
Number	429,740	31,715	153,918	184,936	59,171
Demographics, family history and lifestyles					
Age (years)	61.90 (11.70)	38.83 (5.53)	53.89 (4.05)	66.62 (4.19)	80.35 (4.15)
Duration of diabetes (years)	1.00 [0.00, 6.00]	1.00 [0.00, 2.00]	1.00 [0.00, 4.00]	2.00 [0.00, 6.00]	3.00 [1.00, 10.00]
Men, n (%)	226578 (52.7)	18039 (56.9)	83852 (54.5)	97986 (53.0)	26701 (45.1)
Family history of diabetes, n (%)	195154 (49.3)	19789 (65.0)	87617 (60.0)	74383 (43.9)	13365 (26.6)
Smoking, n (%)					
Non-smoker	297268 (69.2)	20636 (65.1)	105974 (68.9)	128338 (69.4)	42320 (71.5)
Former smoker	74494 (17.3)	3704 (11.7)	21952 (14.3)	35463 (19.2)	13375 (22.6)
Current smoker	57978 (13.5)	7375 (23.3)	25992 (16.9)	21135 (11.4)	3476 (5.9)
Cardio-metabolic risk factors					
BMI (kg/m^2^)	26.14 (4.32)	28.55 (5.68)	26.55 (4.43)	25.71 (3.92)	25.12 (3.76)
Waist circumference, male (cm)	92.49 (10.31)	96.43 (13.18)	92.61 (10.54)	91.89 (9.57)	91.68 (9.35)
Waist circumference, female (cm)	88.55 (10.48)	90.71 (13.25)	88.18 (10.71)	88.31 (9.96)	89.10 (9.84)
Systolic blood pressure (mmHg)	135.06 (17.92)	129.04 (16.45)	132.00 (17.14)	136.88 (17.75)	140.52 (18.83)
Diastolic blood pressure (mmHg)	76.40 (10.76)	79.44 (11.14)	78.67 (10.43)	75.68 (10.30)	71.09 (10.56)
Hypertension, n (%)	323478 (75.3)	16028 (50.5)	102394 (66.5)	150513 (81.4)	54543 (92.2)
Laboratory investigations					
Fasting plasma glucose (mmol/L)	7.68 (2.48)	8.16 (2.95)	7.91 (2.63)	7.53 (2.31)	7.25 (2.13)
HbA1c (%)	7.39 (1.59)	7.74 (1.95)	7.54 (1.71)	7.29 (1.47)	7.12 (1.31)
HDL cholesterol (mmol/L)	1.26 (0.34)	1.16 (0.30)	1.24 (0.32)	1.28 (0.34)	1.30 (0.36)
LDL cholesterol (mmol/L)	2.71 (0.90)	2.78 (0.88)	2.79 (0.90)	2.67 (0.89)	2.59 (0.88)
Triglycerides (mmol/L)	1.37 [0.99, 1.94]	1.50 [1.04, 2.22]	1.40 [1.00, 2.03]	1.33 [0.97, 1.88]	1.28 [0.95, 1.78]
Estimated GFR (mL/min/1.73m^2^)	82.03 (20.24)	104.66 (16.73)	90.64 (15.93)	77.60 (17.04)	61.61 (17.52)
Urine ACR (mg/mmol)	1.40 [0.68, 4.09]	1.20 [0.60, 3.42]	1.20 [0.60, 3.21]	1.40 [0.70, 3.90]	2.51 [1.03, 8.67]
Medication use, n (%)					
Non-insulin glucose-lowering drugs	300445 (69.9)	22909 (72.2)	110283 (71.7)	127302 (68.8)	39951 (67.5)
Sulphonylurea	137955 (32.1)	9501 (30.0)	48817 (31.7)	57893 (31.3)	21744 (36.7)
Metformin	265445 (61.8)	21155 (66.7)	100304 (65.2)	112251 (60.7)	31735 (53.6)
Thiazolidinediones	4020 (0.9)	551 (1.7)	1598 (1.0)	1550 (0.8)	321 (0.5)
DPP-4 inhibitor	10100 (2.4)	784 (2.5)	3431 (2.2)	4392 (2.4)	1493 (2.5)
SGLT-2 inhibitor	2952 (0.7)	331 (1.0)	1159 (0.8)	1251 (0.7)	211 (0.4)
GLP-1 receptor agonist	101 (0.0)	29 (0.1)	41 (0.0)	27 (0.0)	4 (0.0)
Insulin	23984 (5.6)	2704 (8.5)	8408 (5.5)	9484 (5.1)	3388 (5.7)
Blood pressure-lowering drugs	281985 (65.6)	11829 (37.3)	85781 (55.7)	133406 (72.1)	50969 (86.1)
Alpha blockers	23023 (5.4)	314 (1.0)	3790 (2.5)	12340 (6.7)	6579 (11.1)
Beta blockers	103311 (24.0)	3419 (10.8)	30242 (19.6)	49896 (27.0)	19754 (33.4)
Calcium channel blockers	178286 (41.5)	6625 (20.9)	50808 (33.0)	85498 (46.2)	35355 (59.8)
Centrally acting antihypertensive	8419 (2.0)	168 (0.5)	1304 (0.8)	3983 (2.2)	2964 (5.0)
Nitrates	13610 (3.2)	165 (0.5)	2119 (1.4)	6630 (3.6)	4696 (7.9)
Loop diuretics	11125 (2.6)	324 (1.0)	2298 (1.5)	4807 (2.6)	3696 (6.2)
Potassium sparing diuretics	11312 (2.6)	288 (0.9)	3095 (2.0)	5424 (2.9)	2505 (4.2)
Thiazide	26086 (6.1)	675 (2.1)	6749 (4.4)	12830 (6.9)	5832 (9.9)
Vasodilators	3241 (0.8)	215 (0.7)	696 (0.5)	1407 (0.8)	923 (1.6)
Renin Angiotensin System Inhibitor (RASI)	145203 (33.8)	7332 (23.1)	46838 (30.4)	66627 (36.0)	24406 (41.2)
Angiotensin-Converting Enzyme Inhibitor (ACEI)	107727 (25.1)	5493 (17.3)	35219 (22.9)	48526 (26.2)	18489 (31.2)
Angiotensin II Receptor Blocker (ARB)	38108 (8.9)	1884 (5.9)	11865 (7.7)	18355 (9.9)	6004 (10.1)
Lipid-regulating drugs	177156 (41.2)	7798 (24.6)	56817 (36.9)	85881 (46.4)	26660 (45.1)
Ezetimibe	1670 (0.4)	64 (0.2)	532 (0.3)	909 (0.5)	165 (0.3)
Fibrate	11768 (2.7)	1121 (3.5)	4736 (3.1)	4714 (2.5)	1197 (2.0)
Statin	166587 (38.8)	6784 (21.4)	52627 (34.2)	81626 (44.1)	25550 (43.2)
Antiplatelets	74094 (17.2)	1233 (3.9)	16706 (10.9)	37602 (20.3)	18553 (31.4)
Comorbidities, n (%)					
Urine ACR 3–30 mg/mmol	82319 (24.2)	5477 (21.7)	25922 (21.3)	34532 (23.7)	16388 (34.9)
Urine ACR > 30 mg/mmol	21592 (6.4)	1426 (5.6)	6221 (5.1)	8866 (6.1)	5079 (10.8)
Chronic kidney disease	60393 (14.1)	638 (2.0)	7242 (4.7)	27022 (14.6)	25491 (43.1)
Kidney failure	2642 (0.6)	141 (0.4)	893 (0.6)	1147 (0.6)	461 (0.8)
Cardiovascular Disease	75390 (17.5)	1494 (4.7)	17694 (11.5)	37816 (20.4)	18386 (31.1)
Coronary artery disease	44198 (10.3)	730 (2.3)	10257 (6.7)	22547 (12.2)	10664 (18.0)
Peripheral artery disease	6725 (1.6)	315 (1.0)	1942 (1.3)	2976 (1.6)	1492 (2.5)
Stroke	31723 (7.4)	509 (1.6)	6508 (4.2)	15859 (8.6)	8847 (15.0)
Ischemic stroke	18957 (4.4)	282 (0.9)	4004 (2.6)	9611 (5.2)	5060 (8.6)
Hemorrhagic stroke	4222 (1.0)	123 (0.4)	1225 (0.8)	1932 (1.0)	942 (1.6)
Follow-up time (years)	8.58 [5.08, 12.50]	9.25 [5.58, 13.42]	9.42 [5.75, 13.08]	8.17 [4.92, 12.25]	7.25 [4.25, 10.75]

Data are presented as mean (standard deviation, SD), median [interquartile range, IQR] or number (percentage)

*ACR* albumin-to-creatinine ratio, *DPP*-4 dipeptidyl peptidase-4, *GLP*-1 glucagon-like peptide-1, *SGLT*-2 sodium-glucose co-transporter 2

### Risk associations between SBP, DBP and outcomes across age groups

**Fig. 1:**
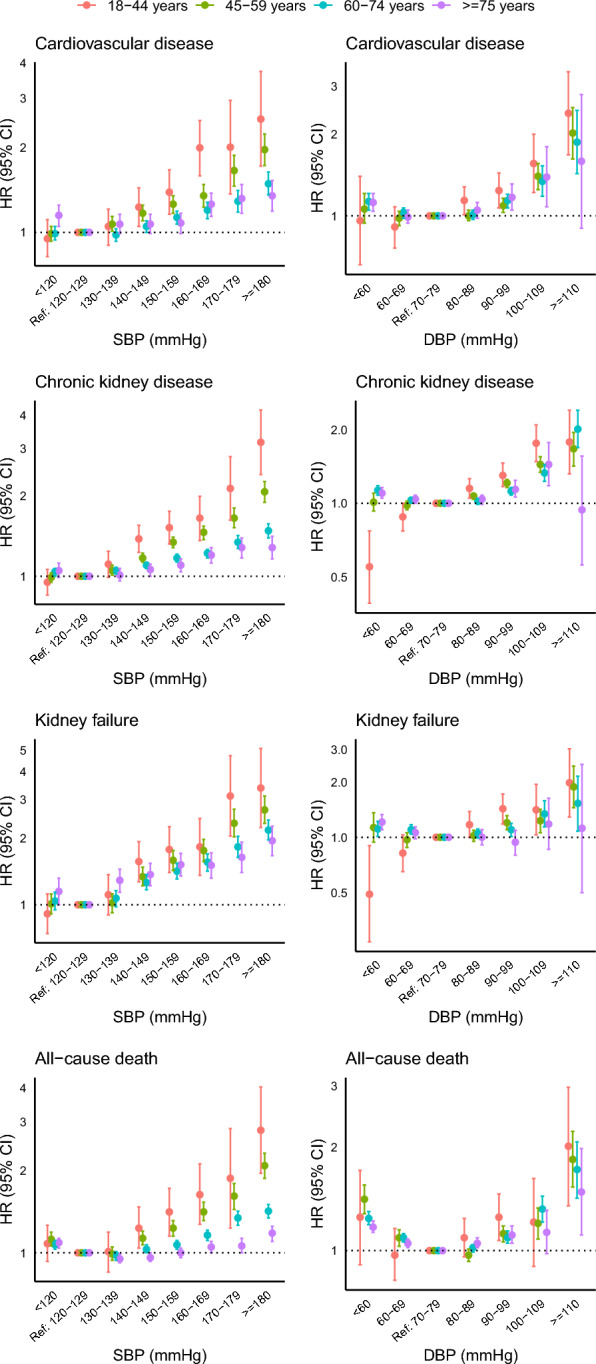
Results of Cox proportional hazard model comparing different systolic blood pressure (SBP) and diastolic blood pressure (DBP) cut-offs referenced to SBP 120–129 mmHg and DBP 70–79 mmHg for incident events stratified by age groups. This is a forest plot showing HRs (95% CI) for cardiovascular disease, chronic kidney disease, end-stage kidney disease and all-cause death for different SBP and DBP categories referenced to SBP 120–129 mmHg and DBP 70–79 mmHg stratified by age categories. The analyses were adjusted for calendar year at assessment, age, sex, diabetes duration, BMI, smoking, HbA1c, LDL cholesterol, HDL cholesterol, log triglycerides, presence of albuminuria, history of CKD, history of CVD and use of blood pressure lowering medications. The corresponding p-values for each between-group comparison are shown in supplementary tables S5 and S6

Over a median follow-up time of 8.6 years (IQR: 5.1, 12.5), we recorded 37,382 incident CVD events among 354,350 individuals(3.09 million person-years follow-up), 96,095 CKD events among 369,347 individuals(2.86 million person-years follow-up), 18,690 kidney failure events among 427,098 individuals(3.79 million person-years follow-up), and 83,826 deaths among 429,740 individuals(3.87 million person-years follow-up). The crude incidence rates of all clinical outcomes increased with rising SBP or DBP across age categories (Tables S3 and S4). With SBP 120–129 mmHg and DBP 70–79 mmHg as reference, increases in SBP and DBP above these ranges were associated with a proportional increase in risks for CVD, CKD, kidney failure and all-cause death across all age groups(Fig. 1**, **Tables S5 and S6). Notably, the dose-response relationships of SBP and DBP with clinical outcomes were steepest in the youngest age category and flattened with increasing age. For all-cause death, a J-shaped association with SBP or DBP was detected in individuals belonging to age categories above 45 years (Fig. 1).

**Fig. 2:**
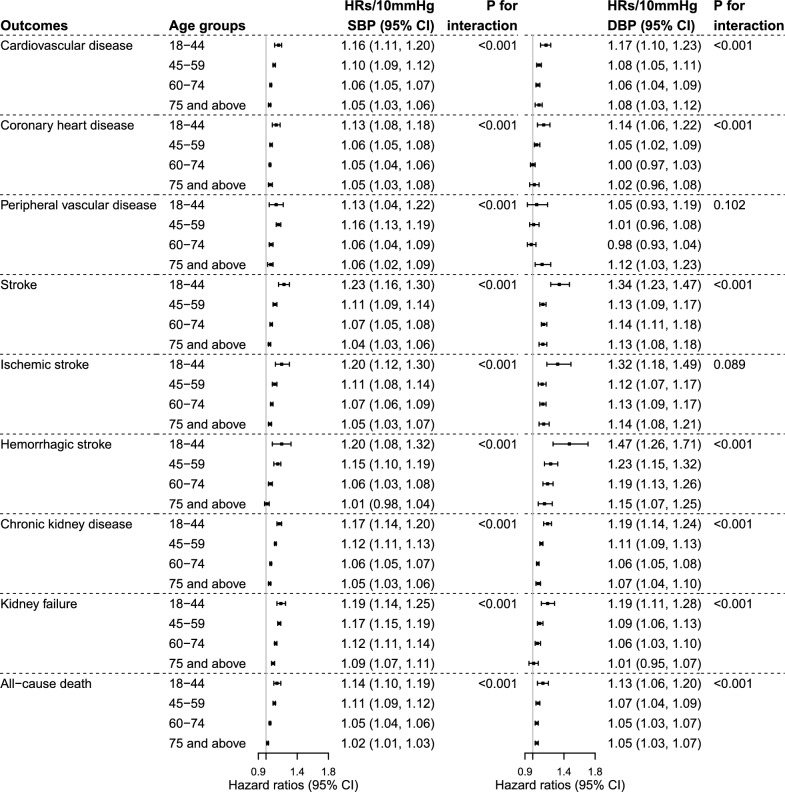
Results of Cox proportional hazard model reporting per 10 mmHg increase in systolic blood pressure (SBP) or diastolic blood pressure (DBP) for incident events stratified by age groups. The analyses were adjusted for calendar year at assessment, age, sex, diabetes duration, BMI, smoking, HbA1c, LDL cholesterol, HDL cholesterol, log triglycerides, presence of albuminuria, history of CKD, history of CVD and use of blood pressure lowering medications. The analyses were limited to individuals with SBP ≥ 120 mmHg and DBP ≥ 70 mmHg

The HRs per 10 mmHg increase in SBP and DBP for most of our study outcomes were greatest in individuals aged 18–44 years and declined with increasing age (Fig. 2**, **Table S7). This pattern was consistent when the analysis was performed using a 1-SD increment increase in SBP or DBP (Table S8). Each 10 mmHg increase in SBP was associated with 16%(HR 1.16 [95% CI: 1.11, 1.20]), 17%(HR 1.17 [1.14, 1.20]), 19%(HR 1.19 [1.14, 1.25]), and 14%(HR 1.14 [1.10, 1.19]) relative increase in risks of incident CVD, CKD, kidney failure and all-cause death respectively for individuals aged 18–44 years. Among individuals age 75 years or above, the corresponding increase in risks associated with each 10 mmHg increase in SBP were 5%(HR 1.05 [1.03, 1.06]), 5%(HR 1.05 [1.03, 1.06]), 9%(HR 1.09 [1.07, 1.11]), 2%(HR 1.02 [1.01, 1.03]) for these outcomes. The risk associations of DBP with study outcomes similarly showed attenuation in intensity with increasing age. Among individual components of CVD, the effect per 10 mmHg or 1-SD increase in DBP was the greatest for hemorrhagic stroke, conferring higher risks among those in the youngest age category 18–44 years compared to other age groups (Tables S7 and S8). Sex stratified analyses demonstrated consistent patterns of declining risk association between SBP, DBP and our study outcomes with increasing age(Tables S9–S12).

### Non-linear associations between SBP, DBP and outcomes

**Fig. 3:**
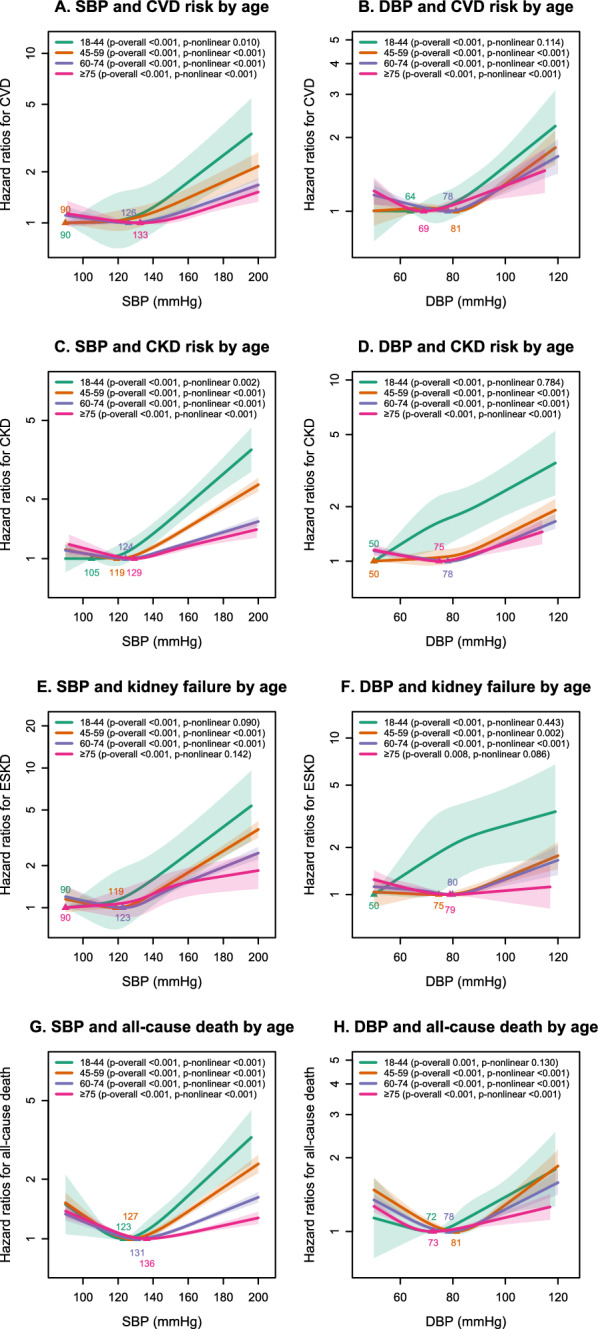
The association of systolic blood pressure (SBP) and diastolic blood pressure (DBP) with the development of incident events by restricted cubic spline stratified by age groups. SBP and DBP were modelled as continuous variables allowing for non-linear effects with 4 knots positioned at quantiles (0.05, 0.35, 0.65, 0.95). The shaded areas represent the 95% C.I. The analyses were adjusted for calendar year at assessment, age, sex, diabetes duration, BMI, smoking, HbA1c, LDL cholesterol, HDL cholesterol, log triglycerides, presence of albuminuria, history of CKD, history of CVD and use of blood pressure lowering medications

Restricted cubic spline demonstrated variable patterns of association between SBP or DBP and clinical outcomes, with different nadir SBP or DBP associated with the least hazard for incident clinical outcomes across age categories (Fig. 3). The relationship between SBP and CVD, CKD, and all-cause death was non-linear in all age categories (*p*-value for non-linearity < 0.05 for all). The relationship between DBP and clinical outcomes was non-linear for most age groups, except for the individuals aged 18–44 years, in which the test for non-linearity in the association between DBP and CVD, CKD, kidney failure and all-cause death was insignificant, indicating a predominantly linear association (Fig. 3). Spline analysis indicated a significant nonlinear association between SBP/DBP with all-cause death for individuals aged 45 years or older (*p*-value for nonlinearity < 0.001), and the corresponding curves suggested a J-shaped association.

### Sensitivity analyses

The observed declining intensity of risk associations between SBP, DBP and study outcomes with increasing age remained largely consistent in the sensitivity analyses after adjustment for the competing risk of death (Tables S17, S18) and stratification based on baseline BP-lowering medication use (Tables S13–S16). Irrespective of baseline BP-lowering medications, risk associations between high SBP and DBP and our study outcomes remained greatest for individuals aged 18–44 years (Tables S13–S16). Excluding individuals who developed CVD, CKD, and death within the first year of study entry attenuated the J-shaped association between SBP and all-cause death, but the observed higher risk of death associated with DBP below 70–79 mmHg for individuals aged 45 years or above remained significant (Tables S19 and S20 and Fig. S2). Multiple imputation analyses were consistent with the complete case analyses (Tables S21 and S22). Lastly, risk associations with DBP were not modified when adjusting for pulse pressure among individuals with DBP below 70 mmHg (Table S23).

## Discussion

In this population-based longitudinal study of individuals with T2D, we examined the age-specific associations between BP and diabetes-related outcomes. We report the following key findings: First, high SBP and DBP remain a significant modifiable cardiovascular risk factor, conferring excess risks for CVD, kidney disease and all-cause death in individuals with T2D, irrespective of age. Second, heterogeneity by age was observed in the strength of these risk associations, with an overall greater effect observed among individuals aged 18 to 44 years in our cohort. Third, we observed a J-shaped association with increased risk of death in individuals aged 45 years or older with DBP below 70–79 mmHg, but caution should be exercised in interpreting this finding from a retrospective study.

In our study, the risk associations between high SBP, DBP and cardiovascular outcomes decline with age, consistent with other large-scale population-based analyses from a general [8–10] and diabetes population [11]. The Asia Pacific Cohort Studies Collaboaration cohort which included 23, 651participants with diabetes showed that SBP was positively associated with incident coronary heart disease, hemorrhagic stroke and ischemic stroke in those aged below 65 but not in older age group, although the statistical interaction between SBP and age was not significant [19]. Another population-based study comprising 180, 492 individuals with diabetes in Hong Kong reported 33% and 9% increase in adjusted hazards for incident CVD per 10 mmHg increase in SBP in individuals aged below 50 and those aged 70–79 respectively [11]. Our study extends these observations by elucidating the age-specific association of both SBP and DBP with other important clinical outcomes, including CKD, kidney failure and all-cause mortality in individuals with T2D. Despite higher crude incidence rates for these events in the older populations, the slope of the increase in HRs for our study outcomes per increase in SBP or DBP was the steepest in individuals from the 18–44 year old category. We further demonstrated that nonlinear associations exist, with variable nadirs of BP corresponding to the lowest risks for clinical outcomes by age. These observed age-specific heterogeneities may reflect different aetiologies of hypertension. Young individuals with high BP may have underlying secondary endocrine causes [20], renovascular disease [20], obstructive sleep apnea, or familial factors [21], which can contribute to a higher risk for CVD and other outcomes independent of BP. Likewise, older individuals with age-related hypertension may have other contributing factors to CVD and kidney disease related to ageing apart from raised BP alone.

We observed that the risks for cardiovascular disease, kidney outcomes and death remained greatest among young individuals irrespective of baseline BP-lowering medications use. However, we lacked information on the intensification of the BP-lowering medications regimen and did not account for the time-varying nature of the regimens, which may influence risk associations. Moreover, subgrouping by BP-lowering medication use further stratifies the study population, thereby reducing statistical power and may underestimate true risk associations. This is particularly relevant for individuals in the older age categories, where baseline BP-lowering medication use is more common. In this regard, previous studies have reported that residual cardiovascular risks associated with hypertension remain despite BP-lowering medications [22, 23], highlighting the importance of hypertension prevention.

Among the specific components of CVD, the risks conferred by an incremental increase in SBP or DBP were particularly high for hemorrhagic stroke. Each 10 mmHg or 1-SD increase in DBP conferred a 47 to 51% increase in relative risk for hemorrhagic stroke, while the corresponding risk conferred by SBP was 20 to 38% for individuals aged 18–44 years. Physiologically, SBP represents the peak arterial pressure during ventricular contraction, while DBP reflects BP during diastole, thus more closely reflects peripheral vascular resistance [24, 25]. These differences may contribute to differing risks in vascular rupture as reported in studies on recurrent intracranial haemorrhage [26]. Concurrently, the predominantly linear associations between DBP and CVD, kidney failure and death for individuals aged 18–44 years further highlighted the importance of controlling high DBP in this population. A previous population-based study conducted in Korea showed that among 6,424,090 young adults aged 20–39 years, isolated diastolic hypertension is the most prevalent form of hypertension and confers comparable cardiovascular risk as isolated systolic hypertension [27]. Given the strong correlation between SBP and DBP, future studies should explore the combined and separate effects of these BP components in age-stratified cohorts to verify our findings.

In our study, there was a J-shaped association between BP and death, which was reported in observational studies [28–31] and post-hoc analyses of interventional trials [32]. Nonetheless, potential bias from reverse causality, such as pre-existing illness leading to low BP before death [33] would limit the interpretations from observational studies. Recent population-based cohort studies in the diabetes population have not reported increased CVD or death with SBP down to 120 mmHg [11, 19], though associations with DBP were not studied. The exclusion of individuals who developed CVD, CKD and death within 1 year of study entry in our study attenuated the association for low SBP, suggesting possible reverse causality. Nonetheless, the increased risk of death remained significant for low DBP, particularly for older adults aged 45 or above.

The adverse cardiovascular effects of low DBP may be driven by a widened pulse pressure, a marker of arterial stiffness [18], although our analysis found no evidence of effect modification by pulse pressure. Other possible reasons for the J-curve association include impaired coronary perfusion, which primarily occurs during diastole [34]. This is supported by studies that have demonstrated increased hazards for cardiovascular death associated with low DBP in individuals with baseline coronary heart disease [30] or subclinical atherosclerosis [31]. Other unmeasured confounders, including differences in treatment intensity, may also be at play. For instance, individuals with low DBP may receive fewer prescriptions of BP-lowering medications despite having a high SBP. In this regard, cardiac autonomic neuropathy is associated with a longer duration of diabetes and older age [35] and is an independent risk factor for mortality in individuals with type 2 diabetes [36, 37]. Its manifestations, including BP fluctuations and orthostatic hypotension, may affect tolerance to BP-lowering treatment, possibly confounding our results. Low DBP may also be a marker of frailty [38] and subclinical myocardial damage [39], which confounds the observed higher risk for death.

Recent guidelines have supported lowering the hypertension treatment threshold to SBP below 130 mmHg with encouragement to achieve below 120 mmHg for individuals with diabetes [40]. While we observed that the risks associated with elevated BP attenuated with increasing age, our results may be influenced by survival bias from the highly selected survivors in an observational cohort, thereby masking the true effects of elevated BP. The BPROAD study enrolled 12,821 middle-aged individuals with T2D with baseline cardiovascular risk, showed that intensive BP target with SBP below 120 mmHg confers meaningful cardiovascular risk reduction, albeit a neutral effect on CKD, compared to the conventional target of SBP below 140 mmHg [41]. Similarly, meta-analysis of randomised controlled trials reported that treatment targets with SBP below 130 mmHg or 120 mmHg significantly reduce mortality, with few adverse effects [42]. Yet young adults have historically been underrepresented in interventional trials related to hypertension management. Using a large sample size and a structured diabetes assessment database with great data granularity, we demonstrated significant age-specific heterogeneities in the risk associations between elevated BP and cardiovascular, kidney outcomes and death. Although our study design does not allow us to conclude the optimal BP target, the observed greater intensities of risk associations among young individuals would call for increased awareness and early intervention for high BP in this population.

Our study is not without limitations. First, we analysed SBP and DBP as separate variables, and we did not examine the joint effects of SBP and DBP on the risk associations with our clinical outcomes. Second, although we have adjusted for age in our model, we did not adjust for the time-varying nature of BP and other cardiovascular risk factors that may occur with ageing. Third, we analysed baseline office BP as the exposure and did not adjust for regression dilution, as in some [9–11] but not all studies [8] that have explored the effect of BP and cardiovascular outcomes. Owing to the retrospective nature of our study, repeated BP readings within the individuals in our cohort may be influenced by treatment and disease progression during follow-up. Applying regression dilution correction may introduce additional bias. However, in the absence of such adjustment, we acknowledge that the risk associations between SBP, DBP, and our outcomes may be underestimated or subject to measurement errors. The use of office BP as our exposure may also include individuals with white-coat hypertension in our risk estimations. Therefore, as a sensitivity analyses we used the prescription of BP-lowering medications to demonstrate risk associations among individuals with treated hypertension. Fourth, our study cohort included individuals with T2D who enrolled in RAMP-DM. Participation in RAMP-DM was associated with a reduced incidence of diabetes-related complications and hospitalisation compared with non-participation [13]. As a result, our study is subject to selection bias by including a diabetes population that has a reduced incidence of cardiovascular and kidney outcomes. Fifth, we studied the outcome of all-cause death, which is an unbiased endpoint, but it lacks specificity with respect to its mechanism. Lastly, our study population comprises mainly people of Chinese ethnicity. Therefore, our findings may not apply to other populations.

## Conclusions

To conclude, we demonstrated age-specific heterogeneities in the association between BP, cardiovascular and kidney outcomes and all-cause death among individuals with T2D. The impact of high SBP or DBP and the associated risks for incident CVD, kidney outcomes and death were greatest among young individuals aged 18 to 44 years compared to other ages. Efforts are needed to promote blood pressure screening and treatment among young individuals with T2D. The scarcity of clinical trial data pertaining to young individuals with hypertension represents a significant research gap that must be addressed to inform effective treatment strategies and goals.

## Supplementary Information

Supplementary Material 1

## Data Availability

The data underlying the resultspresented in the study are hosted by the Hong Kong Hospital Authority. Due to local regulation, the data are not available to the public. Request for data can be made via Hong Kong HospitalAuthority.

## Abbreviations

BP: Blood pressure
BMI: Body mass index
CVD: Cardiovascular disease
CKD: Chronic kidney disease
DBP: Diastolic blood pressure
HbA1c: Glycated haemoglobin
HR: Hazard ratio
SBP: Systolic blood pressure
T2D: Type 2 diabetes
