# Supplementary material for: Age-specific associations between blood pressure and cardiovascular disease, kidney disease, and death among individuals with type 2 diabetes: a population-based cohort study
Source: Cardiovasc Diabetol. 2026 Jan 28;25:60. doi: 10.1186/s12933-025-03072-1 (PMC12922390; doi:10.1186/s12933-025-03072-1)

**Title**: Age-specific associations between blood pressure and cardiovascular disease, kidney disease, and death among people with type 2 diabetes: a population-based cohort study.

**Supplementary Table S1**: ICD-9 Codes used for outcomes ascertainment.

**Supplementary Table S2:** Baseline characteristics of individuals with type 2 diabetes, stratified by presence or absence of missing data on baseline blood pressure and confounding variables.

**Supplementary Table S3**: Crude incidence (per 1,000 person-year) of cardiovascular disease, kidney disease and all-cause death in individuals with type 2 diabetes, stratified by systolic blood pressure (SBP) and age.

**Supplementary Table S4:** Crude incidence (per 1,000 person-year) of cardiovascular disease, kidney disease and all-cause death in individuals with type 2 diabetes, stratified by diastolic blood pressure (DBP) and age.

**Supplementary Table S5:** Cox regression models for the associations of different systolic blood pressure (SBP) cut-offs (referenced to SBP 120 – 129 mmHg) with incident events among adults with type 2 diabetes, stratified by age groups.

**Supplementary Table S6:** Cox regression models for the associations of different diastolic blood pressure (DBP) cut-offs (referenced to DBP 70 – 79 mmHg) with incident events, stratified by age groups.

**Supplementary Table S7:** Cox regression models for the effect of each 10 mmHg increase in systolic blood pressure (SBP) and diastolic blood pressure (DBP) on incident events, stratified by age groups.

**Supplementary Table S8:** Cox regression models for the effect of each 1 standard deviation (SD) increase in systolic blood pressure (SBP) and diastolic blood pressure (DBP) on incident events, stratified by age groups.

**Supplementary Table S9:** Cox regression models for the associations of different systolic blood pressure (SBP) cut-offs (referenced to SBP 120 – 129 mmHg) with incident events among females (n = 203,162), stratified by age groups.

**Supplementary Table S10:** Cox regression models for the associations of different diastolic blood pressure (DBP) cut-offs (referenced to DBP 70 – 79 mmHg) with incident events among females (n = 203,162), stratified by age groups.

**Supplementary Table S11:** Cox regression models for the associations of different systolic blood pressure (SBP) cut-offs (referenced to SBP 120 – 129 mmHg) with incident events among males (n = 226,578), stratified by age groups.

**Supplementary Table S12:** Cox regression models for the associations of different diastolic blood pressure (DBP) cut-offs (referenced to DBP 70 – 79 mmHg) with incident events among males (n = 226,578), stratified by age groups.

**Supplementary Table S13**: Cox regression models for the associations of different systolic blood pressure (SBP) cut-offs (referenced to SBP 120 – 129 mmHg) with incident events among individuals who were using blood pressure lowering medications at baseline (n = 281,985), stratified by age groups.

**Supplementary Table S14**: Cox regression models for the associations of different diastolic blood pressure (DBP) cut-offs (referenced to DBP 70 – 79 mmHg) with incident events among individuals who were using blood pressure lowering medications at baseline (n = 281,985), stratified by age groups.

**Supplementary Table S15**: Cox regression models for the associations of different systolic blood pressure (SBP) cut-offs (referenced to SBP 120 – 129 mmHg) with incident events among individuals who were **not** using blood pressure lowering medications at baseline (n = 147,755), stratified by age groups.

**Supplementary Table S16**: Cox regression models for the associations of different diastolic blood pressure (DBP) cut-offs (referenced to DBP 70 – 79 mmHg) with incident events among individuals who were **not** using blood pressure lowering medications at baseline (n = 147,755), stratified by age groups.

**Supplementary Table S17:** Cox regression models for the associations of different systolic blood pressure (SBP) cut-offs (referenced to SBP 120 – 129 mmHg) with incident events stratified by age groups, accounting for competing risk of death.

**Supplementary Table S18:** Cox regression models for the associations of different diastolic blood pressure (DBP) cut-offs (referenced to DBP 70 – 79 mmHg) with incident events stratified by age groups, accounting for competing risk of death.

**Supplementary Table S19**: Cox regression models for the associations of different systolic blood pressure (SBP) cut-offs (referenced to SBP 120 – 129 mmHg) with incident events, stratified by age groups. This analysis excluded individuals who had history of cardiovascular disease or chronic kidney disease, and those who developed cardiovascular disease, chronic kidney disease or all-cause death within 1 year of study entry (n = 303,949).

**Supplementary Table S20**: Cox regression models for the associations of different diastolic blood pressure (DBP) cut-offs (referenced to DBP 70 – 79 mmHg) with incident events, stratified by age groups. This analysis excluded individuals who had history of cardiovascular disease or chronic kidney disease, and those who developed cardiovascular disease, chronic kidney disease or all-cause death within 1 year of study entry (n = 303,949).

**Supplementary Table S21**: Cox regression models for the associations of different systolic blood pressure (SBP) cut-offs (referenced to SBP 120 – 129 mmHg) with incident events, stratified by age groups, using multiple imputation approach (n = 573,780).

**Supplementary Table S22**: Cox regression models for the associations of different diastolic blood pressure (DBP) cut-offs (referenced to DBP 70 – 79 mmHg) with incident events, stratified by age groups, using multiple imputation approach (n = 573,718).

**Supplementary Table S23:** Cox regression for the association of 10 mmHg increase in diastolic blood pressure (DBP) and pulse pressure (PP) with incident events among individuals with DBP < 70 mmHg (n = 110,950).

**Supplementary Figure S1**: Study flow chart

**Supplementary Figure S2:** Results of Cox proportional hazard model comparing different systolic blood pressure (SBP) and diastolic blood pressure (DBP) cut-offs referenced to SBP 120 – 129 mmHg and DBP 70 – 79 mmHg for incident events stratified by age categories. This analysis excluded individuals who had history of cardiovascular disease or chronic kidney disease, and those who developed cardiovascular disease, chronic kidney disease or all-cause death within 1 year of study entry.

**Supplementary Table S1**: ICD-9 Codes used for outcomes ascertainment

| Outcome | Laboratory test | ICD-9 diagnostic codes | ICD-9 Procedure codes |
| --- | --- | --- | --- |
| Coronary heart disease |  | 410-414 |  |
| Ischemic stroke |  | 433 – 434 |  |
| Haemorrhagic stroke |  | 430 – 432 |  |
| Stroke (not specified) |  | 436-438 |  |
| Peripheral artery disease |  | 250.7, 785.4, 443.81, 443.9 | Surgical revascularization: 38.08, 38.18, 38.38, 38.48, 38.68, 38.88, 39.25, 39.29, 39.49, 39.56, 39.57, 39.58, 39.59, 39.99  Endovascular revascularization: 00.55, 17.56, 39.50, 39.79, 39.90  Lower extremity amputation: 84.10- 84.19  Excluding diagnosis of traumatic amputation [ICD-9 codes: 895-897] |
| Chronic kidney disease | At least two estimated glomerular filtration rate (eGFR) <60 mL/min/1.73m^2^ with an interval time longer than three months within one year | Kidney transplant status: V42.0  Complications of transplanted kidney: 996.81 | Dialysis (54.98 or 39.95 with a principal or secondary diagnosis code of chronic kidney disease: 585 or kidney failure: 586, excluding those with diagnosis code of acute kidney injury: 584).  Kidney transplant: 55.6 |
| Kidney failure | At least two estimated GFR <15 ml/min/1.73m^2^ with an interval time longer than three months within one year |  |  |

**Supplementary Table S2:** Baseline characteristics of individuals with type 2 diabetes, stratified by presence or absence of missing data on baseline blood pressure and confounding variables.

| **Characteristics** | **With missing data** | | **Without missing data** | | ***p* value** | **SMD** |
| --- | --- | --- | --- | --- | --- | --- |
|  | **Numbers** | **Values** | **Numbers** | **Values** |  |  |
| **Demographics, family history and lifestyles** |  |  |  |  |  |  |
| Age (years) | 226642 | 63.64 (12.12) | 429740 | 61.90 (11.70) | <0.001 | 0.146 |
| Duration of diabetes (years) | 186775 | 2.00 [1.00, 7.00] | 429740 | 1.00 [0.00, 6.00] | <0.001 | 0.134 |
| Men, n (%) | 226642 | 108518 (47.9) | 429740 | 226578 (52.7) | <0.001 | 0.097 |
| Family history of diabetes, n (%) | 124147 | 52945 (42.6) | 396193 | 195154 (49.3) | <0.001 | 0.133 |
| Smoking, n (%) | 226642 |  | 429740 |  | <0.001 | 0.276 |
| Non-smoker |  | 183587 (81.0) |  | 297268 (69.2) |  |  |
| Former smoker |  | 24476 (10.8) |  | 74494 (17.3) |  |  |
| Current smoker |  | 18579 (8.2) |  | 57978 (13.5) |  |  |
| **Cardio-metabolic risk factors** |  |  |  |  |  |  |
| BMI (kg/m^2^) | 135112 | 25.92 (4.13) | 429740 | 26.14 (4.32) | <0.001 | 0.051 |
| Waist circumference, male (cm) | 58852 | 91.74 (9.98) | 221104 | 92.49 (10.31) | <0.001 | 0.075 |
| Waist circumference, female (cm) | 60911 | 87.86 (10.23) | 197690 | 88.55 (10.48) | <0.001 | 0.067 |
| Systolic blood pressure (mmHg) | 144040 | 136.80 (18.45) | 429740 | 135.06 (17.92) | <0.001 | 0.096 |
| Diastolic blood pressure (mmHg) | 143978 | 75.71 (10.51) | 429740 | 76.40 (10.76) | <0.001 | 0.064 |
| Hypertension, n (%) | 198830 | 168151 (84.6) | 429740 | 323478 (75.3) | <0.001 | 0.234 |
| **Laboratory investigations** |  |  |  |  |  |  |
| Fasting plasma glucose (mmol/L) | 79672 | 7.61 (2.73) | 371080 | 7.68 (2.48) | <0.001 | 0.028 |
| HbA1c (%) | 121675 | 7.46 (1.68) | 429740 | 7.39 (1.59) | <0.001 | 0.041 |
| HDL cholesterol (mmol/L) | 67020 | 1.25 (0.35) | 429740 | 1.26 (0.34) | <0.001 | 0.022 |
| LDL cholesterol (mmol/L) | 60693 | 2.90 (0.93) | 429740 | 2.71 (0.90) | <0.001 | 0.207 |
| Triglycerides (mmol/L) | 78474 | 1.43 [1.00, 2.20] | 429740 | 1.37 [0.99, 1.94] | <0.001 | 0.253 |
| Estimated GFR (mL/min/1.73m^2^) | 113523 | 77.81 (21.96) | 418727 | 82.03 (20.24) | <0.001 | 0.199 |
| Urine ACR (mg/mmol) | 50851 | 1.55 [0.72, 4.63] | 339584 | 1.40 [0.68, 4.09] | <0.001 | 0.016 |
| **Medication use, n (%)** |  |  |  |  |  |  |
| Non-insulin glucose-lowering drugs | 226642 | 133467 (58.9) | 429740 | 300445 (69.9) | <0.001 | 0.232 |
| Sulphonylurea | 226642 | 81513 (36.0) | 429740 | 137955 (32.1) | <0.001 | 0.082 |
| Metformin | 226642 | 106808 (47.1) | 429740 | 265445 (61.8) | <0.001 | 0.297 |
| Thiazolidinediones | 226642 | 667 (0.3) | 429740 | 4020 (0.9) | <0.001 | 0.082 |
| DPP-4 inhibitor | 226642 | 1239 (0.5) | 429740 | 10100 (2.4) | <0.001 | 0.151 |
| SGLT-2 inhibitor | 226642 | 261 (0.1) | 429740 | 2952 (0.7) | <0.001 | 0.091 |
| GLP-1 receptor agonist | 226642 | 10 (0.0) | 429740 | 101 (0.0) | <0.001 | 0.016 |
| Insulin | 226642 | 6970 (3.1) | 429740 | 23984 (5.6) | <0.001 | 0.123 |
| Blood pressure lowering medications | 226642 | 152235 (67.2) | 429740 | 281985 (65.6) | <0.001 | 0.033 |
| Alpha blockers | 226642 | 12417 (5.5) | 429740 | 23023 (5.4) | 0.039 | 0.005 |
| Beta blockers | 226642 | 57742 (25.5) | 429740 | 103311 (24.0) | <0.001 | 0.033 |
| Calcium channel blockers | 226642 | 92772 (40.9) | 429740 | 178286 (41.5) | <0.001 | 0.011 |
| Centrally acting antihypertensive | 226642 | 9336 (4.1) | 429740 | 8419 (2.0) | <0.001 | 0.126 |
| Nitrates | 226642 | 5958 (2.6) | 429740 | 13610 (3.2) | <0.001 | 0.032 |
| Loop diuretics | 226642 | 4224 (1.9) | 429740 | 11125 (2.6) | <0.001 | 0.049 |
| Potassium sparing diuretics | 226642 | 8778 (3.9) | 429740 | 11312 (2.6) | <0.001 | 0.070 |
| Thiazide | 226642 | 21347 (9.4) | 429740 | 26086 (6.1) | <0.001 | 0.126 |
| Vasodilators | 226642 | 1319 (0.6) | 429740 | 3241 (0.8) | <0.001 | 0.021 |
| Renin Angiotensin System Inhibitor (RASI) | 226642 | 63994 (28.2) | 429740 | 145203 (33.8) | <0.001 | 0.120 |
| Angiotensin-Converting Enzyme Inhibitor (ACEI) | 226642 | 54598 (24.1) | 429740 | 107727 (25.1) | <0.001 | 0.023 |
| Angiotensin II Receptor Blocker (ARB) | 226642 | 9540 (4.2) | 429740 | 38108 (8.9) | <0.001 | 0.189 |
| Lipid-regulating drugs | 226642 | 52076 (23.0) | 429740 | 177156 (41.2) | <0.001 | 0.399 |
| Ezetimibe | 226642 | 141 (0.1) | 429740 | 1670 (0.4) | <0.001 | 0.069 |
| Fibrate | 226642 | 8051 (3.6) | 429740 | 11768 (2.7) | <0.001 | 0.047 |
| Statin | 226642 | 44257 (19.5) | 429740 | 166587 (38.8) | <0.001 | 0.433 |
| Antiplatelets | 226642 | 27273 (12.0) | 429740 | 74094 (17.2) | <0.001 | 0.148 |
| **Comorbidities, n (%)** |  |  |  |  |  |  |
| Urine ACR 3 – 30 mg/mmol | 50851 | 13374 (26.3) | 339584 | 82319 (24.2) | <0.001 | 0.047 |
| Urine ACR > 30 mg/mmol | 50851 | 3472 (6.8) | 339584 | 21592 (6.4) | <0.001 | 0.019 |
| Chronic kidney disease | 226642 | 23389 (10.3) | 429740 | 60393 (14.1) | <0.001 | 0.114 |
| Kidney failure | 226642 | 819 (0.4) | 429740 | 2642 (0.6) | <0.001 | 0.036 |
| Cardiovascular disease | 226642 | 25980 (11.5) | 429740 | 75390 (17.5) | <0.001 | 0.173 |
| Coronary artery disease | 226642 | 12218 (5.4) | 429740 | 44198 (10.3) | <0.001 | 0.183 |
| Peripheral artery disease | 226642 | 3159 (1.4) | 429740 | 6725 (1.6) | <0.001 | 0.014 |
| Stroke | 226642 | 13172 (5.8) | 429740 | 31723 (7.4) | <0.001 | 0.063 |
| Ischemic stroke | 226642 | 6134 (2.7) | 429740 | 18957 (4.4) | <0.001 | 0.092 |
| Hemorrhagic stroke | 226642 | 1744 (0.8) | 429740 | 4222 (1.0) | <0.001 | 0.023 |
| **Follow-up time (years)** | 226642 | 12.33 [6.75, 15.75] | 429740 | 8.58 [5.08, 12.50] | <0.001 | 0.480 |

Data are presented as mean (standard deviation), median [interquartile range] or number (percentage). SMD, standardized mean difference. An SMD < 0.2 was regarded as indicating an acceptable balance between groups.

ACR: albumin-to-creatinine ratio; DPP-4: dipeptidyl peptidase-4; GLP-1: glucagon-like peptide-1; SGLT-2: sodium-glucose co-transporter 2

Baseline confounding variables included calendar year at assessment, age, sex, diabetes duration, body mass index, smoking status, HbA1c, HDL cholesterol, LDL cholesterol, triglycerides, presence of albuminuria, history of chronic kidney disease, history of cardiovascular disease, and use of blood pressure lowering medications.

**Supplementary Table S3**: Crude incidence (per 1,000 person-year) of cardiovascular disease, kidney disease and all-cause death in individuals with type 2 diabetes, stratified by systolic blood pressure (SBP) and age.

| **Outcome** | **Age categories** | **SBP categories (mmHg)** | **Number of people at risk** | **Events** | **Total person-year** | **Crude incidence per 1,000 person-year (95% CI)** |
| --- | --- | --- | --- | --- | --- | --- |
| Cardiovascular disease | All | All | 354350 | 37382 | 3092079 | 12.09 (11.97, 12.21) |
|  | 18-44 years | <120 | 8814 | 359 | 88400 | 4.06 (3.65, 4.50) |
|  |  | 120-129 | 7472 | 345 | 70800 | 4.87 (4.37, 5.42) |
|  |  | 130-139 | 6533 | 360 | 61652 | 5.84 (5.25, 6.47) |
|  |  | 140-149 | 4301 | 307 | 40973 | 7.49 (6.68, 8.38) |
|  |  | 150-159 | 1966 | 181 | 19185 | 9.43 (8.11, 10.91) |
|  |  | 160-169 | 741 | 102 | 7092 | 14.38 (11.73, 17.46) |
|  |  | 170-179 | 227 | 29 | 2117 | 13.70 (9.17, 19.67) |
|  |  | ≥180 | 167 | 28 | 1360 | 20.58 (13.68, 29.75) |
|  | 45-59 years | <120 | 31894 | 2042 | 299457 | 6.82 (6.53, 7.12) |
|  |  | 120-129 | 32280 | 2118 | 292240 | 7.25 (6.94, 7.56) |
|  |  | 130-139 | 31034 | 2446 | 289533 | 8.45 (8.12, 8.79) |
|  |  | 140-149 | 21769 | 2029 | 207230 | 9.79 (9.37, 10.23) |
|  |  | 150-159 | 11567 | 1311 | 114709 | 11.43 (10.82, 12.06) |
|  |  | 160-169 | 4677 | 609 | 46918 | 12.98 (11.97, 14.05) |
|  |  | 170-179 | 1765 | 288 | 17279 | 16.67 (14.80, 18.71) |
|  |  | ≥180 | 1238 | 262 | 11919 | 21.98 (19.40, 24.81) |
|  | 60-74 years | <120 | 22441 | 2153 | 181520 | 11.86 (11.37, 12.37) |
|  |  | 120-129 | 29874 | 2809 | 237638 | 11.82 (11.39, 12.27) |
|  |  | 130-139 | 34699 | 3508 | 283706 | 12.36 (11.96, 12.78) |
|  |  | 140-149 | 28484 | 3402 | 241923 | 14.06 (13.59, 14.54) |
|  |  | 150-159 | 17696 | 2553 | 154672 | 16.51 (15.87, 17.16) |
|  |  | 160-169 | 8246 | 1353 | 72374 | 18.69 (17.71, 19.72) |
|  |  | 170-179 | 3276 | 589 | 28429 | 20.72 (19.08, 22.46) |
|  |  | ≥180 | 2404 | 562 | 21070 | 26.67 (24.51, 28.97) |
|  | ≥75 years | <120 | 4723 | 861 | 33537 | 25.67 (23.99, 27.45) |
|  |  | 120-129 | 6694 | 1056 | 48547 | 21.75 (20.46, 23.10) |
|  |  | 130-139 | 8978 | 1554 | 65544 | 23.71 (22.55, 24.92) |
|  |  | 140-149 | 8566 | 1553 | 63577 | 24.43 (23.23, 25.67) |
|  |  | 150-159 | 6122 | 1203 | 46432 | 25.91 (24.47, 27.42) |
|  |  | 160-169 | 3074 | 710 | 22756 | 31.20 (28.95, 33.58) |
|  |  | 170-179 | 1464 | 376 | 10891 | 34.52 (31.12, 38.20) |
|  |  | ≥180 | 1164 | 324 | 8598 | 37.68 (33.69, 42.02) |
| Chronic kidney disease | All | All | 369347 | 96095 | 2858138 | 33.62 (33.41, 33.83) |
|  | 18-44 years | <120 | 9020 | 633 | 88999 | 7.11 (6.57, 7.69) |
|  |  | 120-129 | 7692 | 597 | 71705 | 8.33 (7.67, 9.02) |
|  |  | 130-139 | 6751 | 635 | 62160 | 10.22 (9.44, 11.04) |
|  |  | 140-149 | 4424 | 561 | 40422 | 13.88 (12.75, 15.08) |
|  |  | 150-159 | 2028 | 320 | 19058 | 16.79 (15.00, 18.74) |
|  |  | 160-169 | 751 | 138 | 6896 | 20.01 (16.81, 23.64) |
|  |  | 170-179 | 235 | 57 | 2016 | 28.27 (21.41, 36.63) |
|  |  | ≥180 | 176 | 56 | 1230 | 45.53 (34.39, 59.13) |
|  | 45-59 years | <120 | 34623 | 4658 | 309622 | 15.04 (14.62, 15.48) |
|  |  | 120-129 | 34584 | 4942 | 298706 | 16.54 (16.09, 17.01) |
|  |  | 130-139 | 33360 | 5665 | 291428 | 19.44 (18.94, 19.95) |
|  |  | 140-149 | 23516 | 4947 | 206962 | 23.90 (23.24, 24.58) |
|  |  | 150-159 | 12455 | 3289 | 110394 | 29.79 (28.78, 30.83) |
|  |  | 160-169 | 5073 | 1549 | 44708 | 34.65 (32.94, 36.42) |
|  |  | 170-179 | 1830 | 649 | 15338 | 42.31 (39.12, 45.70) |
|  |  | ≥180 | 1235 | 535 | 9703 | 55.14 (50.57, 60.02) |
|  | 60-74 years | <120 | 24754 | 6576 | 174675 | 37.65 (36.74, 38.57) |
|  |  | 120-129 | 32349 | 8346 | 223781 | 37.30 (36.50, 38.10) |
|  |  | 130-139 | 37494 | 10997 | 261089 | 42.12 (41.34, 42.91) |
|  |  | 140-149 | 30630 | 10249 | 215289 | 47.61 (46.69, 48.54) |
|  |  | 150-159 | 18532 | 7141 | 130172 | 54.86 (53.59, 56.15) |
|  |  | 160-169 | 8517 | 3628 | 58585 | 61.93 (59.93, 63.98) |
|  |  | 170-179 | 3370 | 1560 | 22387 | 69.68 (66.27, 73.23) |
|  |  | ≥180 | 2268 | 1218 | 14544 | 83.75 (79.11, 88.59) |
|  | ≥75 years | <120 | 3979 | 1905 | 20901 | 91.15 (87.10, 95.33) |
|  |  | 120-129 | 5657 | 2625 | 30484 | 86.11 (82.85, 89.47) |
|  |  | 130-139 | 7615 | 3669 | 41024 | 89.44 (86.57, 92.38) |
|  |  | 140-149 | 7146 | 3665 | 38339 | 95.59 (92.52, 98.74) |
|  |  | 150-159 | 5020 | 2721 | 26280 | 103.54 (99.68, 107.50) |
|  |  | 160-169 | 2376 | 1368 | 12019 | 113.82 (107.86, 120.01) |
|  |  | 170-179 | 1089 | 679 | 5337 | 127.22 (117.83, 137.15) |
|  |  | ≥180 | 798 | 517 | 3888 | 132.96 (121.74, 144.93) |
| Kidney failure | All | All | 427098 | 18690 | 3794732 | 4.93 (4.85, 5.00) |
|  | 18-44 years | <120 | 9129 | 214 | 92596 | 2.31 (2.01, 2.64) |
|  |  | 120-129 | 7770 | 173 | 74743 | 2.31 (1.98, 2.69) |
|  |  | 130-139 | 6834 | 178 | 65672 | 2.71 (2.33, 3.14) |
|  |  | 140-149 | 4520 | 194 | 43628 | 4.45 (3.84, 5.12) |
|  |  | 150-159 | 2091 | 121 | 20825 | 5.81 (4.82, 6.94) |
|  |  | 160-169 | 794 | 65 | 7811 | 8.32 (6.42, 10.61) |
|  |  | 170-179 | 248 | 26 | 2347 | 11.08 (7.24, 16.23) |
|  |  | ≥180 | 188 | 28 | 1551 | 18.05 (11.99, 26.09) |
|  | 45-59 years | <120 | 35727 | 719 | 341094 | 2.11 (1.96, 2.27) |
|  |  | 120-129 | 35682 | 732 | 330988 | 2.21 (2.05, 2.38) |
|  |  | 130-139 | 34629 | 869 | 330634 | 2.63 (2.46, 2.81) |
|  |  | 140-149 | 24644 | 944 | 240824 | 3.92 (3.67, 4.18) |
|  |  | 150-159 | 13263 | 794 | 134039 | 5.92 (5.52, 6.35) |
|  |  | 160-169 | 5547 | 467 | 56590 | 8.25 (7.52, 9.04) |
|  |  | 170-179 | 2046 | 249 | 20401 | 12.21 (10.74, 13.82) |
|  |  | ≥180 | 1487 | 267 | 14402 | 18.54 (16.38, 20.90) |
|  | 60-74 years | <120 | 28245 | 836 | 235641 | 3.55 (3.31, 3.80) |
|  |  | 120-129 | 36558 | 998 | 300048 | 3.33 (3.12, 3.54) |
|  |  | 130-139 | 42705 | 1466 | 360256 | 4.07 (3.86, 4.28) |
|  |  | 140-149 | 35698 | 1678 | 313130 | 5.36 (5.11, 5.62) |
|  |  | 150-159 | 22347 | 1484 | 202125 | 7.34 (6.97, 7.73) |
|  |  | 160-169 | 10575 | 908 | 96585 | 9.40 (8.80, 10.03) |
|  |  | 170-179 | 4389 | 479 | 39278 | 12.20 (11.13, 13.34) |
|  |  | ≥180 | 3272 | 583 | 29407 | 19.83 (18.25, 21.50) |
|  | ≥75 years | <120 | 7077 | 379 | 51304 | 7.39 (6.66, 8.17) |
|  |  | 120-129 | 9447 | 428 | 69781 | 6.13 (5.57, 6.74) |
|  |  | 130-139 | 12674 | 771 | 95025 | 8.11 (7.55, 8.71) |
|  |  | 140-149 | 12224 | 872 | 92364 | 9.44 (8.82, 10.09) |
|  |  | 150-159 | 8895 | 798 | 68300 | 11.68 (10.89, 12.52) |
|  |  | 160-169 | 4488 | 445 | 33914 | 13.12 (11.93, 14.40) |
|  |  | 170-179 | 2132 | 249 | 16175 | 15.39 (13.54, 17.43) |
|  |  | ≥180 | 1773 | 276 | 13256 | 20.82 (18.44, 23.43) |
| All-cause death | All | All | 429740 | 83826 | 3865897 | 21.68 (21.54, 21.83) |
|  | 18-44 years | <120 | 9160 | 391 | 93699 | 4.17 (3.77, 4.61) |
|  |  | 120-129 | 7797 | 290 | 75604 | 3.84 (3.41, 4.30) |
|  |  | 130-139 | 6860 | 279 | 66648 | 4.19 (3.71, 4.71) |
|  |  | 140-149 | 4545 | 251 | 44727 | 5.61 (4.94, 6.35) |
|  |  | 150-159 | 2106 | 149 | 21416 | 6.96 (5.89, 8.17) |
|  |  | 160-169 | 806 | 79 | 8211 | 9.62 (7.62, 11.99) |
|  |  | 170-179 | 251 | 24 | 2459 | 9.76 (6.25, 14.52) |
|  |  | ≥180 | 190 | 34 | 1718 | 19.79 (13.70, 27.65) |
|  | 45-59 years | <120 | 35874 | 2820 | 344769 | 8.18 (7.88, 8.49) |
|  |  | 120-129 | 35831 | 2441 | 334704 | 7.29 (7.01, 7.59) |
|  |  | 130-139 | 34824 | 2643 | 335358 | 7.88 (7.58, 8.19) |
|  |  | 140-149 | 24831 | 2390 | 245676 | 9.73 (9.34, 10.13) |
|  |  | 150-159 | 13355 | 1619 | 137663 | 11.76 (11.19, 12.35) |
|  |  | 160-169 | 5601 | 883 | 58724 | 15.04 (14.06, 16.06) |
|  |  | 170-179 | 2081 | 405 | 21664 | 18.69 (16.92, 20.61) |
|  |  | ≥180 | 1521 | 425 | 15727 | 27.02 (24.51, 29.72) |
|  | 60-74 years | <120 | 28398 | 5186 | 238668 | 21.73 (21.14, 22.33) |
|  |  | 120-129 | 36734 | 5883 | 303766 | 19.37 (18.88, 19.87) |
|  |  | 130-139 | 42911 | 7455 | 365550 | 20.39 (19.93, 20.86) |
|  |  | 140-149 | 35948 | 7486 | 319404 | 23.44 (22.91, 23.97) |
|  |  | 150-159 | 22499 | 5557 | 207078 | 26.84 (26.13, 27.55) |
|  |  | 160-169 | 10678 | 3158 | 99774 | 31.65 (30.56, 32.78) |
|  |  | 170-179 | 4443 | 1544 | 40968 | 37.69 (35.83, 39.62) |
|  |  | ≥180 | 3325 | 1418 | 31293 | 45.31 (42.98, 47.73) |
|  | ≥75 years | <120 | 7118 | 3869 | 52253 | 74.04 (71.73, 76.41) |
|  |  | 120-129 | 9511 | 4613 | 70892 | 65.07 (63.21, 66.98) |
|  |  | 130-139 | 12743 | 6107 | 96960 | 62.98 (61.41, 64.58) |
|  |  | 140-149 | 12315 | 6160 | 94517 | 65.17 (63.56, 66.82) |
|  |  | 150-159 | 8966 | 4985 | 70338 | 70.87 (68.92, 72.87) |
|  |  | 160-169 | 4541 | 2653 | 35016 | 75.77 (72.91, 78.70) |
|  |  | 170-179 | 2165 | 1366 | 16792 | 81.35 (77.09, 85.78) |
|  |  | ≥180 | 1812 | 1263 | 13860 | 91.13 (86.17, 96.29) |

**Supplementary Table S4:** Crude incidence (per 1,000 person-year) of cardiovascular disease, kidney disease and all-cause death in individuals with type 2 diabetes, stratified by diastolic blood pressure (DBP) and age.

| **Outcome** | **Age categories** | **DBP categories (mmHg)** | **Number of people at risk** | **Events** | **Total person-year** | **Crude incidence per 1,000 person-year (95% CI)** |
| --- | --- | --- | --- | --- | --- | --- |
| Cardiovascular disease | All | All | 354350 | 37382 | 3092079 | 12.09 (11.97, 12.21) |
|  | 18-44 years | <60 | 999 | 30 | 9807 | 3.06 (2.06, 4.37) |
|  |  | 60-69 | 4540 | 172 | 45880 | 3.75 (3.21, 4.35) |
|  |  | 70-79 | 9803 | 480 | 96719 | 4.96 (4.53, 5.43) |
|  |  | 80-89 | 9598 | 610 | 91885 | 6.64 (6.12, 7.19) |
|  |  | 90-99 | 4202 | 308 | 38278 | 8.05 (7.17, 9.00) |
|  |  | 100-109 | 868 | 77 | 7324 | 10.51 (8.30, 13.14) |
|  |  | ≥110 | 211 | 34 | 1689 | 20.13 (13.94, 28.13) |
|  | 45-59 years | <60 | 4265 | 256 | 38296 | 6.68 (5.89, 7.56) |
|  |  | 60-69 | 21174 | 1443 | 200358 | 7.20 (6.84, 7.58) |
|  |  | 70-79 | 47180 | 3614 | 444018 | 8.14 (7.88, 8.41) |
|  |  | 80-89 | 44193 | 3721 | 417137 | 8.92 (8.64, 9.21) |
|  |  | 90-99 | 16472 | 1637 | 153330 | 10.68 (10.17, 11.21) |
|  |  | 100-109 | 2541 | 351 | 22798 | 15.40 (13.83, 17.09) |
|  |  | ≥110 | 399 | 83 | 3350 | 24.78 (19.74, 30.72) |
|  | 60-74 years | <60 | 7602 | 861 | 59212 | 14.54 (13.59, 15.55) |
|  |  | 60-69 | 31490 | 3516 | 259865 | 13.53 (13.09, 13.98) |
|  |  | 70-79 | 55742 | 6153 | 464898 | 13.24 (12.91, 13.57) |
|  |  | 80-89 | 39490 | 4606 | 331412 | 13.90 (13.50, 14.31) |
|  |  | 90-99 | 11064 | 1499 | 92372 | 16.23 (15.42, 17.07) |
|  |  | 100-109 | 1481 | 240 | 11719 | 20.48 (17.97, 23.24) |
|  |  | ≥110 | 251 | 54 | 1853 | 29.15 (21.90, 38.03) |
|  | ≥75 years | <60 | 4943 | 937 | 33810 | 27.71 (25.97, 29.55) |
|  |  | 60-69 | 13126 | 2332 | 95861 | 24.33 (23.35, 25.33) |
|  |  | 70-79 | 13977 | 2557 | 104868 | 24.38 (23.45, 25.35) |
|  |  | 80-89 | 6899 | 1366 | 51715 | 26.41 (25.03, 27.85) |
|  |  | 90-99 | 1579 | 373 | 11817 | 31.56 (28.44, 34.94) |
|  |  | 100-109 | 218 | 60 | 1526 | 39.31 (30.00, 50.60) |
|  |  | ≥110 | 43 | 12 | 283 | 42.40 (21.91, 74.07) |
| Chronic kidney disease | All | All | 369347 | 96095 | 2858138 | 33.62 (33.41, 33.83) |
|  | 18-44 years | <60 | 1015 | 35 | 9974 | 3.51 (2.44, 4.88) |
|  |  | 60-69 | 4655 | 310 | 46371 | 6.69 (5.96, 7.47) |
|  |  | 70-79 | 10056 | 841 | 97370 | 8.64 (8.06, 9.24) |
|  |  | 80-89 | 9856 | 1053 | 91684 | 11.49 (10.80, 12.20) |
|  |  | 90-99 | 4358 | 554 | 38106 | 14.54 (13.35, 15.80) |
|  |  | 100-109 | 913 | 158 | 7302 | 21.64 (18.40, 25.29) |
|  |  | ≥110 | 224 | 46 | 1679 | 27.40 (20.06, 36.55) |
|  | 45-59 years | <60 | 4536 | 594 | 38942 | 15.25 (14.05, 16.53) |
|  |  | 60-69 | 22774 | 3343 | 204477 | 16.35 (15.80, 16.91) |
|  |  | 70-79 | 50754 | 8281 | 450305 | 18.39 (18.00, 18.79) |
|  |  | 80-89 | 47593 | 9057 | 417428 | 21.70 (21.25, 22.15) |
|  |  | 90-99 | 17811 | 4045 | 150435 | 26.89 (26.07, 27.73) |
|  |  | 100-109 | 2764 | 756 | 22021 | 34.33 (31.93, 36.87) |
|  |  | ≥110 | 444 | 158 | 3252 | 48.58 (41.30, 56.77) |
|  | 60-74 years | <60 | 8154 | 2565 | 53277 | 48.14 (46.30, 50.04) |
|  |  | 60-69 | 33903 | 10566 | 236348 | 44.71 (43.86, 45.57) |
|  |  | 70-79 | 59735 | 18194 | 421732 | 43.14 (42.52, 43.77) |
|  |  | 80-89 | 42396 | 13475 | 297242 | 45.33 (44.57, 46.11) |
|  |  | 90-99 | 11858 | 4137 | 80577 | 51.34 (49.79, 52.93) |
|  |  | 100-109 | 1599 | 650 | 9973 | 65.18 (60.26, 70.39) |
|  |  | ≥110 | 269 | 128 | 1373 | 93.23 (77.78, 110.85) |
|  | ≥75 years | <60 | 3951 | 1986 | 19514 | 101.77 (97.35, 106.35) |
|  |  | 60-69 | 10749 | 5455 | 56930 | 95.82 (93.29, 98.40) |
|  |  | 70-79 | 11756 | 5895 | 63910 | 92.24 (89.90, 94.63) |
|  |  | 80-89 | 5770 | 2990 | 30724 | 97.32 (93.86, 100.87) |
|  |  | 90-99 | 1265 | 713 | 6417 | 111.12 (103.11, 119.58) |
|  |  | 100-109 | 159 | 95 | 644 | 147.48 (119.32, 180.28) |
|  |  | ≥110 | 30 | 15 | 134 | 111.59 (62.46, 184.06) |
| Kidney failure | All | All | 427098 | 18690 | 3794732 | 4.93 (4.85, 5.00) |
|  | 18-44 years | <60 | 1025 | 11 | 10191 | 1.08 (0.54, 1.93) |
|  |  | 60-69 | 4711 | 100 | 48087 | 2.08 (1.69, 2.53) |
|  |  | 70-79 | 10178 | 272 | 101960 | 2.67 (2.36, 3.00) |
|  |  | 80-89 | 10024 | 346 | 97532 | 3.55 (3.18, 3.94) |
|  |  | 90-99 | 4461 | 200 | 41293 | 4.84 (4.20, 5.56) |
|  |  | 100-109 | 942 | 46 | 8186 | 5.62 (4.11, 7.50) |
|  |  | ≥110 | 233 | 24 | 1924 | 12.48 (7.99, 18.56) |
|  | 45-59 years | <60 | 4731 | 125 | 43289 | 2.89 (2.40, 3.44) |
|  |  | 60-69 | 23650 | 625 | 228188 | 2.74 (2.53, 2.96) |
|  |  | 70-79 | 52656 | 1494 | 507142 | 2.95 (2.80, 3.10) |
|  |  | 80-89 | 49636 | 1648 | 479263 | 3.44 (3.27, 3.61) |
|  |  | 90-99 | 18811 | 877 | 178855 | 4.90 (4.58, 5.24) |
|  |  | 100-109 | 3026 | 213 | 27808 | 7.66 (6.67, 8.76) |
|  |  | ≥110 | 515 | 59 | 4428 | 13.32 (10.14, 17.19) |
|  | 60-74 years | <60 | 9918 | 522 | 79795 | 6.54 (5.99, 7.13) |
|  |  | 60-69 | 39712 | 1868 | 339593 | 5.50 (5.25, 5.76) |
|  |  | 70-79 | 68866 | 2870 | 593798 | 4.83 (4.66, 5.01) |
|  |  | 80-89 | 48972 | 2215 | 424121 | 5.22 (5.01, 5.44) |
|  |  | 90-99 | 14009 | 770 | 120509 | 6.39 (5.95, 6.86) |
|  |  | 100-109 | 1955 | 154 | 15970 | 9.64 (8.18, 11.29) |
|  |  | ≥110 | 357 | 33 | 2685 | 12.29 (8.46, 17.26) |
|  | ≥75 years | <60 | 7575 | 617 | 52184 | 11.82 (10.91, 12.79) |
|  |  | 60-69 | 18776 | 1332 | 140123 | 9.51 (9.00, 10.03) |
|  |  | 70-79 | 19812 | 1348 | 152088 | 8.86 (8.40, 9.35) |
|  |  | 80-89 | 9837 | 696 | 75378 | 9.23 (8.56, 9.95) |
|  |  | 90-99 | 2313 | 180 | 17447 | 10.32 (8.86, 11.94) |
|  |  | 100-109 | 328 | 39 | 2434 | 16.03 (11.40, 21.91) |
|  |  | ≥110 | 69 | 6 | 464 | 12.94 (4.75, 28.18) |
| All-cause death | All | All | 429740 | 83826 | 3865897 | 21.68 (21.54, 21.83) |
|  | 18-44 years | <60 | 1030 | 44 | 10281 | 4.28 (3.11, 5.75) |
|  |  | 60-69 | 4728 | 187 | 48685 | 3.84 (3.31, 4.43) |
|  |  | 70-79 | 10218 | 431 | 103352 | 4.17 (3.79, 4.58) |
|  |  | 80-89 | 10073 | 496 | 99398 | 4.99 (4.56, 5.45) |
|  |  | 90-99 | 4485 | 261 | 42342 | 6.16 (5.44, 6.96) |
|  |  | 100-109 | 948 | 51 | 8389 | 6.08 (4.53, 7.99) |
|  |  | ≥110 | 233 | 27 | 2034 | 13.28 (8.75, 19.32) |
|  | 45-59 years | <60 | 4767 | 469 | 43921 | 10.68 (9.73, 11.69) |
|  |  | 60-69 | 23785 | 2077 | 231479 | 8.97 (8.59, 9.37) |
|  |  | 70-79 | 52941 | 4365 | 514771 | 8.48 (8.23, 8.73) |
|  |  | 80-89 | 49938 | 4239 | 487672 | 8.69 (8.43, 8.96) |
|  |  | 90-99 | 18915 | 1975 | 182926 | 10.80 (10.33, 11.28) |
|  |  | 100-109 | 3052 | 389 | 28827 | 13.49 (12.19, 14.90) |
|  |  | ≥110 | 520 | 112 | 4689 | 23.89 (19.67, 28.74) |
|  | 60-74 years | <60 | 10033 | 2453 | 81710 | 30.02 (28.84, 31.23) |
|  |  | 60-69 | 40002 | 8722 | 346161 | 25.20 (24.67, 25.73) |
|  |  | 70-79 | 69249 | 13283 | 604294 | 21.98 (21.61, 22.36) |
|  |  | 80-89 | 49250 | 9587 | 431990 | 22.19 (21.75, 22.64) |
|  |  | 90-99 | 14073 | 3011 | 123070 | 24.47 (23.60, 25.36) |
|  |  | 100-109 | 1969 | 521 | 16498 | 31.58 (28.93, 34.41) |
|  |  | ≥110 | 360 | 110 | 2779 | 39.58 (32.53, 47.71) |
|  | ≥75 years | <60 | 7660 | 4276 | 53671 | 79.67 (77.30, 82.09) |
|  |  | 60-69 | 18945 | 9926 | 143566 | 69.14 (67.79, 70.51) |
|  |  | 70-79 | 19921 | 10026 | 155364 | 64.53 (63.28, 65.81) |
|  |  | 80-89 | 9909 | 5220 | 77098 | 67.71 (65.88, 69.57) |
|  |  | 90-99 | 2334 | 1325 | 17928 | 73.91 (69.98, 78.00) |
|  |  | 100-109 | 331 | 197 | 2515 | 78.32 (67.76, 90.05) |
|  |  | ≥110 | 71 | 46 | 486 | 94.67 (69.31, 126.27) |

**Supplementary Table S5:** Cox regression models for the associations of different systolic blood pressure (SBP) cut-offs (referenced to SBP 120 – 129 mmHg) with incident events among adults with type 2 diabetes, stratified by age groups.

| Age | SBP (mmHg) | Cardiovascular disease | | Chronic kidney disease | | Kidney failure | | All-cause death | |
| --- | --- | --- | --- | --- | --- | --- | --- | --- | --- |
|  |  | HR (95% CI) | P-value | HR (95% CI) | P-value | HR (95% CI) | P-value | HR (95% CI) | P-value |
| 18-44 years | <120 | 0.95 (0.82, 1.11) | 0.516 | 0.95 (0.85, 1.06) | 0.341 | 0.91 (0.74, 1.12) | 0.365 | 1.08 (0.93, 1.26) | 0.303 |
|  | 120-129 | Ref |  | Ref |  | Ref |  | Ref |  |
|  | 130-139 | 1.05 (0.90, 1.21) | 0.545 | 1.11 (0.99, 1.24) | 0.071 | 1.11 (0.90, 1.37) | 0.337 | 1.01 (0.85, 1.19) | 0.918 |
|  | 140-149 | 1.23 (1.05, 1.44) | 0.009 | 1.38 (1.23, 1.55) | <0.001 | 1.57 (1.27, 1.93) | <0.001 | 1.23 (1.04, 1.47) | 0.016 |
|  | 150-159 | 1.39 (1.16, 1.67) | <0.001 | 1.52 (1.32, 1.75) | <0.001 | 1.78 (1.40, 2.26) | <0.001 | 1.41 (1.15, 1.72) | <0.001 |
|  | 160-169 | 2.00 (1.59, 2.50) | <0.001 | 1.65 (1.36, 1.99) | <0.001 | 1.83 (1.36, 2.46) | <0.001 | 1.63 (1.27, 2.11) | <0.001 |
|  | 170-179 | 2.01 (1.37, 2.95) | <0.001 | 2.13 (1.62, 2.80) | <0.001 | 3.11 (2.05, 4.73) | <0.001 | 1.87 (1.23, 2.84) | 0.004 |
|  | ≥180 | 2.53 (1.72, 3.74) | <0.001 | 3.17 (2.40, 4.19) | <0.001 | 3.38 (2.24, 5.11) | <0.001 | 2.80 (1.95, 4.03) | <0.001 |
| 45-59 years | <120 | 0.99 (0.93, 1.05) | 0.636 | 0.99 (0.95, 1.03) | 0.739 | 1.01 (0.91, 1.12) | 0.858 | 1.12 (1.06, 1.19) | <0.001 |
|  | 120-129 | Ref |  | Ref |  | Ref |  | Ref |  |
|  | 130-139 | 1.07 (1.01, 1.14) | 0.021 | 1.05 (1.01, 1.10) | 0.007 | 1.02 (0.92, 1.12) | 0.715 | 0.99 (0.94, 1.05) | 0.796 |
|  | 140-149 | 1.17 (1.10, 1.25) | <0.001 | 1.17 (1.13, 1.22) | <0.001 | 1.34 (1.22, 1.48) | <0.001 | 1.13 (1.07, 1.20) | <0.001 |
|  | 150-159 | 1.26 (1.17, 1.35) | <0.001 | 1.34 (1.28, 1.40) | <0.001 | 1.59 (1.43, 1.76) | <0.001 | 1.23 (1.15, 1.31) | <0.001 |
|  | 160-169 | 1.35 (1.23, 1.48) | <0.001 | 1.46 (1.37, 1.54) | <0.001 | 1.76 (1.57, 1.98) | <0.001 | 1.41 (1.31, 1.53) | <0.001 |
|  | 170-179 | 1.66 (1.46, 1.88) | <0.001 | 1.65 (1.52, 1.80) | <0.001 | 2.34 (2.03, 2.71) | <0.001 | 1.61 (1.44, 1.79) | <0.001 |
|  | ≥180 | 1.97 (1.73, 2.24) | <0.001 | 2.07 (1.89, 2.26) | <0.001 | 2.69 (2.34, 3.11) | <0.001 | 2.08 (1.87, 2.31) | <0.001 |
| 60-74 years | <120 | 0.99 (0.94, 1.05) | 0.832 | 1.04 (1.00, 1.07) | 0.028 | 1.04 (0.95, 1.14) | 0.371 | 1.07 (1.03, 1.11) | <0.001 |
|  | 120-129 | Ref |  | Ref |  | Ref |  | Ref |  |
|  | 130-139 | 0.98 (0.93, 1.03) | 0.477 | 1.05 (1.02, 1.08) | 0.001 | 1.07 (0.98, 1.16) | 0.114 | 0.98 (0.94, 1.01) | 0.191 |
|  | 140-149 | 1.05 (1.00, 1.10) | 0.065 | 1.10 (1.07, 1.13) | <0.001 | 1.26 (1.17, 1.37) | <0.001 | 1.03 (1.00, 1.07) | 0.061 |
|  | 150-159 | 1.13 (1.07, 1.19) | <0.001 | 1.17 (1.13, 1.21) | <0.001 | 1.42 (1.31, 1.54) | <0.001 | 1.07 (1.03, 1.11) | <0.001 |
|  | 160-169 | 1.20 (1.12, 1.28) | <0.001 | 1.22 (1.17, 1.26) | <0.001 | 1.56 (1.42, 1.70) | <0.001 | 1.16 (1.11, 1.21) | <0.001 |
|  | 170-179 | 1.29 (1.18, 1.41) | <0.001 | 1.34 (1.27, 1.42) | <0.001 | 1.83 (1.64, 2.04) | <0.001 | 1.34 (1.26, 1.42) | <0.001 |
|  | ≥180 | 1.49 (1.36, 1.64) | <0.001 | 1.48 (1.39, 1.57) | <0.001 | 2.18 (1.97, 2.42) | <0.001 | 1.42 (1.34, 1.50) | <0.001 |
| ≥75 years | <120 | 1.15 (1.05, 1.25) | 0.003 | 1.05 (0.99, 1.12) | 0.098 | 1.15 (1.00, 1.32) | 0.051 | 1.09 (1.04, 1.13) | <0.001 |
|  | 120-129 | Ref |  | Ref |  | Ref |  | Ref |  |
|  | 130-139 | 1.07 (0.99, 1.16) | 0.098 | 1.01 (0.96, 1.07) | 0.605 | 1.29 (1.14, 1.45) | <0.001 | 0.95 (0.92, 0.99) | 0.015 |
|  | 140-149 | 1.07 (0.99, 1.16) | 0.080 | 1.06 (1.00, 1.11) | 0.033 | 1.37 (1.22, 1.54) | <0.001 | 0.96 (0.93, 1.00) | 0.065 |
|  | 150-159 | 1.08 (0.99, 1.17) | 0.075 | 1.10 (1.04, 1.16) | <0.001 | 1.52 (1.35, 1.71) | <0.001 | 1.00 (0.96, 1.04) | 0.828 |
|  | 160-169 | 1.26 (1.14, 1.38) | <0.001 | 1.20 (1.12, 1.28) | <0.001 | 1.51 (1.32, 1.72) | <0.001 | 1.05 (1.00, 1.10) | 0.072 |
|  | 170-179 | 1.32 (1.17, 1.48) | <0.001 | 1.28 (1.17, 1.39) | <0.001 | 1.64 (1.40, 1.92) | <0.001 | 1.06 (1.00, 1.13) | 0.048 |
|  | ≥180 | 1.35 (1.19, 1.53) | <0.001 | 1.28 (1.16, 1.41) | <0.001 | 1.95 (1.67, 2.27) | <0.001 | 1.18 (1.10, 1.25) | <0.001 |

The analysis is adjusted for calendar year at assessment, age, sex, diabetes duration, BMI, smoking, HbA1c, LDL cholesterol, HDL cholesterol, log triglycerides, presence of albuminuria, history of CKD, history of CVD and use of blood pressure lowering medications.

**Supplementary Table S6:** Cox regression models for the associations of different diastolic blood pressure (DBP) cut-offs (referenced to DBP 70 – 79 mmHg) with incident events, stratified by age groups.

| Age group | DBP  (mmHg) | Cardiovascular disease | | Chronic kidney disease | | Kidney failure | | All-cause death | |
| --- | --- | --- | --- | --- | --- | --- | --- | --- | --- |
|  |  | HR (95% CI) | P-value | HR (95% CI) | P-value | HR (95% CI) | P-value | HR (95% CI) | P-value |
| 18-44 years | <60 | 0.96 (0.66, 1.40) | 0.842 | 0.55 (0.39, 0.77) | <0.001 | 0.49 (0.27, 0.90) | 0.022 | 1.25 (0.91, 1.71) | 0.164 |
|  | 60-69 | 0.91 (0.76, 1.08) | 0.291 | 0.88 (0.77, 1.00) | 0.047 | 0.82 (0.65, 1.03) | 0.089 | 0.97 (0.82, 1.16) | 0.767 |
|  | 70-79 | Ref |  | Ref |  | Ref |  | Ref |  |
|  | 80-89 | 1.14 (1.01, 1.28) | 0.038 | 1.15 (1.05, 1.26) | 0.003 | 1.17 (1.00, 1.38) | 0.051 | 1.09 (0.96, 1.24) | 0.190 |
|  | 90-99 | 1.24 (1.07, 1.44) | 0.003 | 1.30 (1.17, 1.46) | <0.001 | 1.43 (1.18, 1.72) | <0.001 | 1.25 (1.07, 1.46) | 0.006 |
|  | 100-109 | 1.56 (1.22, 2.00) | <0.001 | 1.76 (1.48, 2.09) | <0.001 | 1.41 (1.03, 1.94) | 0.034 | 1.21 (0.90, 1.62) | 0.210 |
|  | ≥110 | 2.39 (1.68, 3.40) | <0.001 | 1.78 (1.32, 2.40) | <0.001 | 1.98 (1.29, 3.03) | 0.002 | 2.01 (1.35, 2.98) | <0.001 |
| 45-59 years | <60 | 1.06 (0.94, 1.21) | 0.342 | 1.01 (0.93, 1.10) | 0.821 | 1.13 (0.94, 1.36) | 0.181 | 1.41 (1.28, 1.55) | <0.001 |
|  | 60-69 | 0.98 (0.92, 1.04) | 0.470 | 0.98 (0.94, 1.02) | 0.223 | 0.97 (0.88, 1.06) | 0.503 | 1.09 (1.03, 1.15) | 0.001 |
|  | 70-79 | Ref |  | Ref |  | Ref |  | Ref |  |
|  | 80-89 | 1.00 (0.96, 1.05) | 0.960 | 1.07 (1.04, 1.10) | <0.001 | 1.02 (0.95, 1.09) | 0.651 | 0.97 (0.93, 1.01) | 0.140 |
|  | 90-99 | 1.09 (1.03, 1.16) | 0.005 | 1.21 (1.16, 1.25) | <0.001 | 1.20 (1.10, 1.31) | <0.001 | 1.12 (1.06, 1.18) | <0.001 |
|  | 100-109 | 1.40 (1.25, 1.56) | <0.001 | 1.44 (1.34, 1.55) | <0.001 | 1.23 (1.06, 1.42) | 0.006 | 1.20 (1.08, 1.33) | <0.001 |
|  | ≥110 | 2.02 (1.62, 2.51) | <0.001 | 1.67 (1.42, 1.95) | <0.001 | 1.88 (1.45, 2.44) | <0.001 | 1.84 (1.53, 2.22) | <0.001 |
| 60-74 years | <60 | 1.13 (1.05, 1.21) | 0.001 | 1.13 (1.08, 1.18) | <0.001 | 1.11 (1.01, 1.22) | 0.035 | 1.24 (1.19, 1.30) | <0.001 |
|  | 60-69 | 1.03 (0.98, 1.07) | 0.239 | 1.03 (1.00, 1.05) | 0.033 | 1.10 (1.04, 1.17) | 0.001 | 1.09 (1.06, 1.12) | <0.001 |
|  | 70-79 | Ref |  | Ref |  | Ref |  | Ref |  |
|  | 80-89 | 1.01 (0.97, 1.05) | 0.521 | 1.02 (0.99, 1.04) | 0.167 | 1.05 (0.99, 1.11) | 0.095 | 1.02 (0.99, 1.04) | 0.197 |
|  | 90-99 | 1.14 (1.07, 1.20) | <0.001 | 1.12 (1.08, 1.16) | <0.001 | 1.10 (1.01, 1.19) | 0.020 | 1.09 (1.05, 1.14) | <0.001 |
|  | 100-109 | 1.34 (1.18, 1.53) | <0.001 | 1.33 (1.23, 1.44) | <0.001 | 1.34 (1.14, 1.58) | <0.001 | 1.32 (1.21, 1.44) | <0.001 |
|  | ≥110 | 1.87 (1.43, 2.45) | <0.001 | 2.01 (1.69, 2.40) | <0.001 | 1.53 (1.08, 2.15) | 0.016 | 1.72 (1.42, 2.07) | <0.001 |
| ≥75 years | <60 | 1.12 (1.04, 1.21) | 0.003 | 1.10 (1.05, 1.16) | <0.001 | 1.21 (1.10, 1.33) | <0.001 | 1.17 (1.13, 1.22) | <0.001 |
|  | 60-69 | 0.99 (0.94, 1.05) | 0.798 | 1.04 (1.00, 1.08) | 0.028 | 1.06 (0.98, 1.14) | 0.129 | 1.05 (1.02, 1.08) | <0.001 |
|  | 70-79 | Ref |  | Ref |  | Ref |  | Ref |  |
|  | 80-89 | 1.05 (0.98, 1.12) | 0.137 | 1.04 (0.99, 1.08) | 0.109 | 1.00 (0.91, 1.10) | 0.997 | 1.05 (1.02, 1.09) | 0.003 |
|  | 90-99 | 1.17 (1.05, 1.31) | 0.005 | 1.14 (1.06, 1.24) | <0.001 | 0.94 (0.80, 1.09) | 0.405 | 1.11 (1.05, 1.18) | <0.001 |
|  | 100-109 | 1.39 (1.08, 1.80) | 0.011 | 1.44 (1.18, 1.77) | <0.001 | 1.18 (0.86, 1.63) | 0.305 | 1.13 (0.98, 1.31) | 0.080 |
|  | ≥110 | 1.59 (0.90, 2.80) | 0.112 | 0.94 (0.56, 1.56) | 0.800 | 1.12 (0.50, 2.49) | 0.788 | 1.48 (1.11, 1.98) | 0.008 |

The analysis is adjusted for calendar year at assessment, age, sex, diabetes duration, BMI, smoking, HbA1c, LDL cholesterol, HDL cholesterol, log triglycerides, presence of albuminuria, history of CKD, history of CVD and use of blood pressure lowering medications.

**Supplementary Table S7:** Cox regression models for the effect of each 10 mmHg increase in systolic blood pressure (SBP) and diastolic blood pressure (DBP) on incident events, stratified by age groups.

| Outcomes | Per 10mmHg increase in SBP / DBP | 18-44 years | | 45-59 years | | 60-74 years | | ≥75 years | | P-interaction |
| --- | --- | --- | --- | --- | --- | --- | --- | --- | --- | --- |
|  |  | HR (95% CI) | P-value | HR (95% CI) | P-value | HR (95% CI) | P-value | HR (95% CI) | P-value |  |
| Cardiovascular disease | SBP | 1.16 (1.11, 1.20) | <0.001 | 1.10 (1.09, 1.12) | <0.001 | 1.06 (1.05, 1.07) | <0.001 | 1.05 (1.03, 1.06) | <0.001 | <0.001 |
|  | DBP | 1.17 (1.10, 1.23) | <0.001 | 1.08 (1.05, 1.11) | <0.001 | 1.06 (1.04, 1.09) | <0.001 | 1.08 (1.03, 1.12) | <0.001 | <0.001 |
| Coronary Heart disease | SBP | 1.13 (1.08, 1.18) | <0.001 | 1.06 (1.05, 1.08) | <0.001 | 1.05 (1.04, 1.06) | <0.001 | 1.05 (1.03, 1.08) | <0.001 | <0.001 |
|  | DBP | 1.14 (1.06, 1.22) | <0.001 | 1.05 (1.02, 1.09) | <0.001 | 1.00 (0.97, 1.03) | 0.844 | 1.02 (0.96, 1.08) | 0.477 | <0.001 |
| Peripheral vascular disease | SBP | 1.13 (1.04, 1.22) | 0.002 | 1.16 (1.13, 1.19) | <0.001 | 1.06 (1.04, 1.09) | <0.001 | 1.06 (1.02, 1.09) | <0.001 | <0.001 |
|  | DBP | 1.05 (0.93, 1.19) | 0.448 | 1.01 (0.96, 1.08) | 0.627 | 0.98 (0.93, 1.04) | 0.540 | 1.12 (1.03, 1.23) | 0.010 | 0.102 |
| Stroke | SBP | 1.23 (1.16, 1.30) | <0.001 | 1.11 (1.09, 1.14) | <0.001 | 1.07 (1.05, 1.08) | <0.001 | 1.04 (1.03, 1.06) | <0.001 | <0.001 |
|  | DBP | 1.34 (1.23, 1.47) | <0.001 | 1.13 (1.09, 1.17) | <0.001 | 1.14 (1.11, 1.18) | <0.001 | 1.13 (1.08, 1.18) | <0.001 | <0.001 |
| Ischemic stroke | SBP | 1.20 (1.12, 1.30) | <0.001 | 1.11 (1.08, 1.14) | <0.001 | 1.07 (1.06, 1.09) | <0.001 | 1.05 (1.03, 1.07) | <0.001 | <0.001 |
|  | DBP | 1.32 (1.18, 1.49) | <0.001 | 1.12 (1.07, 1.17) | <0.001 | 1.13 (1.09, 1.17) | <0.001 | 1.14 (1.08, 1.21) | <0.001 | 0.089 |
| Haemorrhagic stroke | SBP | 1.20 (1.08, 1.32) | <0.001 | 1.15 (1.10, 1.19) | <0.001 | 1.06 (1.03, 1.08) | <0.001 | 1.01 (0.98, 1.04) | 0.645 | <0.001 |
|  | DBP | 1.47 (1.26, 1.71) | <0.001 | 1.23 (1.15, 1.32) | <0.001 | 1.19 (1.13, 1.26) | <0.001 | 1.15 (1.07, 1.25) | <0.001 | <0.001 |
| Chronic Kidney Disease | SBP | 1.17 (1.14, 1.20) | <0.001 | 1.12 (1.11, 1.13) | <0.001 | 1.06 (1.05, 1.07) | <0.001 | 1.05 (1.03, 1.06) | <0.001 | <0.001 |
|  | DBP | 1.19 (1.14, 1.24) | <0.001 | 1.11 (1.09, 1.13) | <0.001 | 1.06 (1.05, 1.08) | <0.001 | 1.07 (1.04, 1.10) | <0.001 | <0.001 |
| Kidney failure | SBP | 1.19 (1.14, 1.25) | <0.001 | 1.17 (1.15, 1.19) | <0.001 | 1.12 (1.11, 1.14) | <0.001 | 1.09 (1.07, 1.11) | <0.001 | <0.001 |
|  | DBP | 1.19 (1.11, 1.28) | <0.001 | 1.09 (1.06, 1.13) | <0.001 | 1.06 (1.03, 1.10) | <0.001 | 1.01 (0.95, 1.07) | 0.764 | <0.001 |
| All-cause death | SBP | 1.14 (1.10, 1.19) | <0.001 | 1.11 (1.09, 1.12) | <0.001 | 1.05 (1.04, 1.06) | <0.001 | 1.02 (1.01, 1.03) | <0.001 | <0.001 |
|  | DBP | 1.13 (1.06, 1.20) | <0.001 | 1.07 (1.04, 1.09) | <0.001 | 1.05 (1.03, 1.07) | <0.001 | 1.05 (1.03, 1.07) | <0.001 | <0.001 |

The analysis adjusted for calendar year at assessment, age, sex, diabetes duration, BMI, smoking, HbA1c, LDL cholesterol, HDL cholesterol, log triglycerides, presence of albuminuria, history of CKD, history of CVD and use of blood pressure lowering medications. Systolic blood pressure and diastolic blood pressure were included in the model separately. The analyses were limited to individuals with SBP ≥120 mmHg (n = 349,190) or DBP ≥ 70 mmHg (n = 318,790).

**Supplementary Table S8:** Cox regression models for the effect of each 1 standard deviation (SD) increase in systolic blood pressure (SBP) and diastolic blood pressure (DBP) on incident events, stratified by age groups.

| Outcomes | Per 1SD increase in SBP / DBP | 18-44 years | | 45-59 years | | 60-74 years | | ≥75 years | | P-interaction |
| --- | --- | --- | --- | --- | --- | --- | --- | --- | --- | --- |
|  |  | HR (95% CI) | P-value | HR (95% CI) | P-value | HR (95% CI) | P-value | HR (95% CI) | P-value |  |
| Cardiovascular disease | SBP | 1.30 (1.21, 1.38) | <0.001 | 1.19 (1.16, 1.22) | <0.001 | 1.11 (1.09, 1.13) | <0.001 | 1.09 (1.06, 1.12) | <0.001 | <0.001 |
|  | DBP | 1.18 (1.11, 1.25) | <0.001 | 1.08 (1.06, 1.11) | <0.001 | 1.07 (1.04, 1.09) | <0.001 | 1.08 (1.04, 1.13) | <0.001 | <0.001 |
| Coronary Heart disease | SBP | 1.24 (1.14, 1.35) | <0.001 | 1.12 (1.08, 1.15) | <0.001 | 1.09 (1.07, 1.12) | <0.001 | 1.10 (1.06, 1.14) | <0.001 | <0.001 |
|  | DBP | 1.15 (1.06, 1.24) | <0.001 | 1.06 (1.02, 1.09) | <0.001 | 1.00 (0.97, 1.04) | 0.844 | 1.02 (0.96, 1.09) | 0.477 | <0.001 |
| Peripheral vascular disease | SBP | 1.24 (1.08, 1.42) | 0.002 | 1.31 (1.24, 1.38) | <0.001 | 1.12 (1.07, 1.17) | <0.001 | 1.10 (1.04, 1.17) | <0.001 | <0.001 |
|  | DBP | 1.05 (0.92, 1.20) | 0.448 | 1.02 (0.95, 1.08) | 0.627 | 0.98 (0.92, 1.04) | 0.540 | 1.13 (1.03, 1.25) | 0.010 | 0.102 |
| Stroke | SBP | 1.45 (1.31, 1.61) | <0.001 | 1.21 (1.17, 1.26) | <0.001 | 1.12 (1.10, 1.15) | <0.001 | 1.08 (1.05, 1.11) | <0.001 | <0.001 |
|  | DBP | 1.37 (1.25, 1.51) | <0.001 | 1.14 (1.09, 1.18) | <0.001 | 1.16 (1.12, 1.19) | <0.001 | 1.14 (1.08, 1.20) | <0.001 | <0.001 |
| Ischemic stroke | SBP | 1.39 (1.22, 1.59) | <0.001 | 1.21 (1.15, 1.26) | <0.001 | 1.13 (1.10, 1.17) | <0.001 | 1.09 (1.05, 1.13) | <0.001 | <0.001 |
|  | DBP | 1.35 (1.19, 1.53) | <0.001 | 1.13 (1.08, 1.19) | <0.001 | 1.14 (1.10, 1.19) | <0.001 | 1.15 (1.08, 1.23) | <0.001 | 0.089 |
| Haemorrhagic stroke | SBP | 1.38 (1.15, 1.65) | <0.001 | 1.28 (1.20, 1.37) | <0.001 | 1.10 (1.06, 1.15) | <0.001 | 1.01 (0.96, 1.07) | 0.645 | <0.001 |
|  | DBP | 1.51 (1.28, 1.79) | <0.001 | 1.25 (1.16, 1.35) | <0.001 | 1.21 (1.14, 1.28) | <0.001 | 1.17 (1.07, 1.27) | <0.001 | <0.001 |
| Chronic Kidney Disease | SBP | 1.33 (1.26, 1.39) | <0.001 | 1.22 (1.20, 1.24) | <0.001 | 1.11 (1.10, 1.12) | <0.001 | 1.08 (1.06, 1.10) | <0.001 | <0.001 |
|  | DBP | 1.21 (1.15, 1.26) | <0.001 | 1.12 (1.10, 1.14) | <0.001 | 1.07 (1.05, 1.08) | <0.001 | 1.07 (1.04, 1.11) | <0.001 | <0.001 |
| Kidney failure | SBP | 1.37 (1.27, 1.48) | <0.001 | 1.33 (1.29, 1.37) | <0.001 | 1.23 (1.20, 1.26) | <0.001 | 1.17 (1.13, 1.20) | <0.001 | <0.001 |
|  | DBP | 1.21 (1.12, 1.30) | <0.001 | 1.10 (1.06, 1.14) | <0.001 | 1.07 (1.03, 1.11) | <0.001 | 1.01 (0.95, 1.07) | 0.764 | <0.001 |
| All-cause death | SBP | 1.27 (1.18, 1.36) | <0.001 | 1.20 (1.17, 1.22) | <0.001 | 1.09 (1.08, 1.11) | <0.001 | 1.04 (1.02, 1.05) | <0.001 | <0.001 |
|  | DBP | 1.14 (1.07, 1.22) | <0.001 | 1.07 (1.05, 1.10) | <0.001 | 1.05 (1.04, 1.07) | <0.001 | 1.05 (1.03, 1.07) | <0.001 | <0.001 |

The analysis adjusted for calendar year at assessment, age, sex, diabetes duration, BMI, smoking, HbA1c, LDL cholesterol, HDL cholesterol, log triglycerides, presence of albuminuria, history of CKD, history of CVD and use of blood pressure lowering medications. Systolic blood pressure and diastolic blood pressure were included in the model separately. The analyses were limited to individuals with SBP ≥120 mmHg (n = 349,190) or DBP ≥ 70 mmHg (n = 318,790).

**Supplementary Table S9:** Cox regression models for the associations of different systolic blood pressure (SBP) cut-offs (referenced to SBP 120 – 129 mmHg) with incident events among females (n = 203,162), stratified by age groups.

| Age group | SBP (mmHg) | Cardiovascular disease | | Chronic kidney disease | | Kidney failure | | All-cause death | |
| --- | --- | --- | --- | --- | --- | --- | --- | --- | --- |
|  |  | HR (95% CI) | P-value | HR (95% CI) | P-value | HR (95% CI) | P-value | HR (95% CI) | P-value |
| 18-44 years | <120 | 0.96 (0.72, 1.27) | 0.774 | 0.96 (0.79, 1.17) | 0.703 | 0.78 (0.53, 1.15) | 0.205 | 1.18 (0.90, 1.55) | 0.233 |
|  | 120-129 | Ref |  | Ref |  | Ref |  | Ref |  |
|  | 130-139 | 1.19 (0.89, 1.59) | 0.246 | 1.12 (0.91, 1.37) | 0.270 | 0.85 (0.56, 1.29) | 0.438 | 1.09 (0.81, 1.49) | 0.563 |
|  | 140-149 | 1.39 (1.03, 1.88) | 0.030 | 1.24 (1.00, 1.54) | 0.046 | 1.35 (0.91, 2.00) | 0.141 | 1.38 (1.01, 1.89) | 0.045 |
|  | 150-159 | 1.55 (1.10, 2.20) | 0.013 | 1.73 (1.37, 2.19) | <0.001 | 1.75 (1.12, 2.75) | 0.015 | 1.66 (1.15, 2.37) | 0.006 |
|  | 160-169 | 2.23 (1.45, 3.44) | <0.001 | 1.87 (1.34, 2.62) | <0.001 | 1.16 (0.62, 2.18) | 0.646 | 2.10 (1.31, 3.36) | 0.002 |
|  | 170-179 | 2.60 (1.35, 5.04) | 0.005 | 2.40 (1.52, 3.80) | <0.001 | 2.14 (0.94, 4.87) | 0.070 | 2.64 (1.39, 5.01) | 0.003 |
|  | ≥180 | 3.35 (1.89, 5.93) | <0.001 | 3.80 (2.50, 5.76) | <0.001 | 3.62 (1.87, 7.01) | <0.001 | 3.80 (2.16, 6.66) | <0.001 |
| 45-59 years | <120 | 0.96 (0.86, 1.08) | 0.514 | 1.00 (0.93, 1.07) | 0.977 | 1.03 (0.85, 1.24) | 0.777 | 1.10 (1.00, 1.21) | 0.053 |
|  | 120-129 | Ref |  | Ref |  | Ref |  | Ref |  |
|  | 130-139 | 1.10 (0.99, 1.22) | 0.085 | 1.04 (0.98, 1.11) | 0.168 | 1.03 (0.86, 1.23) | 0.753 | 0.97 (0.88, 1.07) | 0.556 |
|  | 140-149 | 1.20 (1.08, 1.34) | 0.001 | 1.12 (1.05, 1.19) | <0.001 | 1.24 (1.04, 1.47) | 0.016 | 1.10 (1.00, 1.22) | 0.059 |
|  | 150-159 | 1.31 (1.16, 1.48) | <0.001 | 1.28 (1.19, 1.37) | <0.001 | 1.44 (1.21, 1.73) | <0.001 | 1.15 (1.03, 1.28) | 0.014 |
|  | 160-169 | 1.38 (1.18, 1.61) | <0.001 | 1.38 (1.26, 1.51) | <0.001 | 1.84 (1.51, 2.25) | <0.001 | 1.35 (1.18, 1.54) | <0.001 |
|  | 170-179 | 1.81 (1.49, 2.19) | <0.001 | 1.61 (1.43, 1.82) | <0.001 | 2.12 (1.66, 2.71) | <0.001 | 1.62 (1.37, 1.92) | <0.001 |
|  | ≥180 | 1.94 (1.58, 2.39) | <0.001 | 2.07 (1.82, 2.35) | <0.001 | 2.86 (2.28, 3.59) | <0.001 | 1.94 (1.64, 2.30) | <0.001 |
| 60-74 years | <120 | 0.95 (0.86, 1.03) | 0.224 | 1.06 (1.01, 1.11) | 0.031 | 1.07 (0.93, 1.23) | 0.366 | 1.09 (1.02, 1.16) | 0.009 |
|  | 120-129 | Ref |  | Ref |  | Ref |  | Ref |  |
|  | 130-139 | 0.96 (0.89, 1.03) | 0.260 | 1.07 (1.03, 1.12) | 0.001 | 1.07 (0.95, 1.21) | 0.270 | 1.01 (0.96, 1.07) | 0.629 |
|  | 140-149 | 0.99 (0.91, 1.07) | 0.770 | 1.10 (1.05, 1.15) | <0.001 | 1.28 (1.14, 1.44) | <0.001 | 1.07 (1.01, 1.13) | 0.013 |
|  | 150-159 | 1.08 (0.99, 1.17) | 0.076 | 1.18 (1.12, 1.24) | <0.001 | 1.42 (1.26, 1.59) | <0.001 | 1.10 (1.04, 1.17) | 0.002 |
|  | 160-169 | 1.16 (1.05, 1.28) | 0.004 | 1.25 (1.18, 1.32) | <0.001 | 1.58 (1.39, 1.81) | <0.001 | 1.22 (1.14, 1.30) | <0.001 |
|  | 170-179 | 1.27 (1.12, 1.45) | <0.001 | 1.29 (1.19, 1.40) | <0.001 | 1.85 (1.58, 2.16) | <0.001 | 1.45 (1.33, 1.57) | <0.001 |
|  | ≥180 | 1.45 (1.28, 1.65) | <0.001 | 1.48 (1.36, 1.61) | <0.001 | 1.98 (1.71, 2.29) | <0.001 | 1.45 (1.33, 1.58) | <0.001 |
| ≥75 years | <120 | 1.20 (1.06, 1.36) | 0.003 | 1.03 (0.95, 1.12) | 0.444 | 1.18 (0.98, 1.41) | 0.082 | 1.05 (0.99, 1.12) | 0.125 |
|  | 120-129 | Ref |  | Ref |  | Ref |  | Ref |  |
|  | 130-139 | 1.11 (0.99, 1.23) | 0.063 | 1.04 (0.98, 1.12) | 0.205 | 1.38 (1.18, 1.61) | <0.001 | 0.97 (0.92, 1.03) | 0.300 |
|  | 140-149 | 1.08 (0.98, 1.21) | 0.133 | 1.05 (0.98, 1.12) | 0.148 | 1.41 (1.21, 1.63) | <0.001 | 0.98 (0.93, 1.03) | 0.432 |
|  | 150-159 | 1.09 (0.98, 1.22) | 0.128 | 1.07 (0.99, 1.15) | 0.082 | 1.59 (1.37, 1.85) | <0.001 | 1.02 (0.96, 1.07) | 0.573 |
|  | 160-169 | 1.23 (1.08, 1.40) | 0.001 | 1.16 (1.06, 1.27) | <0.001 | 1.52 (1.29, 1.81) | <0.001 | 1.04 (0.97, 1.11) | 0.274 |
|  | 170-179 | 1.35 (1.16, 1.58) | <0.001 | 1.25 (1.12, 1.39) | <0.001 | 1.62 (1.32, 1.98) | <0.001 | 1.10 (1.01, 1.19) | 0.021 |
|  | ≥180 | 1.45 (1.24, 1.70) | <0.001 | 1.23 (1.09, 1.38) | <0.001 | 1.84 (1.52, 2.22) | <0.001 | 1.18 (1.09, 1.28) | <0.001 |

The analysis is adjusted for calendar year at assessment, age, diabetes duration, BMI, smoking, HbA1c, LDL cholesterol, HDL cholesterol, log triglycerides, presence of albuminuria, history of CKD, history of CVD and use of blood pressure lowering medications.

**Supplementary Table S10:** Cox regression models for the associations of different diastolic blood pressure (DBP) cut-offs (referenced to DBP 70 – 79 mmHg) with incident events among females (n = 203,162), stratified by age groups.

| Age group | DBP (mmHg) | Cardiovascular disease | | Chronic kidney disease | | Kidney failure | | All-cause death | |
| --- | --- | --- | --- | --- | --- | --- | --- | --- | --- |
|  |  | HR (95% CI) | P-value | HR (95% CI) | P-value | HR (95% CI) | P-value | HR (95% CI) | P-value |
| 18-44 years | <60 | 0.74 (0.40, 1.38) | 0.348 | 0.45 (0.26, 0.77) | 0.004 | 0.38 (0.14, 1.05) | 0.063 | 1.40 (0.90, 2.19) | 0.135 |
|  | 60-69 | 0.90 (0.66, 1.22) | 0.501 | 0.82 (0.66, 1.01) | 0.066 | 0.67 (0.44, 1.02) | 0.063 | 0.94 (0.71, 1.25) | 0.684 |
|  | 70-79 | Ref |  | Ref |  | Ref |  | Ref |  |
|  | 80-89 | 1.24 (0.99, 1.56) | 0.059 | 1.18 (1.01, 1.38) | 0.039 | 1.24 (0.92, 1.66) | 0.157 | 0.99 (0.78, 1.24) | 0.901 |
|  | 90-99 | 1.35 (1.02, 1.80) | 0.037 | 1.19 (0.97, 1.45) | 0.096 | 1.19 (0.82, 1.73) | 0.355 | 1.27 (0.95, 1.69) | 0.102 |
|  | 100-109 | 1.39 (0.80, 2.43) | 0.245 | 1.52 (1.07, 2.17) | 0.020 | 0.47 (0.19, 1.18) | 0.106 | 0.81 (0.41, 1.61) | 0.556 |
|  | ≥110 | 4.36 (2.27, 8.39) | <0.001 | 1.99 (1.11, 3.58) | 0.022 | 1.44 (0.55, 3.78) | 0.455 | 3.00 (1.56, 5.79) | 0.001 |
| 45-59 years | <60 | 0.93 (0.77, 1.12) | 0.425 | 0.98 (0.88, 1.09) | 0.697 | 1.11 (0.84, 1.45) | 0.474 | 1.36 (1.18, 1.57) | <0.001 |
|  | 60-69 | 0.95 (0.87, 1.05) | 0.350 | 0.96 (0.90, 1.02) | 0.144 | 0.98 (0.84, 1.14) | 0.779 | 1.08 (0.99, 1.18) | 0.079 |
|  | 70-79 | Ref |  | Ref |  | Ref |  | Ref |  |
|  | 80-89 | 1.00 (0.92, 1.08) | 0.905 | 1.04 (0.99, 1.09) | 0.109 | 0.99 (0.88, 1.12) | 0.912 | 0.99 (0.92, 1.07) | 0.756 |
|  | 90-99 | 1.10 (0.98, 1.23) | 0.102 | 1.16 (1.09, 1.24) | <0.001 | 1.38 (1.18, 1.62) | <0.001 | 1.24 (1.13, 1.37) | <0.001 |
|  | 100-109 | 1.65 (1.31, 2.07) | <0.001 | 1.36 (1.18, 1.57) | <0.001 | 1.40 (1.05, 1.87) | 0.023 | 1.27 (1.03, 1.58) | 0.027 |
|  | ≥110 | 2.18 (1.38, 3.43) | <0.001 | 1.51 (1.10, 2.08) | 0.010 | 2.76 (1.65, 4.62) | <0.001 | 1.95 (1.31, 2.89) | <0.001 |
| 60-74 years | <60 | 1.15 (1.04, 1.26) | 0.006 | 1.14 (1.08, 1.20) | <0.001 | 1.06 (0.94, 1.20) | 0.352 | 1.22 (1.15, 1.30) | <0.001 |
|  | 60-69 | 1.05 (0.98, 1.11) | 0.152 | 1.01 (0.97, 1.04) | 0.655 | 1.05 (0.97, 1.14) | 0.256 | 1.08 (1.03, 1.13) | <0.001 |
|  | 70-79 | Ref |  | Ref |  | Ref |  | Ref |  |
|  | 80-89 | 1.05 (0.99, 1.12) | 0.089 | 1.01 (0.98, 1.05) | 0.411 | 0.97 (0.89, 1.05) | 0.469 | 1.05 (1.01, 1.10) | 0.017 |
|  | 90-99 | 1.18 (1.07, 1.30) | <0.001 | 1.13 (1.07, 1.20) | <0.001 | 1.08 (0.95, 1.22) | 0.225 | 1.12 (1.05, 1.20) | <0.001 |
|  | 100-109 | 1.45 (1.16, 1.81) | 0.001 | 1.44 (1.27, 1.65) | <0.001 | 1.25 (0.95, 1.64) | 0.108 | 1.28 (1.09, 1.50) | 0.003 |
|  | ≥110 | 1.84 (1.16, 2.92) | 0.010 | 2.13 (1.61, 2.83) | <0.001 | 1.58 (0.93, 2.68) | 0.088 | 1.97 (1.44, 2.69) | <0.001 |
| ≥75 years | <60 | 1.14 (1.03, 1.25) | 0.009 | 1.07 (1.01, 1.15) | 0.034 | 1.20 (1.06, 1.35) | 0.003 | 1.11 (1.06, 1.17) | <0.001 |
|  | 60-69 | 1.01 (0.94, 1.09) | 0.824 | 1.04 (0.99, 1.09) | 0.098 | 1.05 (0.95, 1.15) | 0.352 | 1.01 (0.97, 1.05) | 0.498 |
|  | 70-79 | Ref |  | Ref |  | Ref |  | Ref |  |
|  | 80-89 | 1.08 (0.99, 1.18) | 0.094 | 1.04 (0.98, 1.11) | 0.175 | 1.02 (0.90, 1.15) | 0.760 | 1.06 (1.01, 1.12) | 0.012 |
|  | 90-99 | 1.20 (1.03, 1.39) | 0.020 | 1.06 (0.95, 1.19) | 0.278 | 0.88 (0.71, 1.09) | 0.236 | 1.16 (1.07, 1.25) | <0.001 |
|  | 100-109 | 1.54 (1.11, 2.14) | 0.009 | 1.77 (1.36, 2.30) | <0.001 | 1.18 (0.78, 1.77) | 0.435 | 0.99 (0.81, 1.21) | 0.899 |
|  | ≥110 | 1.64 (0.82, 3.29) | 0.163 | 1.11 (0.50, 2.48) | 0.792 | 1.13 (0.42, 3.02) | 0.810 | 1.30 (0.86, 1.96) | 0.208 |

The analysis is adjusted for calendar year at assessment, age, diabetes duration, BMI, smoking, HbA1c, LDL cholesterol, HDL cholesterol, log triglycerides, presence of albuminuria, history of CKD, history of CVD and use of blood pressure lowering medications.

**Supplementary Table S11:** Cox regression models for the associations of different systolic blood pressure (SBP) cut-offs (referenced to SBP 120 – 129 mmHg) with incident events among males (n = 226,578), stratified by age groups.

| Age group | SBP (mmHg) | CVD | | CKD | | Kidney Failure | | All-cause mortality | |
| --- | --- | --- | --- | --- | --- | --- | --- | --- | --- |
|  |  | HR (95% CI) | P value | HR (95% CI) | P | HR (95% CI) | P value | HR (95% CI) | P |
| 18-44 years | <120 | 0.95 (0.80, 1.14) | 0.597 | 0.95 (0.83, 1.09) | 0.482 | 0.99 (0.77, 1.25) | 0.903 | 1.05 (0.87, 1.27) | 0.588 |
|  | 120-129 | Ref |  | Ref |  | Ref |  | Ref |  |
|  | 130-139 | 1.00 (0.84, 1.19) | 0.989 | 1.10 (0.96, 1.26) | 0.152 | 1.20 (0.94, 1.54) | 0.147 | 0.98 (0.80, 1.19) | 0.838 |
|  | 140-149 | 1.18 (0.98, 1.41) | 0.085 | 1.45 (1.26, 1.66) | <0.001 | 1.62 (1.26, 2.07) | <0.001 | 1.18 (0.96, 1.45) | 0.124 |
|  | 150-159 | 1.36 (1.09, 1.69) | 0.006 | 1.42 (1.20, 1.69) | <0.001 | 1.82 (1.37, 2.41) | <0.001 | 1.31 (1.02, 1.67) | 0.032 |
|  | 160-169 | 1.88 (1.44, 2.45) | <0.001 | 1.55 (1.23, 1.95) | <0.001 | 2.11 (1.51, 2.94) | <0.001 | 1.47 (1.08, 1.99) | 0.013 |
|  | 170-179 | 1.81 (1.13, 2.90) | 0.013 | 1.92 (1.36, 2.71) | <0.001 | 3.63 (2.22, 5.93) | <0.001 | 1.52 (0.86, 2.68) | 0.145 |
|  | ≥180 | 2.05 (1.20, 3.53) | 0.009 | 2.80 (1.93, 4.08) | <0.001 | 3.23 (1.86, 5.60) | <0.001 | 2.35 (1.45, 3.81) | <0.001 |
| 45-59 years | <120 | 0.99 (0.92, 1.07) | 0.847 | 0.99 (0.94, 1.04) | 0.608 | 1.01 (0.90, 1.15) | 0.820 | 1.13 (1.06, 1.21) | <0.001 |
|  | 120-129 | Ref |  | Ref |  | Ref |  | Ref |  |
|  | 130-139 | 1.06 (0.99, 1.14) | 0.096 | 1.06 (1.01, 1.11) | 0.024 | 1.03 (0.92, 1.17) | 0.579 | 1.01 (0.94, 1.08) | 0.799 |
|  | 140-149 | 1.16 (1.08, 1.25) | <0.001 | 1.21 (1.15, 1.27) | <0.001 | 1.42 (1.26, 1.59) | <0.001 | 1.15 (1.07, 1.23) | <0.001 |
|  | 150-159 | 1.23 (1.13, 1.34) | <0.001 | 1.38 (1.30, 1.46) | <0.001 | 1.68 (1.49, 1.91) | <0.001 | 1.27 (1.18, 1.37) | <0.001 |
|  | 160-169 | 1.33 (1.19, 1.49) | <0.001 | 1.51 (1.40, 1.63) | <0.001 | 1.74 (1.50, 2.01) | <0.001 | 1.46 (1.33, 1.61) | <0.001 |
|  | 170-179 | 1.56 (1.32, 1.84) | <0.001 | 1.70 (1.52, 1.90) | <0.001 | 2.48 (2.06, 2.97) | <0.001 | 1.58 (1.38, 1.81) | <0.001 |
|  | ≥180 | 1.99 (1.68, 2.35) | <0.001 | 2.01 (1.76, 2.29) | <0.001 | 2.60 (2.16, 3.12) | <0.001 | 2.14 (1.88, 2.45) | <0.001 |
| 60-74 years | <120 | 1.03 (0.96, 1.11) | 0.435 | 1.02 (0.98, 1.07) | 0.264 | 1.02 (0.90, 1.16) | 0.720 | 1.06 (1.01, 1.11) | 0.018 |
|  | 120-129 | Ref |  | Ref |  | Ref |  | Ref |  |
|  | 130-139 | 1.00 (0.94, 1.07) | 0.914 | 1.03 (0.99, 1.07) | 0.099 | 1.07 (0.96, 1.19) | 0.227 | 0.96 (0.92, 1.00) | 0.074 |
|  | 140-149 | 1.10 (1.03, 1.17) | 0.006 | 1.10 (1.06, 1.15) | <0.001 | 1.25 (1.13, 1.40) | <0.001 | 1.02 (0.97, 1.06) | 0.462 |
|  | 150-159 | 1.17 (1.09, 1.26) | <0.001 | 1.16 (1.11, 1.21) | <0.001 | 1.42 (1.27, 1.59) | <0.001 | 1.06 (1.01, 1.11) | 0.020 |
|  | 160-169 | 1.23 (1.13, 1.35) | <0.001 | 1.19 (1.13, 1.26) | <0.001 | 1.53 (1.35, 1.73) | <0.001 | 1.13 (1.07, 1.19) | <0.001 |
|  | 170-179 | 1.29 (1.14, 1.46) | <0.001 | 1.39 (1.28, 1.50) | <0.001 | 1.81 (1.55, 2.11) | <0.001 | 1.26 (1.16, 1.36) | <0.001 |
|  | ≥180 | 1.50 (1.32, 1.72) | <0.001 | 1.49 (1.36, 1.62) | <0.001 | 2.42 (2.09, 2.81) | <0.001 | 1.37 (1.26, 1.49) | <0.001 |
| ≥75 years | <120 | 1.08 (0.94, 1.23) | 0.279 | 1.07 (0.98, 1.16) | 0.130 | 1.10 (0.89, 1.36) | 0.379 | 1.12 (1.05, 1.19) | <0.001 |
|  | 120-129 | Ref |  | Ref |  | Ref |  | Ref |  |
|  | 130-139 | 1.02 (0.91, 1.15) | 0.745 | 0.97 (0.90, 1.05) | 0.443 | 1.17 (0.97, 1.41) | 0.103 | 0.94 (0.89, 0.99) | 0.024 |
|  | 140-149 | 1.06 (0.94, 1.19) | 0.341 | 1.06 (0.98, 1.14) | 0.145 | 1.32 (1.10, 1.58) | 0.003 | 0.96 (0.91, 1.01) | 0.108 |
|  | 150-159 | 1.06 (0.93, 1.20) | 0.380 | 1.14 (1.05, 1.23) | 0.002 | 1.42 (1.18, 1.72) | <0.001 | 0.98 (0.92, 1.03) | 0.422 |
|  | 160-169 | 1.29 (1.12, 1.49) | <0.001 | 1.25 (1.13, 1.38) | <0.001 | 1.48 (1.20, 1.84) | <0.001 | 1.06 (0.98, 1.13) | 0.131 |
|  | 170-179 | 1.25 (1.03, 1.51) | 0.023 | 1.32 (1.16, 1.51) | <0.001 | 1.70 (1.32, 2.17) | <0.001 | 1.02 (0.93, 1.12) | 0.634 |
|  | ≥180 | 1.17 (0.94, 1.46) | 0.160 | 1.38 (1.18, 1.62) | <0.001 | 2.24 (1.74, 2.89) | <0.001 | 1.15 (1.04, 1.28) | 0.006 |

The analysis is adjusted for calendar year at assessment, age, diabetes duration, BMI, smoking, HbA1c, LDL cholesterol, HDL cholesterol, log triglycerides, presence of albuminuria, history of CKD, history of CVD and use of blood pressure lowering medications.

**Supplementary Table S12:** Cox regression models for the associations of different diastolic blood pressure (DBP) cut-offs (referenced to DBP 70 – 79 mmHg) with incident events among males (n = 226,578), stratified by age groups.

| Age group | DBP (mmHg) | Cardiovascular disease | | Chronic kidney disease | | Kidney failure | | All-cause death | |
| --- | --- | --- | --- | --- | --- | --- | --- | --- | --- |
|  |  | HR (95% CI) | P-value | HR (95% CI) | P-value | HR (95% CI) | P-value | HR (95% CI) | P-value |
| 18-44 years | <60 | 1.16 (0.73, 1.85) | 0.530 | 0.68 (0.44, 1.05) | 0.085 | 0.57 (0.27, 1.21) | 0.142 | 1.14 (0.73, 1.78) | 0.574 |
|  | 60-69 | 0.91 (0.74, 1.13) | 0.408 | 0.91 (0.77, 1.07) | 0.258 | 0.89 (0.68, 1.18) | 0.419 | 0.99 (0.80, 1.24) | 0.946 |
|  | 70-79 | Ref |  | Ref |  | Ref |  | Ref |  |
|  | 80-89 | 1.09 (0.94, 1.25) | 0.255 | 1.13 (1.01, 1.26) | 0.040 | 1.11 (0.92, 1.35) | 0.272 | 1.13 (0.97, 1.33) | 0.119 |
|  | 90-99 | 1.20 (1.01, 1.43) | 0.033 | 1.35 (1.18, 1.54) | <0.001 | 1.48 (1.19, 1.84) | <0.001 | 1.23 (1.02, 1.49) | 0.029 |
|  | 100-109 | 1.58 (1.20, 2.08) | 0.001 | 1.85 (1.51, 2.26) | <0.001 | 1.76 (1.24, 2.48) | 0.001 | 1.34 (0.96, 1.86) | 0.084 |
|  | ≥110 | 2.06 (1.35, 3.13) | <0.001 | 1.69 (1.19, 2.40) | 0.003 | 2.15 (1.32, 3.48) | 0.002 | 1.68 (1.02, 2.75) | 0.041 |
| 45-59 years | <60 | 1.23 (1.03, 1.47) | 0.019 | 1.08 (0.95, 1.23) | 0.244 | 1.21 (0.95, 1.55) | 0.129 | 1.50 (1.31, 1.71) | <0.001 |
|  | 60-69 | 0.99 (0.91, 1.07) | 0.767 | 0.99 (0.93, 1.04) | 0.671 | 0.96 (0.85, 1.08) | 0.513 | 1.10 (1.02, 1.17) | 0.008 |
|  | 70-79 | Ref |  | Ref |  | Ref |  | Ref |  |
|  | 80-89 | 1.00 (0.95, 1.06) | 0.886 | 1.09 (1.05, 1.13) | <0.001 | 1.02 (0.94, 1.12) | 0.600 | 0.96 (0.91, 1.01) | 0.145 |
|  | 90-99 | 1.09 (1.01, 1.17) | 0.019 | 1.25 (1.19, 1.31) | <0.001 | 1.15 (1.04, 1.28) | 0.006 | 1.09 (1.02, 1.16) | 0.010 |
|  | 100-109 | 1.35 (1.19, 1.53) | <0.001 | 1.49 (1.36, 1.63) | <0.001 | 1.19 (1.00, 1.41) | 0.044 | 1.19 (1.06, 1.34) | 0.004 |
|  | ≥110 | 1.99 (1.55, 2.56) | <0.001 | 1.73 (1.44, 2.08) | <0.001 | 1.70 (1.25, 2.30) | <0.001 | 1.83 (1.48, 2.27) | <0.001 |
| 60-74 years | <60 | 1.11 (0.99, 1.24) | 0.064 | 1.11 (1.04, 1.18) | 0.002 | 1.14 (0.98, 1.33) | 0.079 | 1.26 (1.19, 1.34) | <0.001 |
|  | 60-69 | 1.01 (0.96, 1.07) | 0.673 | 1.05 (1.01, 1.08) | 0.010 | 1.15 (1.06, 1.25) | <0.001 | 1.10 (1.06, 1.14) | <0.001 |
|  | 70-79 | Ref |  | Ref |  | Ref |  | Ref |  |
|  | 80-89 | 0.98 (0.93, 1.03) | 0.435 | 1.02 (0.99, 1.05) | 0.301 | 1.11 (1.03, 1.19) | 0.008 | 0.99 (0.96, 1.03) | 0.639 |
|  | 90-99 | 1.09 (1.02, 1.17) | 0.015 | 1.11 (1.06, 1.16) | <0.001 | 1.12 (1.01, 1.24) | 0.037 | 1.07 (1.02, 1.12) | 0.008 |
|  | 100-109 | 1.27 (1.08, 1.48) | 0.004 | 1.27 (1.15, 1.40) | <0.001 | 1.37 (1.12, 1.68) | 0.002 | 1.32 (1.19, 1.47) | <0.001 |
|  | ≥110 | 1.88 (1.35, 2.61) | <0.001 | 1.93 (1.54, 2.41) | <0.001 | 1.49 (0.95, 2.35) | 0.082 | 1.61 (1.27, 2.04) | <0.001 |
| ≥75 years | <60 | 1.12 (0.99, 1.26) | 0.077 | 1.16 (1.06, 1.25) | <0.001 | 1.22 (1.04, 1.44) | 0.015 | 1.25 (1.19, 1.32) | <0.001 |
|  | 60-69 | 0.98 (0.90, 1.06) | 0.582 | 1.04 (0.98, 1.10) | 0.153 | 1.08 (0.96, 1.23) | 0.191 | 1.09 (1.05, 1.14) | <0.001 |
|  | 70-79 | Ref |  | Ref |  | Ref |  | Ref |  |
|  | 80-89 | 1.01 (0.92, 1.12) | 0.779 | 1.03 (0.97, 1.10) | 0.328 | 0.98 (0.85, 1.13) | 0.821 | 1.04 (1.00, 1.09) | 0.078 |
|  | 90-99 | 1.13 (0.96, 1.32) | 0.140 | 1.22 (1.09, 1.36) | <0.001 | 1.02 (0.80, 1.29) | 0.886 | 1.07 (0.98, 1.16) | 0.120 |
|  | 100-109 | 1.16 (0.77, 1.76) | 0.477 | 1.12 (0.81, 1.55) | 0.478 | 1.25 (0.75, 2.09) | 0.395 | 1.29 (1.06, 1.57) | 0.012 |
|  | ≥110 | 1.38 (0.52, 3.68) | 0.524 | 0.88 (0.46, 1.69) | 0.703 | 1.08 (0.27, 4.35) | 0.908 | 1.64 (1.09, 2.47) | 0.018 |

The analysis is adjusted for calendar year at assessment, age, diabetes duration, BMI, smoking, HbA1c, LDL cholesterol, HDL cholesterol, log triglycerides, presence of albuminuria, history of CKD, history of CVD and use of blood pressure lowering medications.

**Supplementary Table S13**: Cox regression models for the associations of different systolic blood pressure (SBP) cut-offs (referenced to SBP 120 – 129 mmHg) with incident events among individuals who were using blood pressure lowering medications at baseline (n = 281,985), stratified by age groups.

| Age group | SBP (mmHg) | Cardiovascular disease | | Chronic Kidney Disease | | Kidney failure | | All-cause death | |
| --- | --- | --- | --- | --- | --- | --- | --- | --- | --- |
|  |  | HR (95% CI) | P -value | HR (95% CI) | P-value | HR (95% CI) | P-value | HR (95% CI) | P-value |
| 18-44 years | <120 | 0.81 (0.63, 1.05) | 0.107 | 0.98 (0.82, 1.17) | 0.827 | 0.81 (0.59, 1.11) | 0.180 | 1.20 (0.92, 1.57) | 0.173 |
|  | 120-129 | Ref |  | Ref |  | Ref |  | Ref |  |
|  | 130-139 | 0.93 (0.75, 1.15) | 0.502 | 0.99 (0.84, 1.16) | 0.888 | 1.07 (0.80, 1.44) | 0.625 | 1.04 (0.81, 1.34) | 0.754 |
|  | 140-149 | 1.09 (0.88, 1.35) | 0.428 | 1.21 (1.03, 1.42) | 0.019 | 1.40 (1.05, 1.85) | 0.021 | 1.17 (0.90, 1.51) | 0.233 |
|  | 150-159 | 1.18 (0.92, 1.51) | 0.184 | 1.30 (1.09, 1.56) | 0.005 | 1.49 (1.10, 2.03) | 0.011 | 1.35 (1.02, 1.79) | 0.037 |
|  | 160-169 | 1.80 (1.35, 2.38) | <0.001 | 1.32 (1.04, 1.69) | 0.022 | 1.27 (0.87, 1.85) | 0.220 | 1.76 (1.28, 2.43) | <0.001 |
|  | 170-179 | 1.42 (0.87, 2.31) | 0.164 | 1.86 (1.35, 2.57) | <0.001 | 2.38 (1.44, 3.94) | <0.001 | 2.02 (1.25, 3.27) | 0.004 |
|  | ≥180 | 2.39 (1.55, 3.69) | <0.001 | 2.92 (2.14, 3.99) | <0.001 | 3.05 (1.93, 4.83) | <0.001 | 2.89 (1.91, 4.38) | <0.001 |
| 45-59 years | <120 | 1.05 (0.96, 1.14) | 0.289 | 1.07 (1.02, 1.13) | 0.011 | 1.04 (0.91, 1.18) | 0.578 | 1.21 (1.12, 1.30) | <0.001 |
|  | 120-129 | Ref |  | Ref |  | Ref |  | Ref |  |
|  | 130-139 | 1.05 (0.97, 1.14) | 0.193 | 1.04 (0.99, 1.09) | 0.101 | 1.05 (0.94, 1.18) | 0.379 | 1.00 (0.93, 1.07) | 0.957 |
|  | 140-149 | 1.16 (1.07, 1.26) | <0.001 | 1.15 (1.09, 1.20) | <0.001 | 1.36 (1.21, 1.52) | <0.001 | 1.15 (1.08, 1.24) | <0.001 |
|  | 150-159 | 1.25 (1.14, 1.36) | <0.001 | 1.32 (1.25, 1.39) | <0.001 | 1.55 (1.38, 1.74) | <0.001 | 1.25 (1.16, 1.35) | <0.001 |
|  | 160-169 | 1.38 (1.24, 1.54) | <0.001 | 1.41 (1.32, 1.51) | <0.001 | 1.69 (1.48, 1.93) | <0.001 | 1.43 (1.30, 1.56) | <0.001 |
|  | 170-179 | 1.58 (1.37, 1.83) | <0.001 | 1.58 (1.43, 1.73) | <0.001 | 2.25 (1.91, 2.64) | <0.001 | 1.52 (1.35, 1.72) | <0.001 |
|  | ≥180 | 1.95 (1.68, 2.25) | <0.001 | 2.00 (1.81, 2.22) | <0.001 | 2.66 (2.28, 3.12) | <0.001 | 2.11 (1.88, 2.37) | <0.001 |
| 60-74 years | <120 | 1.00 (0.94, 1.08) | 0.924 | 1.07 (1.03, 1.11) | <0.001 | 1.07 (0.97, 1.18) | 0.194 | 1.09 (1.04, 1.13) | <0.001 |
|  | 120-129 | Ref |  | Ref |  | Ref |  | Ref |  |
|  | 130-139 | 0.96 (0.90, 1.02) | 0.151 | 1.04 (1.01, 1.07) | 0.018 | 1.06 (0.97, 1.15) | 0.225 | 0.98 (0.94, 1.02) | 0.265 |
|  | 140-149 | 1.01 (0.95, 1.07) | 0.718 | 1.08 (1.05, 1.12) | <0.001 | 1.24 (1.14, 1.35) | <0.001 | 1.03 (0.99, 1.07) | 0.116 |
|  | 150-159 | 1.10 (1.03, 1.17) | 0.004 | 1.15 (1.11, 1.19) | <0.001 | 1.40 (1.29, 1.53) | <0.001 | 1.07 (1.03, 1.11) | 0.002 |
|  | 160-169 | 1.16 (1.08, 1.25) | <0.001 | 1.20 (1.15, 1.26) | <0.001 | 1.52 (1.38, 1.67) | <0.001 | 1.16 (1.11, 1.22) | <0.001 |
|  | 170-179 | 1.25 (1.13, 1.38) | <0.001 | 1.31 (1.23, 1.39) | <0.001 | 1.75 (1.56, 1.97) | <0.001 | 1.36 (1.28, 1.44) | <0.001 |
|  | ≥180 | 1.46 (1.32, 1.61) | <0.001 | 1.47 (1.37, 1.57) | <0.001 | 2.10 (1.88, 2.35) | <0.001 | 1.39 (1.31, 1.48) | <0.001 |
| ≥75 years | <120 | 1.17 (1.06, 1.29) | 0.002 | 1.08 (1.01, 1.16) | 0.017 | 1.14 (0.99, 1.32) | 0.075 | 1.09 (1.04, 1.14) | <0.001 |
|  | 120-129 | Ref |  | Ref |  | Ref |  | Ref |  |
|  | 130-139 | 1.07 (0.99, 1.17) | 0.100 | 1.02 (0.96, 1.07) | 0.590 | 1.27 (1.13, 1.44) | <0.001 | 0.95 (0.92, 0.99) | 0.025 |
|  | 140-149 | 1.08 (0.99, 1.18) | 0.069 | 1.05 (1.00, 1.11) | 0.057 | 1.36 (1.21, 1.53) | <0.001 | 0.97 (0.93, 1.01) | 0.108 |
|  | 150-159 | 1.06 (0.97, 1.16) | 0.181 | 1.11 (1.05, 1.17) | <0.001 | 1.50 (1.33, 1.70) | <0.001 | 1.00 (0.95, 1.04) | 0.838 |
|  | 160-169 | 1.27 (1.15, 1.41) | <0.001 | 1.22 (1.13, 1.31) | <0.001 | 1.50 (1.31, 1.72) | <0.001 | 1.06 (1.01, 1.11) | 0.030 |
|  | 170-179 | 1.32 (1.16, 1.50) | <0.001 | 1.27 (1.16, 1.39) | <0.001 | 1.64 (1.39, 1.92) | <0.001 | 1.06 (0.99, 1.13) | 0.074 |
|  | ≥180 | 1.33 (1.16, 1.52) | <0.001 | 1.29 (1.17, 1.42) | <0.001 | 1.95 (1.66, 2.28) | <0.001 | 1.18 (1.10, 1.26) | <0.001 |

The analysis is adjusted for calendar year at assessment, age, sex, diabetes duration, BMI, smoking, HbA1c, LDL cholesterol, HDL cholesterol, log triglycerides, presence of albuminuria, history of CKD and history of CVD.

**Supplementary Table S14**: Cox regression models for the associations of different diastolic blood pressure (DBP) cut-offs (referenced to DBP 70 – 79 mmHg) with incident events among individuals who were using blood pressure lowering medications at baseline (n = 281,985), stratified by age groups.

| Age group | DBP (mmHg) | Cardiovascular Disease | | Chronic Kidney Disease | | Kidney failure | | All-cause death | |
| --- | --- | --- | --- | --- | --- | --- | --- | --- | --- |
|  |  | HR (95% CI) | P-value | HR (95% CI) | P-value | HR (95% CI) | P-value | HR (95% CI) | P-value |
| 18-44 years | <60 | 0.99 (0.49, 2.02) | 0.982 | 0.81 (0.47, 1.38) | 0.432 | 0.26 (0.06, 1.04) | 0.057 | 1.47 (0.82, 2.65) | 0.199 |
|  | 60-69 | 0.93 (0.68, 1.26) | 0.636 | 1.07 (0.88, 1.32) | 0.490 | 0.87 (0.61, 1.24) | 0.440 | 1.00 (0.74, 1.36) | 0.990 |
|  | 70-79 | Ref |  | Ref |  | Ref |  | Ref |  |
|  | 80-89 | 1.15 (0.96, 1.37) | 0.138 | 1.11 (0.98, 1.27) | 0.112 | 1.33 (1.06, 1.66) | 0.012 | 1.11 (0.91, 1.36) | 0.282 |
|  | 90-99 | 1.24 (1.02, 1.52) | 0.033 | 1.21 (1.04, 1.40) | 0.012 | 1.34 (1.05, 1.73) | 0.021 | 1.22 (0.98, 1.52) | 0.069 |
|  | 100-109 | 1.62 (1.20, 2.17) | 0.002 | 1.72 (1.39, 2.11) | <0.001 | 1.17 (0.78, 1.74) | 0.457 | 1.34 (0.95, 1.89) | 0.092 |
|  | ≥110 | 2.27 (1.48, 3.48) | <0.001 | 1.79 (1.27, 2.53) | <0.001 | 2.10 (1.29, 3.43) | 0.003 | 2.16 (1.38, 3.39) | <0.001 |
| 45-59 years | <60 | 1.23 (1.02, 1.47) | 0.027 | 1.20 (1.07, 1.33) | 0.001 | 1.01 (0.80, 1.27) | 0.962 | 1.50 (1.32, 1.70) | <0.001 |
|  | 60-69 | 1.08 (0.99, 1.17) | 0.072 | 1.04 (0.99, 1.09) | 0.150 | 0.97 (0.87, 1.09) | 0.629 | 1.11 (1.03, 1.18) | 0.004 |
|  | 70-79 | Ref |  | Ref |  | Ref |  | Ref |  |
|  | 80-89 | 0.98 (0.92, 1.04) | 0.455 | 1.04 (1.00, 1.07) | 0.064 | 0.97 (0.89, 1.05) | 0.452 | 0.94 (0.89, 0.99) | 0.013 |
|  | 90-99 | 1.09 (1.01, 1.17) | 0.023 | 1.19 (1.13, 1.24) | <0.001 | 1.13 (1.02, 1.24) | 0.015 | 1.07 (1.01, 1.14) | 0.033 |
|  | 100-109 | 1.30 (1.14, 1.49) | <0.001 | 1.37 (1.26, 1.50) | <0.001 | 1.13 (0.97, 1.33) | 0.126 | 1.17 (1.05, 1.32) | 0.007 |
|  | ≥110 | 2.04 (1.60, 2.62) | <0.001 | 1.63 (1.36, 1.95) | <0.001 | 1.81 (1.36, 2.42) | <0.001 | 1.91 (1.55, 2.35) | <0.001 |
| 60-74 years | <60 | 1.16 (1.07, 1.26) | <0.001 | 1.16 (1.11, 1.22) | <0.001 | 1.12 (1.01, 1.23) | 0.032 | 1.27 (1.21, 1.34) | <0.001 |
|  | 60-69 | 1.02 (0.97, 1.07) | 0.443 | 1.03 (1.01, 1.06) | 0.017 | 1.13 (1.06, 1.20) | <0.001 | 1.10 (1.06, 1.13) | <0.001 |
|  | 70-79 | Ref |  | Ref |  | Ref |  | Ref |  |
|  | 80-89 | 1.01 (0.96, 1.05) | 0.755 | 1.00 (0.98, 1.03) | 0.800 | 1.04 (0.98, 1.11) | 0.158 | 1.01 (0.98, 1.04) | 0.494 |
|  | 90-99 | 1.11 (1.04, 1.19) | 0.001 | 1.10 (1.06, 1.15) | <0.001 | 1.08 (0.99, 1.18) | 0.077 | 1.09 (1.04, 1.13) | <0.001 |
|  | 100-109 | 1.27 (1.09, 1.47) | 0.002 | 1.29 (1.18, 1.40) | <0.001 | 1.38 (1.16, 1.64) | <0.001 | 1.28 (1.16, 1.41) | <0.001 |
|  | ≥110 | 1.89 (1.40, 2.55) | <0.001 | 1.98 (1.64, 2.40) | <0.001 | 1.49 (1.02, 2.16) | 0.037 | 1.81 (1.48, 2.21) | <0.001 |
| ≥75 years | <60 | 1.13 (1.04, 1.23) | 0.003 | 1.13 (1.07, 1.19) | <0.001 | 1.24 (1.13, 1.37) | <0.001 | 1.18 (1.14, 1.23) | <0.001 |
|  | 60-69 | 1.00 (0.94, 1.06) | 0.970 | 1.06 (1.02, 1.10) | 0.004 | 1.08 (1.00, 1.17) | 0.051 | 1.05 (1.02, 1.08) | 0.002 |
|  | 70-79 | Ref |  | Ref |  | Ref |  | Ref |  |
|  | 80-89 | 1.07 (0.99, 1.15) | 0.075 | 1.05 (1.00, 1.10) | 0.067 | 1.02 (0.92, 1.12) | 0.740 | 1.06 (1.03, 1.10) | <0.001 |
|  | 90-99 | 1.14 (1.01, 1.28) | 0.034 | 1.13 (1.04, 1.23) | 0.004 | 0.96 (0.82, 1.13) | 0.638 | 1.11 (1.04, 1.18) | 0.001 |
|  | 100-109 | 1.36 (1.04, 1.79) | 0.027 | 1.45 (1.17, 1.80) | <0.001 | 1.11 (0.79, 1.56) | 0.540 | 1.09 (0.94, 1.26) | 0.276 |
|  | ≥110 | 1.64 (0.90, 2.96) | 0.104 | 0.99 (0.58, 1.67) | 0.961 | 1.25 (0.56, 2.78) | 0.591 | 1.54 (1.14, 2.08) | 0.005 |

The analysis is adjusted for calendar year at assessment, age, sex, diabetes duration, BMI, smoking, HbA1c, LDL cholesterol, HDL cholesterol, log triglycerides, presence of albuminuria, history of CKD and history of CVD.

**Supplementary Table S15**: Cox regression models for the associations of different systolic blood pressure (SBP) cut-offs (referenced to SBP 120 – 129 mmHg) with incident events among individuals who were **not** using blood pressure lowering medications at baseline (n = 147,755), stratified by age groups.

| Age group | SBP (mmHg) | Cardiovascular disease | | Chronic Kidney Disease | | Kidney failure | | All-cause death | |
| --- | --- | --- | --- | --- | --- | --- | --- | --- | --- |
|  |  | HR (95% CI) | P -value | HR (95% CI) | P-value | HR (95% CI) | P-value | HR (95% CI) | P-value |
| 18-44 years | <120 | 1.07 (0.89, 1.29) | 0.478 | 0.97 (0.84, 1.13) | 0.728 | 1.09 (0.83, 1.43) | 0.540 | 1.02 (0.84, 1.24) | 0.825 |
|  | 120-129 | Ref |  | Ref |  | Ref |  | Ref |  |
|  | 130-139 | 1.15 (0.94, 1.42) | 0.183 | 1.26 (1.07, 1.48) | 0.005 | 1.13 (0.83, 1.55) | 0.431 | 0.99 (0.79, 1.24) | 0.929 |
|  | 140-149 | 1.36 (1.08, 1.71) | 0.009 | 1.64 (1.38, 1.95) | <0.001 | 1.85 (1.35, 2.54) | <0.001 | 1.38 (1.08, 1.74) | 0.009 |
|  | 150-159 | 1.62 (1.22, 2.14) | <0.001 | 1.88 (1.52, 2.34) | <0.001 | 2.35 (1.59, 3.46) | <0.001 | 1.54 (1.14, 2.09) | 0.005 |
|  | 160-169 | 1.98 (1.34, 2.93) | <0.001 | 2.41 (1.78, 3.26) | <0.001 | 3.76 (2.32, 6.07) | <0.001 | 1.32 (0.81, 2.15) | 0.265 |
|  | 170-179 | 3.82 (2.07, 7.04) | <0.001 | 2.98 (1.71, 5.20) | <0.001 | 6.39 (2.94, 13.89) | <0.001 | 1.38 (0.51, 3.73) | 0.522 |
|  | ≥180 | 1.90 (0.70, 5.14) | 0.206 | 3.38 (1.73, 6.60) | <0.001 | 4.11 (1.28, 13.16) | 0.017 | 2.07 (0.76, 5.61) | 0.152 |
| 45-59 years | <120 | 0.94 (0.86, 1.02) | 0.158 | 0.92 (0.86, 0.98) | 0.006 | 0.96 (0.81, 1.15) | 0.684 | 1.03 (0.95, 1.11) | 0.529 |
|  | 120-129 | Ref |  | Ref |  | Ref |  | Ref |  |
|  | 130-139 | 1.10 (1.00, 1.20) | 0.042 | 1.08 (1.01, 1.15) | 0.027 | 0.91 (0.75, 1.10) | 0.324 | 0.99 (0.90, 1.08) | 0.806 |
|  | 140-149 | 1.20 (1.09, 1.33) | <0.001 | 1.24 (1.16, 1.33) | <0.001 | 1.27 (1.04, 1.55) | 0.018 | 1.10 (1.00, 1.21) | 0.060 |
|  | 150-159 | 1.29 (1.15, 1.46) | <0.001 | 1.42 (1.30, 1.54) | <0.001 | 1.73 (1.41, 2.14) | <0.001 | 1.18 (1.05, 1.33) | 0.006 |
|  | 160-169 | 1.22 (1.01, 1.46) | 0.036 | 1.63 (1.45, 1.83) | <0.001 | 2.23 (1.73, 2.89) | <0.001 | 1.39 (1.18, 1.64) | <0.001 |
|  | 170-179 | 2.02 (1.55, 2.62) | <0.001 | 2.16 (1.80, 2.59) | <0.001 | 3.03 (2.13, 4.33) | <0.001 | 2.12 (1.69, 2.67) | <0.001 |
|  | ≥180 | 2.10 (1.54, 2.87) | <0.001 | 2.60 (2.09, 3.22) | <0.001 | 2.72 (1.89, 3.91) | <0.001 | 2.06 (1.58, 2.69) | <0.001 |
| 60-74 years | <120 | 1.00 (0.90, 1.10) | 0.953 | 0.98 (0.92, 1.04) | 0.512 | 0.92 (0.73, 1.16) | 0.490 | 1.02 (0.95, 1.10) | 0.588 |
|  | 120-129 | Ref |  | Ref |  | Ref |  | Ref |  |
|  | 130-139 | 1.04 (0.95, 1.14) | 0.422 | 1.06 (1.00, 1.13) | 0.034 | 1.09 (0.88, 1.35) | 0.413 | 0.97 (0.91, 1.05) | 0.460 |
|  | 140-149 | 1.15 (1.05, 1.27) | 0.004 | 1.16 (1.09, 1.24) | <0.001 | 1.33 (1.08, 1.64) | 0.008 | 1.04 (0.97, 1.12) | 0.259 |
|  | 150-159 | 1.23 (1.10, 1.37) | <0.001 | 1.26 (1.17, 1.35) | <0.001 | 1.52 (1.21, 1.90) | <0.001 | 1.08 (1.00, 1.18) | 0.059 |
|  | 160-169 | 1.33 (1.16, 1.54) | <0.001 | 1.29 (1.17, 1.41) | <0.001 | 1.79 (1.39, 2.30) | <0.001 | 1.17 (1.05, 1.30) | 0.004 |
|  | 170-179 | 1.41 (1.15, 1.73) | <0.001 | 1.49 (1.31, 1.69) | <0.001 | 2.25 (1.66, 3.06) | <0.001 | 1.19 (1.03, 1.38) | 0.020 |
|  | ≥180 | 1.54 (1.21, 1.96) | <0.001 | 1.60 (1.36, 1.87) | <0.001 | 2.74 (2.02, 3.72) | <0.001 | 1.57 (1.33, 1.84) | <0.001 |
| ≥75 years | <120 | 1.04 (0.84, 1.29) | 0.745 | 0.90 (0.78, 1.04) | 0.165 | 1.24 (0.75, 2.04) | 0.409 | 1.06 (0.95, 1.18) | 0.306 |
|  | 120-129 | Ref |  | Ref |  | Ref |  | Ref |  |
|  | 130-139 | 1.04 (0.86, 1.26) | 0.684 | 1.01 (0.89, 1.14) | 0.914 | 1.45 (0.94, 2.25) | 0.093 | 0.96 (0.86, 1.06) | 0.390 |
|  | 140-149 | 1.01 (0.83, 1.23) | 0.913 | 1.07 (0.94, 1.21) | 0.311 | 1.49 (0.96, 2.31) | 0.075 | 0.95 (0.85, 1.06) | 0.333 |
|  | 150-159 | 1.19 (0.96, 1.47) | 0.108 | 1.04 (0.90, 1.20) | 0.599 | 1.76 (1.13, 2.76) | 0.012 | 0.99 (0.88, 1.11) | 0.905 |
|  | 160-169 | 1.12 (0.86, 1.48) | 0.396 | 1.10 (0.92, 1.32) | 0.283 | 1.57 (0.93, 2.67) | 0.094 | 0.91 (0.79, 1.06) | 0.236 |
|  | 170-179 | 1.30 (0.91, 1.86) | 0.156 | 1.33 (1.04, 1.71) | 0.025 | 1.72 (0.89, 3.32) | 0.106 | 1.11 (0.90, 1.35) | 0.325 |
|  | ≥180 | 1.58 (1.09, 2.29) | 0.016 | 1.22 (0.88, 1.71) | 0.232 | 1.91 (1.04, 3.51) | 0.038 | 1.20 (0.96, 1.48) | 0.104 |

The analysis is adjusted for calendar year at assessment, age, sex, diabetes duration, BMI, smoking, HbA1c, LDL cholesterol, HDL cholesterol, log triglycerides, presence of albuminuria, history of CKD and history of CVD.

**Supplementary Table S16**: Cox regression models for the associations of different diastolic blood pressure (DBP) cut-offs (referenced to DBP 70 – 79 mmHg) with incident events among individuals who were **not** using blood pressure lowering medications at baseline (n = 147,755), stratified by age groups.

| Age group | DBP (mmHg) | Cardiovascular Disease | | Chronic Kidney Disease | | Kidney failure | | All-cause death | |
| --- | --- | --- | --- | --- | --- | --- | --- | --- | --- |
|  |  | HR (95% CI) | P-value | HR (95% CI) | P-value | HR (95% CI) | P-value | HR (95% CI) | P-value |
| 18-44 years | <60 | 1.01 (0.65, 1.56) | 0.979 | 0.47 (0.30, 0.73) | <0.001 | 0.73 (0.37, 1.44) | 0.359 | 1.21 (0.83, 1.76) | 0.315 |
|  | 60-69 | 0.91 (0.74, 1.13) | 0.411 | 0.78 (0.66, 0.93) | 0.005 | 0.78 (0.57, 1.06) | 0.110 | 0.97 (0.78, 1.19) | 0.750 |
|  | 70-79 | Ref |  | Ref |  | Ref |  | Ref |  |
|  | 80-89 | 1.12 (0.95, 1.32) | 0.178 | 1.17 (1.03, 1.32) | 0.017 | 0.96 (0.76, 1.22) | 0.749 | 1.09 (0.91, 1.29) | 0.360 |
|  | 90-99 | 1.25 (1.00, 1.56) | 0.054 | 1.51 (1.27, 1.79) | <0.001 | 1.65 (1.23, 2.21) | <0.001 | 1.36 (1.08, 1.72) | 0.010 |
|  | 100-109 | 1.34 (0.84, 2.15) | 0.219 | 1.76 (1.24, 2.49) | 0.001 | 2.24 (1.32, 3.80) | 0.003 | 0.81 (0.42, 1.59) | 0.547 |
|  | ≥110 | 2.62 (1.37, 4.98) | 0.003 | 2.05 (1.09, 3.86) | 0.026 | 3.51 (1.42, 8.70) | 0.007 | 1.63 (0.66, 3.98) | 0.287 |
| 45-59 years | <60 | 0.94 (0.79, 1.13) | 0.521 | 0.84 (0.73, 0.96) | 0.008 | 1.53 (1.13, 2.07) | 0.006 | 1.32 (1.14, 1.53) | <0.001 |
|  | 60-69 | 0.88 (0.81, 0.97) | 0.007 | 0.90 (0.84, 0.96) | 0.001 | 1.01 (0.84, 1.20) | 0.954 | 1.07 (0.99, 1.16) | 0.105 |
|  | 70-79 | Ref |  | Ref |  | Ref |  | Ref |  |
|  | 80-89 | 1.05 (0.97, 1.13) | 0.214 | 1.14 (1.09, 1.20) | <0.001 | 1.18 (1.02, 1.37) | 0.027 | 1.04 (0.97, 1.12) | 0.272 |
|  | 90-99 | 1.09 (0.98, 1.22) | 0.102 | 1.28 (1.18, 1.37) | <0.001 | 1.49 (1.24, 1.80) | <0.001 | 1.26 (1.14, 1.39) | <0.001 |
|  | 100-109 | 1.72 (1.39, 2.13) | <0.001 | 1.75 (1.50, 2.04) | <0.001 | 1.96 (1.37, 2.80) | <0.001 | 1.20 (0.93, 1.53) | 0.158 |
|  | ≥110 | 1.96 (1.23, 3.12) | 0.005 | 1.98 (1.42, 2.77) | <0.001 | 2.31 (1.22, 4.35) | 0.010 | 1.59 (1.01, 2.51) | 0.045 |
| 60-74 years | <60 | 1.05 (0.91, 1.21) | 0.498 | 1.04 (0.95, 1.13) | 0.423 | 1.03 (0.76, 1.39) | 0.840 | 1.13 (1.02, 1.24) | 0.020 |
|  | 60-69 | 1.05 (0.97, 1.13) | 0.263 | 1.01 (0.96, 1.06) | 0.656 | 0.92 (0.77, 1.09) | 0.319 | 1.06 (1.00, 1.12) | 0.057 |
|  | 70-79 | Ref |  | Ref |  | Ref |  | Ref |  |
|  | 80-89 | 1.02 (0.95, 1.11) | 0.532 | 1.06 (1.01, 1.11) | 0.018 | 1.04 (0.90, 1.21) | 0.587 | 1.04 (0.98, 1.10) | 0.166 |
|  | 90-99 | 1.20 (1.07, 1.35) | 0.002 | 1.17 (1.09, 1.27) | <0.001 | 1.19 (0.96, 1.47) | 0.114 | 1.12 (1.02, 1.23) | 0.013 |
|  | 100-109 | 1.65 (1.25, 2.17) | <0.001 | 1.61 (1.34, 1.94) | <0.001 | 1.00 (0.63, 1.59) | 0.995 | 1.55 (1.26, 1.92) | <0.001 |
|  | ≥110 | 1.80 (0.99, 3.26) | 0.052 | 2.15 (1.43, 3.25) | <0.001 | 2.01 (0.83, 4.87) | 0.123 | 1.27 (0.72, 2.23) | 0.414 |
| ≥75 years | <60 | 1.08 (0.88, 1.34) | 0.464 | 0.94 (0.81, 1.08) | 0.363 | 0.71 (0.45, 1.12) | 0.142 | 1.09 (0.98, 1.22) | 0.125 |
|  | 60-69 | 0.96 (0.83, 1.11) | 0.578 | 0.94 (0.85, 1.04) | 0.206 | 0.81 (0.60, 1.09) | 0.158 | 1.06 (0.98, 1.15) | 0.120 |
|  | 70-79 | Ref |  | Ref |  | Ref |  | Ref |  |
|  | 80-89 | 0.97 (0.82, 1.16) | 0.756 | 0.98 (0.87, 1.10) | 0.729 | 0.80 (0.58, 1.11) | 0.179 | 0.98 (0.89, 1.08) | 0.736 |
|  | 90-99 | 1.34 (1.02, 1.75) | 0.034 | 1.18 (0.97, 1.44) | 0.101 | 0.61 (0.33, 1.11) | 0.105 | 1.12 (0.95, 1.31) | 0.189 |
|  | 100-109 | 1.72 (0.81, 3.64) | 0.157 | 1.40 (0.75, 2.61) | 0.292 | 2.16 (0.87, 5.37) | 0.099 | 1.74 (1.14, 2.65) | 0.011 |
|  | ≥110 | 1.10 (0.15, 7.87) | 0.922 | 0.56 (0.08, 3.98) | 0.562 | NA |  | 0.94 (0.30, 2.92) | 0.913 |

NA: not available due to small number of events.

The analysis is adjusted for calendar year at assessment, age, sex, diabetes duration, BMI, smoking, HbA1c, LDL cholesterol, HDL cholesterol, log triglycerides, presence of albuminuria, history of CKD and history of CVD.

**Supplementary Table S17:** Cox regression models for the associations of different systolic blood pressure (SBP) cut-offs (referenced to SBP 120 – 129 mmHg) with incident events stratified by age groups, accounting for competing risk of death.

| Age group | SBP categories  (mmHg) | Cardiovascular disease | | Chronic kidney disease | | Kidney failure | |
| --- | --- | --- | --- | --- | --- | --- | --- |
|  |  | sHR (95% CI) | P-value | sHR (95% CI) | P-value | sHR (95% CI) | P-value |
| 18-44 years | <120 | 0.95 (0.81, 1.10) | 0.470 | 0.94 (0.84, 1.06) | 0.310 | 0.93 (0.75, 1.16) | 0.540 |
|  | 120-129 | Ref |  | Ref |  | Ref |  |
|  | 130-139 | 1.04 (0.90, 1.21) | 0.590 | 1.11 (0.99, 1.25) | 0.069 | 1.14 (0.92, 1.43) | 0.240 |
|  | 140-149 | 1.23 (1.05, 1.44) | 0.011 | 1.37 (1.22, 1.55) | <0.001 | 1.60 (1.27, 2.02) | <0.001 |
|  | 150-159 | 1.36 (1.13, 1.64) | 0.001 | 1.52 (1.32, 1.76) | <0.001 | 1.84 (1.42, 2.38) | <0.001 |
|  | 160-169 | 1.96 (1.57, 2.47) | <0.001 | 1.64 (1.35, 1.98) | <0.001 | 1.94 (1.39, 2.71) | <0.001 |
|  | 170-179 | 1.93 (1.29, 2.87) | 0.001 | 2.15 (1.58, 2.92) | <0.001 | 3.30 (2.10, 5.19) | <0.001 |
|  | ≥180 | 2.47 (1.65, 3.71) | <0.001 | 2.94 (2.12, 4.08) | <0.001 | 3.35 (1.97, 5.71) | <0.001 |
| 45-59 years | <120 | 0.98 (0.92, 1.04) | 0.490 | 0.99 (0.95, 1.03) | 0.530 | 0.99 (0.88, 1.10) | 0.780 |
|  | 120-129 | Ref |  | Ref |  | Ref |  |
|  | 130-139 | 1.08 (1.01, 1.14) | 0.016 | 1.06 (1.02, 1.10) | 0.005 | 1.01 (0.91, 1.12) | 0.880 |
|  | 140-149 | 1.17 (1.10, 1.24) | <0.001 | 1.17 (1.12, 1.22) | <0.001 | 1.33 (1.20, 1.47) | <0.001 |
|  | 150-159 | 1.25 (1.16, 1.34) | <0.001 | 1.34 (1.28, 1.40) | <0.001 | 1.56 (1.40, 1.74) | <0.001 |
|  | 160-169 | 1.33 (1.21, 1.46) | <0.001 | 1.44 (1.36, 1.53) | <0.001 | 1.70 (1.49, 1.95) | <0.001 |
|  | 170-179 | 1.60 (1.41, 1.82) | <0.001 | 1.62 (1.48, 1.78) | <0.001 | 2.27 (1.91, 2.68) | <0.001 |
|  | ≥180 | 1.85 (1.62, 2.12) | <0.001 | 1.99 (1.80, 2.20) | <0.001 | 2.45 (2.07, 2.92) | <0.001 |
| 60-74 years | <120 | 0.99 (0.93, 1.04) | 0.610 | 1.03 (1.00, 1.06) | 0.070 | 1.03 (0.94, 1.13) | 0.490 |
|  | 120-129 | Ref |  | Ref |  | Ref |  |
|  | 130-139 | 0.99 (0.94, 1.04) | 0.610 | 1.05 (1.02, 1.08) | <0.001 | 1.09 (1.00, 1.18) | 0.040 |
|  | 140-149 | 1.05 (1.00, 1.11) | 0.043 | 1.10 (1.06, 1.13) | <0.001 | 1.27 (1.18, 1.38) | <0.001 |
|  | 150-159 | 1.13 (1.07, 1.19) | <0.001 | 1.16 (1.13, 1.20) | <0.001 | 1.42 (1.31, 1.54) | <0.001 |
|  | 160-169 | 1.19 (1.11, 1.27) | <0.001 | 1.21 (1.16, 1.26) | <0.001 | 1.53 (1.39, 1.68) | <0.001 |
|  | 170-179 | 1.25 (1.15, 1.37) | <0.001 | 1.29 (1.22, 1.37) | <0.001 | 1.72 (1.54, 1.94) | <0.001 |
|  | ≥180 | 1.42 (1.29, 1.55) | <0.001 | 1.45 (1.36, 1.55) | <0.001 | 2.08 (1.85, 2.33) | <0.001 |
| ≥75 years | <120 | 1.12 (1.02, 1.23) | 0.013 | 1.03 (0.97, 1.09) | 0.330 | 1.13 (0.98, 1.30) | 0.090 |
|  | 120-129 | Ref |  | Ref |  | Ref |  |
|  | 130-139 | 1.09 (1.01, 1.18) | 0.036 | 1.02 (0.98, 1.08) | 0.330 | 1.32 (1.18, 1.49) | <0.001 |
|  | 140-149 | 1.09 (1.01, 1.18) | 0.025 | 1.08 (1.03, 1.13) | 0.003 | 1.40 (1.24, 1.57) | <0.001 |
|  | 150-159 | 1.10 (1.01, 1.19) | 0.026 | 1.10 (1.05, 1.17) | <0.001 | 1.54 (1.37, 1.73) | <0.001 |
|  | 160-169 | 1.26 (1.14, 1.39) | <0.001 | 1.19 (1.12, 1.28) | <0.001 | 1.46 (1.28, 1.67) | <0.001 |
|  | 170-179 | 1.31 (1.17, 1.48) | <0.001 | 1.28 (1.17, 1.40) | <0.001 | 1.66 (1.42, 1.95) | <0.001 |
|  | ≥180 | 1.31 (1.16, 1.49) | <0.001 | 1.27 (1.15, 1.40) | <0.001 | 1.83 (1.57, 2.14) | <0.001 |

sHR: subdistribution hazard ratio.

The analysis is adjusted for calendar year at assessment, age, sex, diabetes duration, BMI, smoking, HbA1c, LDL cholesterol, HDL cholesterol, log triglycerides, presence of albuminuria, history of CKD, history of CVD and use of blood pressure lowering medications.

**Supplementary Table S18:** Cox regression models for the associations of different diastolic blood pressure (DBP) cut-offs (referenced to DBP 70 – 79 mmHg) with incident events stratified by age groups, accounting for competing risk of death.

| Age group | DBP categories  (mmHg) | Cardiovascular disease | | Chronic kidney disease | | Kidney failure | |
| --- | --- | --- | --- | --- | --- | --- | --- |
|  |  | sHR (95% CI) | P-value | sHR (95% CI) | P-value | sHR (95% CI) | P-value |
| 18-44 years | <60 | 0.94 (0.64, 1.38) | 0.750 | 0.55 (0.39, 0.78) | <0.001 | 0.49 (0.27, 0.91) | 0.024 |
|  | 60-69 | 0.91 (0.77, 1.09) | 0.310 | 0.87 (0.77, 1.00) | 0.045 | 0.82 (0.65, 1.04) | 0.100 |
|  | 70-79 | Ref |  | Ref |  | Ref |  |
|  | 80-89 | 1.13 (1.00, 1.28) | 0.046 | 1.15 (1.05, 1.26) | 0.004 | 1.14 (0.96, 1.36) | 0.140 |
|  | 90-99 | 1.23 (1.06, 1.43) | 0.005 | 1.30 (1.16, 1.46) | <0.001 | 1.44 (1.18, 1.76) | <0.001 |
|  | 100-109 | 1.54 (1.19, 1.98) | <0.001 | 1.74 (1.45, 2.08) | <0.001 | 1.46 (1.05, 2.03) | 0.026 |
|  | ≥110 | 2.34 (1.61, 3.39) | <0.001 | 1.75 (1.23, 2.49) | 0.002 | 2.07 (1.18, 3.66) | 0.012 |
| 45-59 years | <60 | 1.05 (0.92, 1.19) | 0.490 | 0.99 (0.91, 1.08) | 0.900 | 1.07 (0.88, 1.30) | 0.510 |
|  | 60-69 | 0.97 (0.91, 1.03) | 0.360 | 0.97 (0.93, 1.01) | 0.180 | 0.96 (0.87, 1.06) | 0.470 |
|  | 70-79 | Ref |  | Ref |  | Ref |  |
|  | 80-89 | 1.00 (0.96, 1.05) | 0.830 | 1.07 (1.04, 1.11) | <0.001 | 1.03 (0.95, 1.11) | 0.500 |
|  | 90-99 | 1.08 (1.02, 1.15) | 0.009 | 1.20 (1.15, 1.25) | <0.001 | 1.20 (1.09, 1.31) | <0.001 |
|  | 100-109 | 1.38 (1.23, 1.55) | <0.001 | 1.41 (1.31, 1.53) | <0.001 | 1.23 (1.03, 1.47) | 0.023 |
|  | ≥110 | 1.93 (1.54, 2.42) | <0.001 | 1.65 (1.37, 1.98) | <0.001 | 1.63 (1.21, 2.20) | 0.001 |
| 60-74 years | <60 | 1.09 (1.01, 1.17) | 0.020 | 1.11 (1.06, 1.16) | <0.001 | 1.05 (0.95, 1.16) | 0.320 |
|  | 60-69 | 1.01 (0.97, 1.05) | 0.600 | 1.02 (1.00, 1.05) | 0.110 | 1.08 (1.02, 1.15) | 0.008 |
|  | 70-79 | Ref |  | Ref |  | Ref |  |
|  | 80-89 | 1.01 (0.97, 1.05) | 0.570 | 1.02 (0.99, 1.04) | 0.150 | 1.03 (0.98, 1.09) | 0.270 |
|  | 90-99 | 1.13 (1.07, 1.20) | <0.001 | 1.11 (1.07, 1.15) | <0.001 | 1.08 (0.99, 1.17) | 0.072 |
|  | 100-109 | 1.29 (1.13, 1.48) | <0.001 | 1.29 (1.18, 1.40) | <0.001 | 1.29 (1.07, 1.55) | 0.006 |
|  | ≥110 | 1.75 (1.32, 2.31) | <0.001 | 1.84 (1.53, 2.20) | <0.001 | 1.38 (0.98, 1.94) | 0.063 |
| ≥75 years | <60 | 1.08 (1.00, 1.17) | 0.042 | 1.07 (1.02, 1.13) | 0.007 | 1.15 (1.04, 1.26) | 0.006 |
|  | 60-69 | 0.98 (0.92, 1.03) | 0.390 | 1.04 (1.00, 1.07) | 0.059 | 1.05 (0.98, 1.14) | 0.180 |
|  | 70-79 | Ref |  | Ref |  | Ref |  |
|  | 80-89 | 1.04 (0.98, 1.11) | 0.210 | 1.02 (0.98, 1.07) | 0.350 | 0.98 (0.89, 1.08) | 0.680 |
|  | 90-99 | 1.16 (1.04, 1.29) | 0.008 | 1.14 (1.06, 1.24) | <0.001 | 0.87 (0.74, 1.02) | 0.092 |
|  | 100-109 | 1.35 (1.04, 1.74) | 0.022 | 1.28 (1.02, 1.59) | 0.030 | 1.22 (0.88, 1.70) | 0.240 |
|  | ≥110 | 1.54 (0.88, 2.69) | 0.130 | 0.88 (0.50, 1.55) | 0.670 | 0.89 (0.38, 2.11) | 0.800 |

sHR: subdistribution hazard ratio

The analysis is adjusted for calendar year at assessment, age, sex, diabetes duration, BMI, smoking, HbA1c, LDL cholesterol, HDL cholesterol, log triglycerides, presence of albuminuria, history of CKD, history of CVD and use of blood pressure lowering medications.

**Supplementary Table S19**: Cox regression models for the associations of different systolic blood pressure (SBP) cut-offs (referenced to SBP 120 – 129 mmHg) with incident events, stratified by age groups. This analysis excluded individuals who had history of cardiovascular disease or chronic kidney disease, and those who developed cardiovascular disease, chronic kidney disease or all-cause death within 1 year of study entry (n = 303,949).

| Age group | SBP (mmHg) | Cardiovascular disease | | Chronic kidney disease | | Kidney failure | | All-cause death | |
| --- | --- | --- | --- | --- | --- | --- | --- | --- | --- |
|  |  | HR (95% CI) | P-value | HR (95% CI) | P-value | HR (95% CI) | P-value | HR (95% CI) | P-value |
| 18-44 years | <120 | 0.99 (0.84, 1.16) | 0.921 | 0.95 (0.84, 1.08) | 0.419 | 1.01 (0.78, 1.30) | 0.951 | 1.11 (0.93, 1.33) | 0.245 |
|  | 120-129 | Ref |  | Ref |  | Ref |  | Ref |  |
|  | 130-139 | 1.11 (0.95, 1.30) | 0.202 | 1.12 (0.99, 1.26) | 0.081 | 1.05 (0.80, 1.37) | 0.742 | 0.99 (0.82, 1.20) | 0.912 |
|  | 140-149 | 1.30 (1.10, 1.54) | 0.002 | 1.35 (1.19, 1.54) | <0.001 | 1.53 (1.16, 2.01) | 0.002 | 1.21 (0.99, 1.49) | 0.066 |
|  | 150-159 | 1.31 (1.07, 1.62) | 0.010 | 1.47 (1.26, 1.71) | <0.001 | 1.53 (1.09, 2.15) | 0.014 | 1.35 (1.05, 1.73) | 0.019 |
|  | 160-169 | 1.95 (1.51, 2.52) | <0.001 | 1.56 (1.27, 1.93) | <0.001 | 1.94 (1.26, 2.98) | 0.002 | 1.42 (1.01, 2.01) | 0.045 |
|  | 170-179 | 1.88 (1.22, 2.88) | 0.004 | 2.27 (1.66, 3.11) | <0.001 | 2.67 (1.43, 5.00) | 0.002 | 1.60 (0.91, 2.82) | 0.100 |
|  | ≥180 | 2.22 (1.37, 3.58) | 0.001 | 2.74 (1.94, 3.88) | <0.001 | 3.64 (1.89, 7.01) | <0.001 | 2.66 (1.54, 4.58) | <0.001 |
| 45-59 years | <120 | 0.96 (0.90, 1.03) | 0.249 | 0.96 (0.92, 1.01) | 0.101 | 0.98 (0.84, 1.15) | 0.838 | 1.07 (1.00, 1.14) | 0.064 |
|  | 120-129 | Ref |  | Ref |  | Ref |  | Ref |  |
|  | 130-139 | 1.04 (0.98, 1.11) | 0.186 | 1.04 (1.00, 1.09) | 0.074 | 0.95 (0.82, 1.10) | 0.469 | 0.95 (0.89, 1.02) | 0.142 |
|  | 140-149 | 1.13 (1.06, 1.21) | <0.001 | 1.18 (1.13, 1.23) | <0.001 | 1.18 (1.01, 1.36) | 0.034 | 1.09 (1.02, 1.17) | 0.016 |
|  | 150-159 | 1.22 (1.12, 1.31) | <0.001 | 1.30 (1.23, 1.36) | <0.001 | 1.33 (1.13, 1.56) | <0.001 | 1.15 (1.05, 1.25) | 0.001 |
|  | 160-169 | 1.31 (1.18, 1.45) | <0.001 | 1.39 (1.30, 1.48) | <0.001 | 1.88 (1.55, 2.28) | <0.001 | 1.30 (1.16, 1.45) | <0.001 |
|  | 170-179 | 1.42 (1.21, 1.65) | <0.001 | 1.53 (1.39, 1.69) | <0.001 | 1.96 (1.51, 2.54) | <0.001 | 1.47 (1.26, 1.72) | <0.001 |
|  | ≥180 | 1.64 (1.38, 1.95) | <0.001 | 2.04 (1.84, 2.27) | <0.001 | 2.67 (2.05, 3.48) | <0.001 | 1.91 (1.62, 2.26) | <0.001 |
| 60-74 years | <120 | 1.00 (0.93, 1.07) | 0.954 | 1.03 (0.99, 1.07) | 0.130 | 0.96 (0.78, 1.17) | 0.674 | 1.05 (0.99, 1.11) | 0.094 |
|  | 120-129 | Ref |  | Ref |  | Ref |  | Ref |  |
|  | 130-139 | 0.99 (0.94, 1.06) | 0.845 | 1.05 (1.02, 1.09) | 0.003 | 1.14 (0.97, 1.35) | 0.120 | 0.96 (0.91, 1.01) | 0.115 |
|  | 140-149 | 1.03 (0.97, 1.09) | 0.407 | 1.09 (1.05, 1.13) | <0.001 | 1.22 (1.03, 1.44) | 0.023 | 1.03 (0.98, 1.08) | 0.252 |
|  | 150-159 | 1.12 (1.05, 1.19) | 0.001 | 1.15 (1.11, 1.20) | <0.001 | 1.28 (1.08, 1.53) | 0.005 | 1.04 (0.98, 1.10) | 0.205 |
|  | 160-169 | 1.18 (1.09, 1.28) | <0.001 | 1.21 (1.16, 1.27) | <0.001 | 1.59 (1.31, 1.94) | <0.001 | 1.16 (1.09, 1.25) | <0.001 |
|  | 170-179 | 1.20 (1.06, 1.35) | 0.003 | 1.34 (1.25, 1.43) | <0.001 | 1.84 (1.43, 2.37) | <0.001 | 1.32 (1.20, 1.45) | <0.001 |
|  | ≥180 | 1.54 (1.36, 1.74) | <0.001 | 1.43 (1.32, 1.54) | <0.001 | 2.71 (2.14, 3.45) | <0.001 | 1.39 (1.26, 1.55) | <0.001 |
| ≥75 years | <120 | 1.16 (1.01, 1.33) | 0.033 | 1.01 (0.94, 1.09) | 0.730 | 0.93 (0.54, 1.60) | 0.782 | 1.04 (0.96, 1.13) | 0.372 |
|  | 120-129 | Ref |  | Ref |  | Ref |  | Ref |  |
|  | 130-139 | 1.02 (0.91, 1.15) | 0.683 | 1.02 (0.96, 1.09) | 0.478 | 1.20 (0.79, 1.82) | 0.402 | 0.94 (0.87, 1.00) | 0.068 |
|  | 140-149 | 1.02 (0.91, 1.15) | 0.746 | 1.05 (0.98, 1.12) | 0.148 | 1.47 (0.99, 2.20) | 0.059 | 0.91 (0.85, 0.98) | 0.014 |
|  | 150-159 | 1.04 (0.92, 1.18) | 0.539 | 1.07 (1.00, 1.15) | 0.049 | 1.51 (0.99, 2.29) | 0.054 | 1.01 (0.93, 1.09) | 0.862 |
|  | 160-169 | 1.19 (1.03, 1.38) | 0.022 | 1.14 (1.05, 1.24) | 0.003 | 1.46 (0.89, 2.38) | 0.132 | 0.99 (0.90, 1.08) | 0.752 |
|  | 170-179 | 1.42 (1.18, 1.71) | <0.001 | 1.29 (1.15, 1.44) | <0.001 | 1.49 (0.82, 2.71) | 0.191 | 1.13 (1.00, 1.27) | 0.044 |
|  | ≥180 | 1.48 (1.21, 1.80) | <0.001 | 1.25 (1.10, 1.41) | <0.001 | 2.49 (1.46, 4.26) | <0.001 | 1.16 (1.02, 1.32) | 0.024 |

The analysis is adjusted for calendar year at assessment, age, sex, diabetes duration, BMI, smoking, HbA1c, LDL cholesterol, HDL cholesterol, log triglycerides, presence of albuminuria and use of blood pressure lowering medications.

**Supplementary Table S20**: Cox regression models for the associations of different diastolic blood pressure (DBP) cut-offs (referenced to DBP 70 – 79 mmHg) with incident events, stratified by age groups. This analysis excluded individuals who had history of cardiovascular disease or chronic kidney disease, and those who developed cardiovascular disease, chronic kidney disease or all-cause death within 1 year of study entry (n = 303,949).

| Age group | DBP (mmHg) | Cardiovascular disease | | Chronic Kidney Disease | | Kidney failure | | All-cause death | |
| --- | --- | --- | --- | --- | --- | --- | --- | --- | --- |
|  |  | HR (95% CI) | P-value | HR (95% CI) | P-value | HR (95% CI) | P-value | HR (95% CI) | P-value |
| 18-44 years | <60 | 1.03 (0.70, 1.52) | 0.880 | 0.52 (0.35, 0.76) | <0.001 | 0.43 (0.19, 0.97) | 0.043 | 1.18 (0.82, 1.70) | 0.380 |
|  | 60-69 | 0.91 (0.75, 1.10) | 0.314 | 0.84 (0.73, 0.97) | 0.018 | 0.64 (0.47, 0.87) | 0.005 | 0.97 (0.80, 1.19) | 0.785 |
|  | 70-79 | Ref |  | Ref |  | Ref |  | Ref |  |
|  | 80-89 | 1.14 (1.00, 1.30) | 0.043 | 1.15 (1.04, 1.27) | 0.007 | 1.07 (0.87, 1.32) | 0.508 | 0.98 (0.84, 1.14) | 0.749 |
|  | 90-99 | 1.20 (1.02, 1.42) | 0.024 | 1.27 (1.12, 1.44) | <0.001 | 1.19 (0.91, 1.54) | 0.203 | 1.10 (0.90, 1.34) | 0.343 |
|  | 100-109 | 1.47 (1.12, 1.94) | 0.006 | 1.74 (1.42, 2.12) | <0.001 | 1.34 (0.84, 2.13) | 0.216 | 1.21 (0.84, 1.74) | 0.300 |
|  | ≥110 | 2.56 (1.73, 3.79) | <0.001 | 1.68 (1.17, 2.42) | 0.005 | 2.78 (1.54, 5.04) | <0.001 | 2.06 (1.22, 3.48) | 0.007 |
| 45-59 years | <60 | 1.05 (0.91, 1.21) | 0.503 | 0.99 (0.90, 1.09) | 0.903 | 1.17 (0.86, 1.58) | 0.318 | 1.31 (1.15, 1.49) | <0.001 |
|  | 60-69 | 0.97 (0.90, 1.04) | 0.336 | 0.94 (0.90, 0.99) | 0.014 | 0.88 (0.76, 1.03) | 0.120 | 1.07 (1.00, 1.15) | 0.050 |
|  | 70-79 | Ref |  | Ref |  | Ref |  | Ref |  |
|  | 80-89 | 1.01 (0.96, 1.06) | 0.803 | 1.06 (1.02, 1.09) | 0.001 | 1.00 (0.90, 1.12) | 0.998 | 0.96 (0.91, 1.01) | 0.154 |
|  | 90-99 | 1.06 (0.99, 1.13) | 0.085 | 1.17 (1.12, 1.23) | <0.001 | 1.10 (0.96, 1.27) | 0.158 | 1.10 (1.02, 1.18) | 0.009 |
|  | 100-109 | 1.38 (1.21, 1.58) | <0.001 | 1.40 (1.28, 1.53) | <0.001 | 1.29 (0.99, 1.67) | 0.061 | 1.22 (1.05, 1.41) | 0.010 |
|  | ≥110 | 1.67 (1.25, 2.23) | <0.001 | 1.61 (1.32, 1.96) | <0.001 | 1.31 (0.76, 2.27) | 0.336 | 1.72 (1.27, 2.33) | <0.001 |
| 60-74 years | <60 | 1.08 (0.98, 1.18) | 0.111 | 1.10 (1.05, 1.16) | <0.001 | 0.89 (0.69, 1.15) | 0.380 | 1.18 (1.09, 1.26) | <0.001 |
|  | 60-69 | 1.01 (0.96, 1.07) | 0.599 | 1.02 (0.99, 1.05) | 0.197 | 0.98 (0.86, 1.12) | 0.777 | 1.10 (1.05, 1.14) | <0.001 |
|  | 70-79 | Ref |  | Ref |  | Ref |  | Ref |  |
|  | 80-89 | 1.00 (0.95, 1.05) | 0.938 | 0.99 (0.97, 1.02) | 0.633 | 0.97 (0.86, 1.09) | 0.567 | 1.01 (0.97, 1.05) | 0.621 |
|  | 90-99 | 1.13 (1.05, 1.21) | <0.001 | 1.09 (1.05, 1.14) | <0.001 | 1.09 (0.92, 1.30) | 0.295 | 1.10 (1.03, 1.17) | 0.002 |
|  | 100-109 | 1.28 (1.08, 1.52) | 0.004 | 1.22 (1.10, 1.35) | <0.001 | 1.55 (1.09, 2.21) | 0.014 | 1.39 (1.21, 1.60) | <0.001 |
|  | ≥110 | 2.11 (1.49, 2.99) | <0.001 | 2.10 (1.68, 2.62) | <0.001 | 1.28 (0.48, 3.43) | 0.621 | 2.17 (1.60, 2.96) | <0.001 |
| ≥75 years | <60 | 1.17 (1.04, 1.32) | 0.009 | 1.09 (1.02, 1.16) | 0.015 | 0.98 (0.65, 1.49) | 0.933 | 1.11 (1.03, 1.19) | 0.007 |
|  | 60-69 | 0.98 (0.90, 1.07) | 0.695 | 1.06 (1.01, 1.11) | 0.014 | 1.05 (0.80, 1.38) | 0.713 | 1.06 (1.00, 1.11) | 0.038 |
|  | 70-79 | Ref |  | Ref |  | Ref |  | Ref |  |
|  | 80-89 | 1.01 (0.91, 1.12) | 0.832 | 1.01 (0.96, 1.07) | 0.705 | 1.19 (0.88, 1.60) | 0.267 | 1.08 (1.01, 1.15) | 0.015 |
|  | 90-99 | 1.24 (1.05, 1.46) | 0.011 | 1.12 (1.01, 1.24) | 0.027 | 1.02 (0.59, 1.75) | 0.942 | 1.04 (0.93, 1.16) | 0.513 |
|  | 100-109 | 1.65 (1.12, 2.43) | 0.012 | 1.50 (1.16, 1.95) | 0.002 | 1.99 (0.73, 5.41) | 0.178 | 1.45 (1.11, 1.91) | 0.008 |
|  | ≥110 | 1.92 (0.72, 5.13) | 0.193 | 0.71 (0.32, 1.58) | 0.396 | NA |  | 1.15 (0.52, 2.56) | 0.733 |

NA: not available due to small number of events

The analysis is adjusted for calendar year at assessment, age, sex, diabetes duration, BMI, smoking, HbA1c, LDL cholesterol, HDL cholesterol, log triglycerides, presence of albuminuria and use of blood pressure lowering medications.

**Supplementary Table S21**: Cox regression models for the associations of different systolic blood pressure (SBP) cut-offs (referenced to SBP 120 – 129 mmHg) with incident events, stratified by age groups, using multiple imputation approach (n = 573,780).

| Age group | SBP (mmHg) | Cardiovascular disease | | Chronic kidney disease | | Kidney failure | | All-cause death | |
| --- | --- | --- | --- | --- | --- | --- | --- | --- | --- |
|  |  | HR (95% CI) | P-value | HR (95% CI) | P-value | HR (95% CI) | P-value | HR (95% CI) | P-value |
| 18-44 years | <120 | 0.97 (0.84, 1.13) | 0.715 | 0.97 (0.86, 1.08) | 0.542 | 0.93 (0.76, 1.13) | 0.458 | 1.09 (0.94, 1.27) | 0.248 |
|  | 120-129 | Ref |  | Ref |  | Ref |  | Ref |  |
|  | 130-139 | 1.04 (0.90, 1.21) | 0.560 | 1.11 (1.00, 1.24) | 0.060 | 1.08 (0.88, 1.33) | 0.472 | 1.03 (0.88, 1.21) | 0.732 |
|  | 140-149 | 1.21 (1.04, 1.41) | 0.015 | 1.39 (1.23, 1.56) | <0.001 | 1.57 (1.27, 1.92) | <0.001 | 1.22 (1.03, 1.44) | 0.022 |
|  | 150-159 | 1.38 (1.15, 1.66) | <0.001 | 1.54 (1.34, 1.76) | <0.001 | 1.77 (1.41, 2.24) | <0.001 | 1.40 (1.15, 1.71) | <0.001 |
|  | 160-169 | 1.98 (1.59, 2.48) | <0.001 | 1.64 (1.36, 1.98) | <0.001 | 1.79 (1.34, 2.40) | <0.001 | 1.60 (1.25, 2.06) | <0.001 |
|  | 170-179 | 1.84 (1.25, 2.69) | 0.002 | 2.09 (1.59, 2.73) | <0.001 | 3.17 (2.11, 4.76) | <0.001 | 1.83 (1.22, 2.74) | 0.004 |
|  | ≥180 | 2.49 (1.71, 3.64) | <0.001 | 2.88 (2.18, 3.80) | <0.001 | 3.35 (2.23, 5.04) | <0.001 | 2.70 (1.90, 3.85) | <0.001 |
| 45-59 years | <120 | 0.98 (0.93, 1.04) | 0.593 | 1.00 (0.96, 1.04) | 0.906 | 1.02 (0.92, 1.12) | 0.773 | 1.13 (1.07, 1.19) | <0.001 |
|  | 120-129 | Ref |  | Ref |  | Ref |  | Ref |  |
|  | 130-139 | 1.06 (1.01, 1.13) | 0.033 | 1.06 (1.02, 1.10) | 0.003 | 1.03 (0.93, 1.13) | 0.556 | 0.99 (0.94, 1.05) | 0.772 |
|  | 140-149 | 1.16 (1.09, 1.23) | <0.001 | 1.18 (1.13, 1.22) | <0.001 | 1.33 (1.21, 1.47) | <0.001 | 1.12 (1.06, 1.18) | <0.001 |
|  | 150-159 | 1.25 (1.16, 1.34) | <0.001 | 1.34 (1.29, 1.40) | <0.001 | 1.58 (1.43, 1.75) | <0.001 | 1.21 (1.14, 1.29) | <0.001 |
|  | 160-169 | 1.33 (1.22, 1.46) | <0.001 | 1.46 (1.37, 1.54) | <0.001 | 1.71 (1.52, 1.92) | <0.001 | 1.41 (1.30, 1.52) | <0.001 |
|  | 170-179 | 1.64 (1.46, 1.86) | <0.001 | 1.67 (1.54, 1.81) | <0.001 | 2.35 (2.04, 2.71) | <0.001 | 1.60 (1.44, 1.77) | <0.001 |
|  | ≥180 | 1.97 (1.73, 2.24) | <0.001 | 2.10 (1.93, 2.30) | <0.001 | 2.70 (2.34, 3.10) | <0.001 | 2.05 (1.85, 2.27) | <0.001 |
| 60-74 years | <120 | 1.00 (0.95, 1.06) | 0.871 | 1.04 (1.01, 1.07) | 0.014 | 1.04 (0.95, 1.14) | 0.372 | 1.07 (1.04, 1.11) | <0.001 |
|  | 120-129 | Ref |  | Ref |  | Ref |  | Ref |  |
|  | 130-139 | 0.99 (0.94, 1.04) | 0.641 | 1.05 (1.02, 1.08) | 0.002 | 1.07 (0.99, 1.15) | 0.111 | 0.98 (0.95, 1.01) | 0.177 |
|  | 140-149 | 1.06 (1.01, 1.11) | 0.025 | 1.10 (1.07, 1.13) | <0.001 | 1.26 (1.17, 1.36) | <0.001 | 1.04 (1.01, 1.07) | 0.023 |
|  | 150-159 | 1.14 (1.08, 1.20) | <0.001 | 1.17 (1.13, 1.21) | <0.001 | 1.43 (1.33, 1.55) | <0.001 | 1.07 (1.04, 1.11) | <0.001 |
|  | 160-169 | 1.21 (1.13, 1.29) | <0.001 | 1.21 (1.17, 1.26) | <0.001 | 1.57 (1.44, 1.71) | <0.001 | 1.16 (1.12, 1.21) | <0.001 |
|  | 170-179 | 1.32 (1.21, 1.44) | <0.001 | 1.34 (1.27, 1.41) | <0.001 | 1.88 (1.69, 2.09) | <0.001 | 1.36 (1.29, 1.43) | <0.001 |
|  | ≥180 | 1.52 (1.39, 1.66) | <0.001 | 1.46 (1.38, 1.55) | <0.001 | 2.19 (1.98, 2.42) | <0.001 | 1.45 (1.37, 1.53) | <0.001 |
| ≥75 years | <120 | 1.12 (1.03, 1.22) | 0.008 | 1.05 (0.99, 1.11) | 0.117 | 1.17 (1.02, 1.33) | 0.021 | 1.09 (1.05, 1.14) | <0.001 |
|  | 120-129 | Ref |  | Ref |  | Ref |  | Ref |  |
|  | 130-139 | 1.04 (0.97, 1.12) | 0.263 | 1.02 (0.97, 1.07) | 0.355 | 1.30 (1.17, 1.46) | <0.001 | 0.95 (0.92, 0.98) | 0.005 |
|  | 140-149 | 1.06 (0.99, 1.14) | 0.117 | 1.06 (1.01, 1.11) | 0.020 | 1.36 (1.22, 1.52) | <0.001 | 0.96 (0.93, 0.99) | 0.025 |
|  | 150-159 | 1.08 (1.00, 1.17) | 0.063 | 1.10 (1.05, 1.16) | <0.001 | 1.50 (1.34, 1.68) | <0.001 | 0.99 (0.96, 1.03) | 0.763 |
|  | 160-169 | 1.24 (1.13, 1.35) | <0.001 | 1.18 (1.11, 1.26) | <0.001 | 1.54 (1.36, 1.75) | <0.001 | 1.05 (1.00, 1.10) | 0.043 |
|  | 170-179 | 1.27 (1.14, 1.43) | <0.001 | 1.25 (1.15, 1.36) | <0.001 | 1.63 (1.40, 1.89) | <0.001 | 1.06 (1.00, 1.12) | 0.064 |
|  | ≥180 | 1.40 (1.24, 1.57) | <0.001 | 1.31 (1.20, 1.44) | <0.001 | 1.98 (1.71, 2.29) | <0.001 | 1.19 (1.12, 1.26) | <0.001 |

The analysis is adjusted for calendar year at assessment, age, sex, diabetes duration, BMI, smoking, HbA1c, LDL cholesterol, HDL cholesterol, log triglycerides, presence of albuminuria, history of CKD, history of CVD and use of blood pressure lowering medications.

Multiple imputation was performed for covariates with missing data, including diabetes duration, BMI, HbA1c, LDL cholesterol, HDL cholesterol, and log-transformed triglycerides. Specifically, 25 imputed datasets were created, corresponding to the highest proportion of incomplete cases in the dataset, and the results were pooled using Rubin’s rules.

**Supplementary Table S22**: Cox regression models for the associations of different diastolic blood pressure (DBP) cut-offs (referenced to DBP 70 – 79 mmHg) with incident events, stratified by age groups, using multiple imputation approach (n = 573,718).

| Age group | DBP (mmHg) | Cardiovascular disease | | Chronic Kidney Disease | | Kidney failure | | All-cause death | |
| --- | --- | --- | --- | --- | --- | --- | --- | --- | --- |
|  |  | HR (95% CI) | P-value | HR (95% CI) | P-value | HR (95% CI) | P-value | HR (95% CI) | P-value |
| 18-44 years | <60 | 0.99 (0.69, 1.42) | 0.962 | 0.59 (0.42, 0.82) | 0.001 | 0.53 (0.30, 0.95) | 0.034 | 1.29 (0.95, 1.74) | 0.103 |
|  | 60-69 | 0.93 (0.78, 1.10) | 0.406 | 0.89 (0.78, 1.01) | 0.082 | 0.84 (0.67, 1.06) | 0.143 | 0.99 (0.84, 1.17) | 0.934 |
|  | 70-79 | Ref |  | Ref |  | Ref |  | Ref |  |
|  | 80-89 | 1.13 (1.00, 1.27) | 0.044 | 1.15 (1.05, 1.26) | 0.002 | 1.16 (0.99, 1.36) | 0.067 | 1.07 (0.94, 1.21) | 0.318 |
|  | 90-99 | 1.23 (1.07, 1.42) | 0.005 | 1.31 (1.17, 1.46) | <0.001 | 1.44 (1.20, 1.73) | <0.001 | 1.24 (1.06, 1.44) | 0.006 |
|  | 100-109 | 1.59 (1.25, 2.02) | <0.001 | 1.79 (1.51, 2.13) | <0.001 | 1.49 (1.09, 2.04) | 0.011 | 1.23 (0.93, 1.64) | 0.150 |
|  | ≥110 | 2.32 (1.64, 3.29) | <0.001 | 1.77 (1.32, 2.38) | <0.001 | 2.01 (1.32, 3.06) | 0.001 | 1.86 (1.26, 2.76) | 0.002 |
| 45-59 years | <60 | 1.07 (0.95, 1.22) | 0.260 | 1.01 (0.93, 1.10) | 0.791 | 1.14 (0.95, 1.36) | 0.155 | 1.39 (1.27, 1.53) | <0.001 |
|  | 60-69 | 0.97 (0.92, 1.03) | 0.374 | 0.99 (0.95, 1.02) | 0.456 | 0.99 (0.91, 1.09) | 0.883 | 1.10 (1.04, 1.16) | <0.001 |
|  | 70-79 | Ref |  | Ref |  | Ref |  | Ref |  |
|  | 80-89 | 1.00 (0.96, 1.05) | 0.843 | 1.07 (1.04, 1.10) | <0.001 | 1.02 (0.95, 1.09) | 0.636 | 0.96 (0.92, 1.00) | 0.061 |
|  | 90-99 | 1.09 (1.03, 1.16) | 0.003 | 1.21 (1.17, 1.26) | <0.001 | 1.20 (1.11, 1.31) | <0.001 | 1.11 (1.05, 1.17) | <0.001 |
|  | 100-109 | 1.39 (1.25, 1.55) | <0.001 | 1.44 (1.34, 1.55) | <0.001 | 1.21 (1.05, 1.40) | 0.008 | 1.19 (1.07, 1.32) | <0.001 |
|  | ≥110 | 2.04 (1.64, 2.53) | <0.001 | 1.70 (1.45, 1.98) | <0.001 | 1.91 (1.48, 2.47) | <0.001 | 1.83 (1.52, 2.20) | <0.001 |
| 60-74 years | <60 | 1.14 (1.07, 1.22) | <0.001 | 1.14 (1.09, 1.19) | <0.001 | 1.13 (1.03, 1.23) | 0.011 | 1.26 (1.21, 1.32) | <0.001 |
|  | 60-69 | 1.03 (0.99, 1.07) | 0.126 | 1.03 (1.01, 1.06) | 0.007 | 1.10 (1.04, 1.17) | <0.001 | 1.10 (1.07, 1.12) | <0.001 |
|  | 70-79 | Ref |  | Ref |  | Ref |  | Ref |  |
|  | 80-89 | 1.02 (0.98, 1.06) | 0.271 | 1.02 (1.00, 1.05) | 0.038 | 1.05 (0.99, 1.10) | 0.098 | 1.02 (1.00, 1.05) | 0.073 |
|  | 90-99 | 1.15 (1.08, 1.21) | <0.001 | 1.12 (1.08, 1.16) | <0.001 | 1.10 (1.02, 1.19) | 0.015 | 1.10 (1.06, 1.15) | <0.001 |
|  | 100-109 | 1.35 (1.19, 1.53) | <0.001 | 1.32 (1.23, 1.43) | <0.001 | 1.33 (1.13, 1.56) | <0.001 | 1.32 (1.21, 1.44) | <0.001 |
|  | ≥110 | 1.91 (1.47, 2.48) | <0.001 | 2.03 (1.71, 2.40) | <0.001 | 1.62 (1.17, 2.24) | 0.004 | 1.72 (1.44, 2.06) | <0.001 |
| ≥75 years | <60 | 1.14 (1.06, 1.22) | <0.001 | 1.10 (1.04, 1.15) | <0.001 | 1.25 (1.14, 1.37) | <0.001 | 1.18 (1.14, 1.22) | <0.001 |
|  | 60-69 | 1.00 (0.94, 1.05) | 0.884 | 1.04 (1.01, 1.08) | 0.019 | 1.06 (0.99, 1.14) | 0.099 | 1.05 (1.02, 1.08) | <0.001 |
|  | 70-79 | Ref |  | Ref |  | Ref |  | Ref |  |
|  | 80-89 | 1.05 (0.99, 1.12) | 0.118 | 1.04 (1.00, 1.09) | 0.052 | 1.02 (0.94, 1.11) | 0.638 | 1.05 (1.02, 1.09) | 0.001 |
|  | 90-99 | 1.20 (1.08, 1.33) | <0.001 | 1.13 (1.05, 1.22) | <0.001 | 1.00 (0.86, 1.15) | 0.980 | 1.12 (1.06, 1.18) | <0.001 |
|  | 100-109 | 1.46 (1.15, 1.85) | 0.002 | 1.43 (1.18, 1.73) | <0.001 | 1.15 (0.85, 1.57) | 0.360 | 1.18 (1.04, 1.35) | 0.011 |
|  | ≥110 | 1.78 (1.07, 2.96) | 0.025 | 0.95 (0.58, 1.56) | 0.850 | 1.05 (0.47, 2.34) | 0.911 | 1.46 (1.11, 1.93) | 0.007 |

The analysis is adjusted for calendar year at assessment, age, sex, diabetes duration, BMI, smoking, HbA1c, LDL cholesterol, HDL cholesterol, log triglycerides, presence of albuminuria, history of CKD, history of CVD and use of blood pressure lowering medications.

Multiple imputation was performed for covariates with missing data, including diabetes duration, BMI, HbA1c, LDL cholesterol, HDL cholesterol, and log-transformed triglycerides. Specifically, 25 imputed datasets were created, corresponding to the highest proportion of incomplete cases in the dataset, and the results were pooled using Rubin’s rules.

**Supplementary Table S23:** Cox regression for the association of 10 mmHg increase in diastolic blood pressure (DBP) and pulse pressure (PP) with incident events among individuals with DBP < 70 mmHg (n = 110,950).

| Age group | Single blood pressure model | Dual blood pressure model | Cardiovascular disease | | Chronic Kidney Disease | | Kidney failure | | All-cause death | |
| --- | --- | --- | --- | --- | --- | --- | --- | --- | --- | --- |
|  |  |  | HR/10 mmHg  (95% CI) | P-value | HR/10 mmHg  (95% CI) | P-value | HR/10 mmHg  (95% CI) | P-value | HR/10 mmHg  (95% CI) | P-value |
| 18-44 years | DBP |  | 0.85 (0.63, 1.14) | 0.285 | 1.20 (0.93, 1.53) | 0.154 | 1.52 (0.99, 2.34) | 0.054 | 0.78 (0.60, 1.01) | 0.060 |
|  | PP |  | 1.02 (0.91, 1.14) | 0.721 | 0.97 (0.88, 1.06) | 0.470 | 0.91 (0.78, 1.06) | 0.214 | 0.92 (0.82, 1.03) | 0.149 |
|  |  | DBP | 0.86 (0.63, 1.15) | 0.307 | 1.19 (0.93, 1.52) | 0.178 | 1.48 (0.96, 2.27) | 0.077 | 0.75 (0.57, 0.97) | 0.032 |
|  |  | PP | 1.01 (0.90, 1.13) | 0.858 | 0.98 (0.89, 1.07) | 0.588 | 0.93 (0.79, 1.08) | 0.334 | 0.90 (0.81, 1.01) | 0.082 |
| 45-59 years | DBP |  | 0.94 (0.85, 1.04) | 0.225 | 0.95 (0.89, 1.02) | 0.172 | 0.89 (0.77, 1.03) | 0.115 | 0.80 (0.74, 0.87) | <0.001 |
|  | PP |  | 1.08 (1.04, 1.11) | <0.001 | 1.04 (1.02, 1.07) | <0.001 | 1.07 (1.03, 1.12) | 0.002 | 1.02 (0.99, 1.04) | 0.260 |
|  |  | DBP | 0.96 (0.86, 1.06) | 0.415 | 0.97 (0.90, 1.03) | 0.319 | 0.90 (0.78, 1.04) | 0.164 | 0.80 (0.74, 0.87) | <0.001 |
|  |  | PP | 1.07 (1.04, 1.11) | <0.001 | 1.04 (1.02, 1.06) | <0.001 | 1.07 (1.02, 1.12) | 0.003 | 1.01 (0.98, 1.04) | 0.515 |
| 60-74 years | DBP |  | 0.91 (0.86, 0.97) | 0.002 | 0.92 (0.89, 0.95) | <0.001 | 0.99 (0.92, 1.07) | 0.844 | 0.86 (0.83, 0.90) | <0.001 |
|  | PP |  | 1.05 (1.03, 1.07) | <0.001 | 1.05 (1.04, 1.06) | <0.001 | 1.10 (1.08, 1.13) | <0.001 | 1.04 (1.03, 1.06) | <0.001 |
|  |  | DBP | 0.92 (0.87, 0.98) | 0.008 | 0.93 (0.90, 0.97) | <0.001 | 1.02 (0.94, 1.10) | 0.640 | 0.87 (0.84, 0.90) | <0.001 |
|  |  | PP | 1.05 (1.03, 1.07) | <0.001 | 1.05 (1.03, 1.06) | <0.001 | 1.10 (1.08, 1.13) | <0.001 | 1.04 (1.03, 1.05) | <0.001 |
| ≥75 years | DBP |  | 0.91 (0.85, 0.97) | 0.002 | 0.96 (0.92, 1.00) | 0.036 | 0.88 (0.81, 0.95) | <0.001 | 0.88 (0.86, 0.91) | <0.001 |
|  | PP |  | 1.05 (1.02, 1.07) | <0.001 | 1.04 (1.03, 1.06) | <0.001 | 1.11 (1.08, 1.14) | <0.001 | 1.01 (1.00, 1.02) | 0.219 |
|  |  | DBP | 0.92 (0.86, 0.98) | 0.006 | 0.97 (0.93, 1.01) | 0.102 | 0.89 (0.83, 0.96) | 0.003 | 0.88 (0.86, 0.91) | <0.001 |
|  |  | PP | 1.04 (1.02, 1.07) | <0.001 | 1.04 (1.03, 1.06) | <0.001 | 1.10 (1.08, 1.13) | <0.001 | 1.00 (0.99, 1.01) | 0.456 |
| All | DBP |  | 0.92 (0.88, 0.96) | <0.001 | 0.95 (0.92, 0.97) | <0.001 | 0.94 (0.89, 0.99) | 0.015 | 0.87 (0.85, 0.89) | <0.001 |
|  | PP |  | 1.05 (1.04, 1.06) | <0.001 | 1.05 (1.04, 1.06) | <0.001 | 1.09 (1.08, 1.11) | <0.001 | 1.02 (1.01, 1.03) | <0.001 |
|  |  | DBP | 0.93 (0.90, 0.97) | <0.001 | 0.96 (0.94, 0.98) | <0.001 | 0.96 (0.91, 1.01) | 0.102 | 0.87 (0.86, 0.89) | <0.001 |
|  |  | PP | 1.05 (1.03, 1.06) | <0.001 | 1.05 (1.04, 1.05) | <0.001 | 1.09 (1.08, 1.11) | <0.001 | 1.02 (1.01, 1.03) | <0.001 |

Effect of pulse pressure (PP) on the associations between diastolic blood pressure (DBP) and incident cardiovascular disease, chronic kidney disease, Kidney failure and all-cause death were examined by Cox proportional hazard model with PP and DBP included individually (single blood pressure model) and jointly (dual blood pressure model), adjusted for calendar year at assessment, age, sex, diabetes duration, BMI, smoking, HbA1c, LDL cholesterol, HDL cholesterol, log triglycerides, presence of albuminuria, history of CKD, history of CVD and use of blood pressure lowering medications.

**Supplementary Figure S1**: Study flow chart


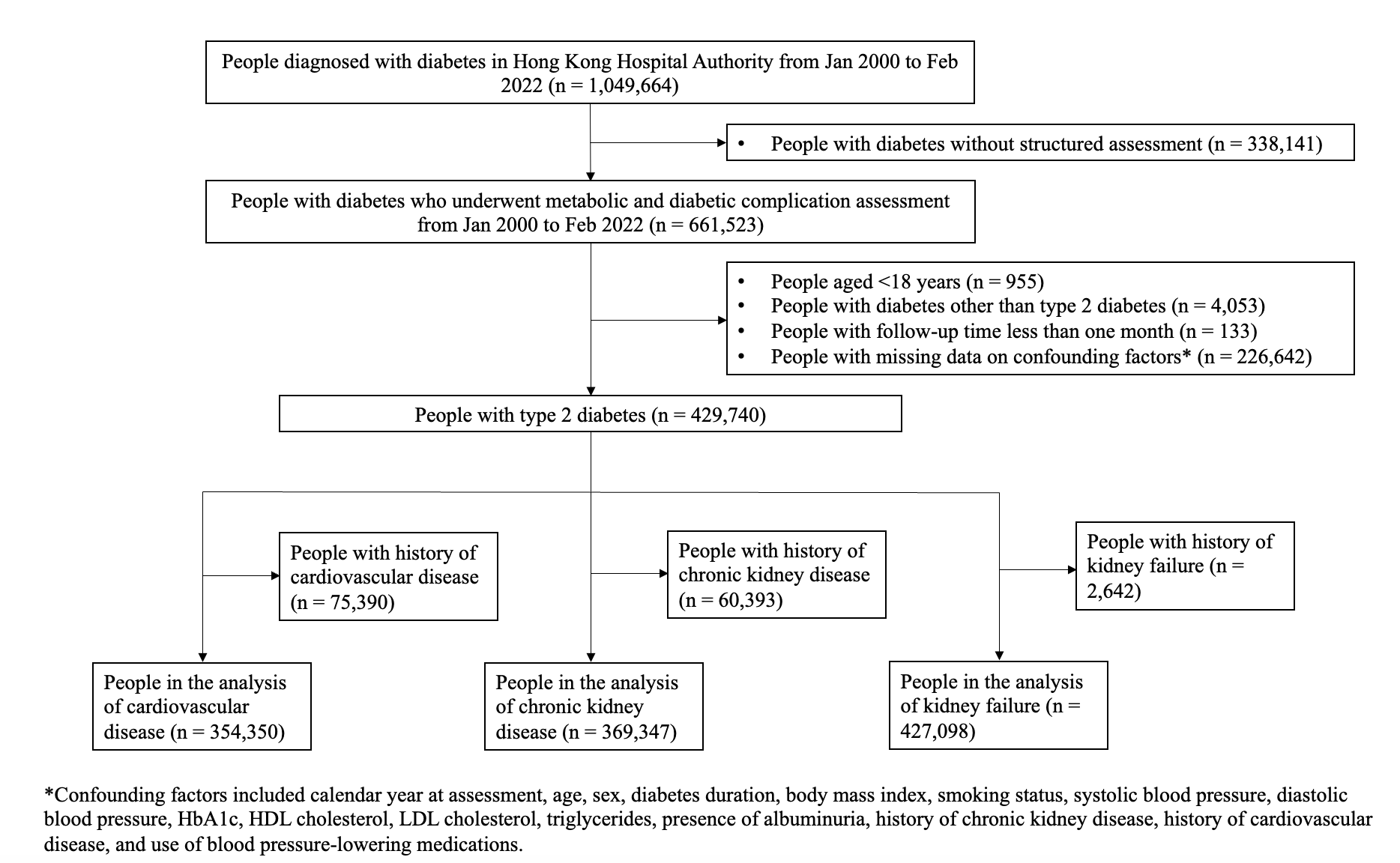


**Supplementary Figure S2:** Results of Cox proportional hazard model comparing different systolic blood pressure (SBP) and diastolic blood pressure (DBP) cut-offs referenced to SBP 120 – 129 mmHg and DBP 70 – 79 mmHg for incident events stratified by age categories. This analysis excluded individuals who had history of cardiovascular disease or chronic kidney disease, and those who developed cardiovascular disease, chronic kidney disease or all-cause death within 1 year of study entry.

Models were adjusted for calendar year at assessment, age, sex, diabetes duration, BMI, smoking, HbA1c, LDL cholesterol, HDL cholesterol, log triglycerides, presence of albuminuria and use of blood pressure lowering medications.


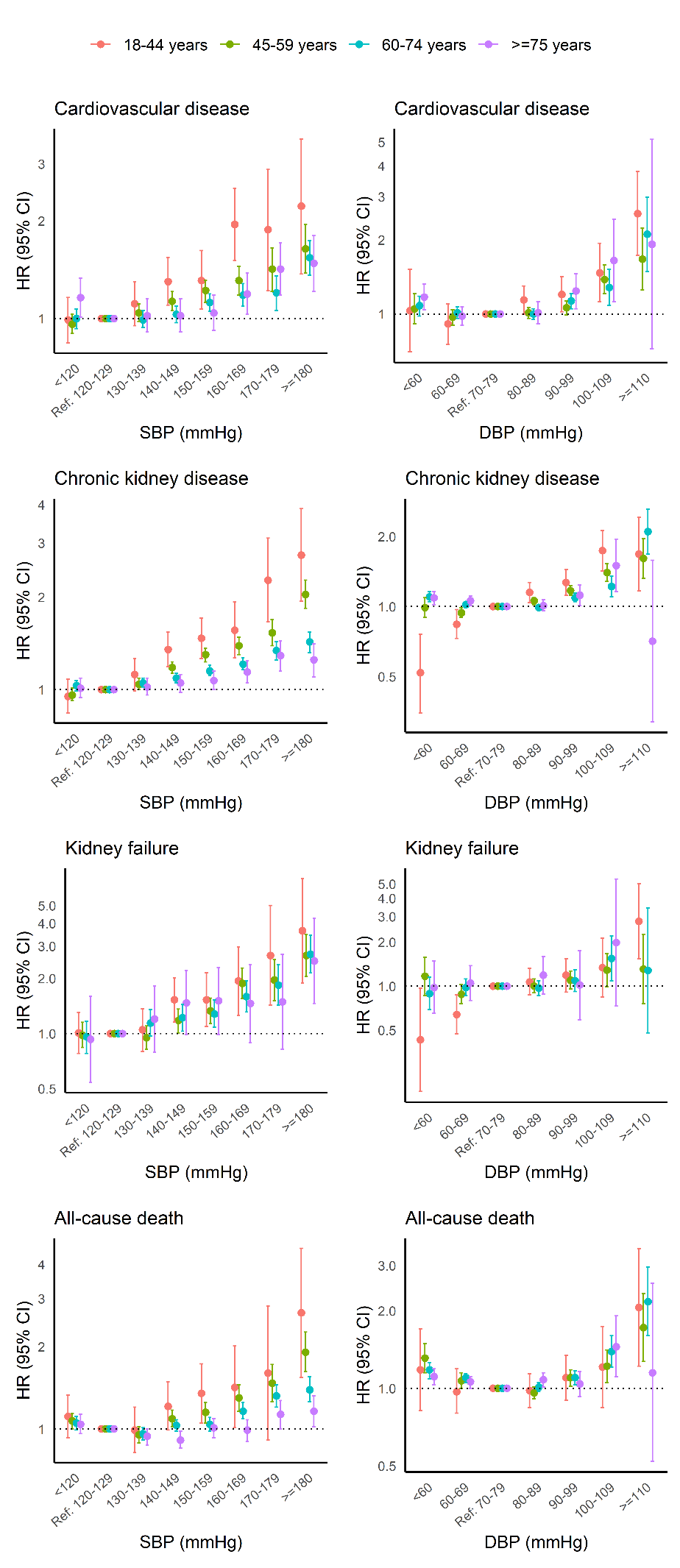

Supplement: Supplementary file 1 — Supplementary Material 1 [file 12933_2025_3072_MOESM1_ESM.docx]
